# *N1*-Benzyl Tryptamine Pan-SHIP1/2 Inhibitors: Synthesis and Preliminary Biological Evaluation as Anti-Tumor Agents

**DOI:** 10.3390/molecules27238451

**Published:** 2022-12-02

**Authors:** Sandra Fernandes, Shea T. Meyer, Jigisha P. Shah, Arijit A. Adhikari, William G. Kerr, John D. Chisholm

**Affiliations:** 1 Department of Microbiology & Immunology, SUNY Upstate Medical University, Syracuse, NY 13210, USA; 2 Department of Chemistry, Syracuse University, Syracuse, NY 13244, USA

**Keywords:** tryptamine, SHIP1, SHIP2, phosphatase, antitumor, medicinal chemistry

## Abstract

Inhibition of phosphatidylinositol 3,4,5-trisphosphate 5-phosphatase (SHIP) with small molecule inhibitors leads to apoptosis in tumor cells. Inhibitors that target both SHIP1 and SHIP2 (pan-SHIP1/2 inhibitors) may have benefits in these areas since paralog compensation is not possible when both SHIP paralogs are being inhibited. A series of tryptamine-based pan-SHIP1/2 inhibitors have been synthesized and evaluated for their ability to inhibit the SHIP paralogs. The most active compounds were also evaluated for their effects on cancer cell lines.

## 1. Introduction

**Figure 1:**
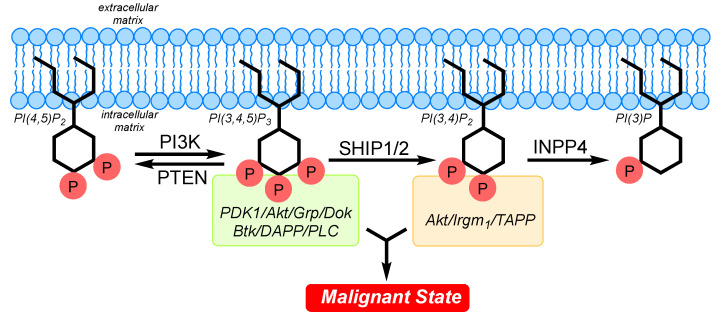
Malignancy and the “*Two PIP Hypothesis*”. Signaling an imbalance in the amounts of both PI(3,4,5)P_3_ and PI(3,4)P_2_ may lead to the development of a malignancy.

The PI3K pathway is a major cell signaling pathway in eukaryotic cells, having been shown to have a strong influence on cell division and cell survival. The pathway is named for the phosphoinositide 3-kinase (PI3K) enzyme (in this case referring specifically to the PIK3CA isoform) [1], which phosphorylates the membrane-anchored inositol phospholipid PI(4,5)P_2_ to form PI(3,4,5)P_3_. These membrane-anchored inositol phospholipids function as secondary messengers in the PI3K pathway. The diverse phosphorylation patterns present on the inositol ring of these lipids are recognized by PH domains present in signaling molecules such as serine/threonine kinases (such as Akt) which are typically activated upon binding to the inositol phospholipids and also by subsequent phosphorylation by other protein kinases (e.g., 3-phosphoinositide-dependent protein kinase-1, PDK1). This activation initiates a signaling cascade that transduces signals from the membrane to the cell nucleus. Signals from this pathway influence many cellular events, including cell division and survival. Modulating the concentration of the different inositol phospholipids on the interior of the plasma membrane has become a focus in the treatment of cancer, as controlling the amounts of these molecules may be used to curtail undesired cell division or survival. Inhibition of PI3K has been heavily investigated [2,3,4], with a number of clinically relevant PI3K inhibitors having been developed. Resistance to PI3K inhibitors has now been reported under some circumstances [5,6,7], so investigations into alternative approaches to influence PI3K signaling have been initiated. Modulation of the SH2-containing 5‘-inositol phosphatases SHIP1 and/or SHIP2, inositol phosphatases that degrade PI(3,4,5)P_3_, may provide another approach to sway PI3K pathway signaling. While SHIP is often considered as reversing signals initiated from PI3K phosphorylation, such as the well-known tumor promoter phosphatase and tensin homolog protein (PTEN) [8], the situation is more complex, with significant evidence pointing to SHIP1/2 facilitating tumor cell survival [9,10,11,12,13,14,15]. The primary difference between PTEN and the SHIP paralogs can be attributed to PTEN hydrolyzing the 3′ phosphate to generate PI(4,5)P_2_ from PI(3,4,5)P_3_, while SHIP1 and SHIP2 are 5′ phosphatases which convert PI(3,4,5)P_3_ to PI(3,4)P_2_. Therefore, SHIP and PTEN exert distinct effects on downstream signaling. Additionally, recent results show that the Akt2 isoform binds with greater affinity to PI(3,4)P_2_ [16], the product of the SHIP enzymes, instigating selective activation of Akt2 instead of activation of Akt1/Akt3 as is observed with PI(3,4,5)P_3_, the product of PI3K [17,18]. Therefore, the products of both SHIP1/2 and PI3K, PI(3,4)P_2_ and PI(3,4,5)P_3_, respectively, may be required to boost signals which lead to excessive cell division and promote survival in certain neoplasms. This has been termed the “*Two PIP Hypothesis*” where abnormal signaling from an imbalance in the amounts of both PI(3,4,5)P_3_ and PI(3,4)P_2_ may promote the development of the malignant state (Figure 1).

Consistent with the “*Two PIP Hypothesis*”, levels of the SHIP1 product PI(3,4)P_2_ are increased in several leukemia cells lines [19], while increased amounts of PI(3,4)P_2_ in inositol polyphosphate 4-phosphatase type II (INPP4A/B) knockout mice are implicated in promoting tumor formation [20,21]. We have also shown that the SHIP1 inhibitor 3α-aminocholestane (3AC) reduces Akt activation and promotes apoptosis of human blood cell cancers that express SHIP1, further supporting the hypothesis [9,14]. The key role of PI(3,4)P_2_ in cancer cell signaling was confirmed in these studies by demonstrating that the addition of PI(3,4)P_2_ onto leukemia cells is protective from apoptosis by SHIP1 inhibition in a dose-dependent fashion [9]. Related studies have shown that a SHIP1 agonist also slows the growth of multiple myeloma cells in vitro [22]. The observation that both agonists and antagonists of SHIP1 are cytotoxic to multiple myeloma cells highlights the delicate balance of PI(3,4,5)P_3_ and PI(3,4)P_2_ a cancer cell must maintain in order to develop and perpetuate the malignant state. Altogether a number of different breakdowns in PI3K signaling are possible in order for a cancer cell to reach a malignant state that satisfies the “*Two PIP Hypothesis*”.

There may also be applications for pan-SHIP1/2 inhibitors in the treatment of several types of cancer. As stated previously, a SHIP1 inhibitor showed cytotoxic effects on leukemia cells [9]. Other cancers may be more sensitive to SHIP2 inhibition, as SHIP2 expression is increased in breast cancer and promotes survival signals from epidermal growth factor receptor (EGF-R) in these tumors [11,13]. Further studies from our groups have shown that pan-SHIP1/2 inhibitors are also quite effective at slowing the growth of tumor cells [14,23]. Even though small molecule inhibitors are available for Akt [24], SHIP modulation provides a different molecular mechanism to influence PI3K signaling. This is advantageous for treating tumors that have become resistant to Akt inhibitors. The ability to produce a pan-SHIP1/2 inhibitor could prevent tumors from developing a resistance to inhibition of one SHIP paralog leading them to then utilize the other SHIP paralog to produce PI(3,4)P_2_. Such SHIP paralog compensation was in fact observed in vivo in a xenogeneic multiple myeloma model treated with the SHIP1-selective inhibitor 3α-aminocholestane (3AC). While 3AC was found to be effective in protecting the majority of mice from multiple myeloma growth; however, in those where there was relapse the tumor had up-regulated endogenous SHIP2 expression [14].

**Figure 2:**
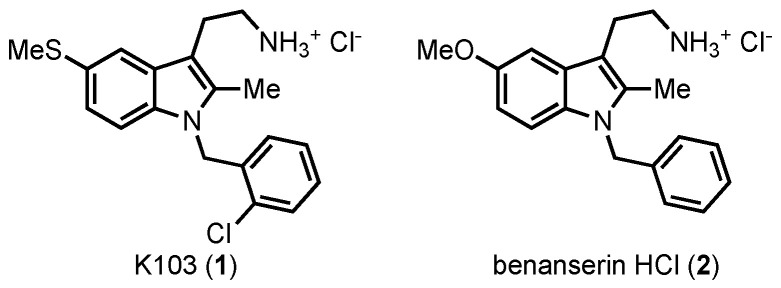
Structure of the pan-SHIP1/2 inhibitor K103 and Benanserin HCl.

A number of groups have reported the identification of small molecules that modulate SHIP activity [25,26,27]. Our own work in the area began with a high-throughput screen (HTS) used to identify molecules that inhibit recombinant SHIP1, which identified a number of lead compounds [9,14,28]. One inhibitor found from this screen was tryptamine K103 (**1**) (Figure 2), a close analog of the serotonin antagonist benanserin **2** [29,30].

K103 showed inhibition of both SHIP paralogs, marking it as a pan-SHIP1/2 inhibitor, but the molecule showed no effect on OCRL, another 5′ inositol phosphatase [14]. In line with the *“Two PIP Hypothesis”* the molecule demonstrated significant antitumor effects on several cell lines, especially on breast cancer cells [14,23]. Additional studies on K103 showed that inhibition of SHIP1/2 in multiple myeloma cells resulted in a G2/M cell cycle arrest, followed by caspase cascade activation leading to extensive apoptosis [14]. Tryptamine **1** is compliant with commonly used measures of druglike small molecule properties [31,32], but during the course of this work, it was discovered that **1** induced a psychotropic effect in mice which limited the usefulness of the molecule in vivo. Therefore, some synthetic studies on this tryptamine were initiated to define what features of the molecule were needed to maintain pan-SHIP1/2 inhibition so that an inhibitor with good pharmacodynamic properties and an improved side effect profile could be designed.

## 2. Results and Discussion

**Scheme 1:**
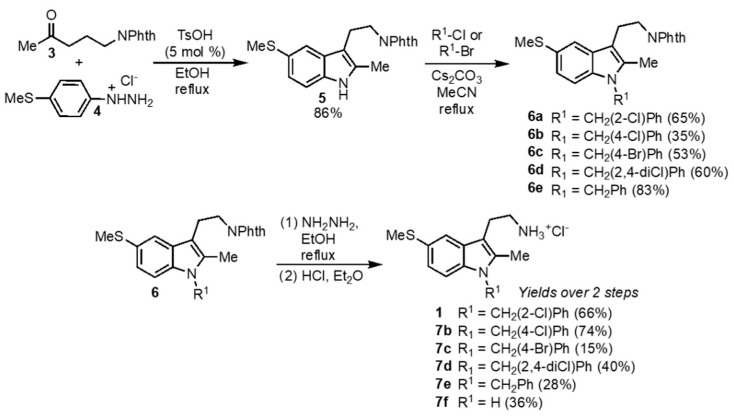
Synthesis of K103 and analogs.

The synthesis of K103 and related tryptamines was undertaken as shown in Scheme 1. The tryptamine core was accessed from a Fischer indole synthesis [33] with ketone **3** and hydrazine **4** as previously reported [34]. The indole nitrogen was then alkylated with benzyl bromides or benzyl chlorides using cesium carbonate in refluxing acetonitrile using the method of Fink [35]. Removal of the phthalimide protecting group with hydrazine and formation of the HCl salt of the resulting amine resulted in K103 **1** as well as the analogs **7b**–**7e**. A 5-thiomethyltryptamine with no benzyl group on the indole nitrogen (**7f**) was also prepared by deprotection of the phthalimide of **5** with hydrazine followed by the formation of the HCl salt.

**Scheme 2:**
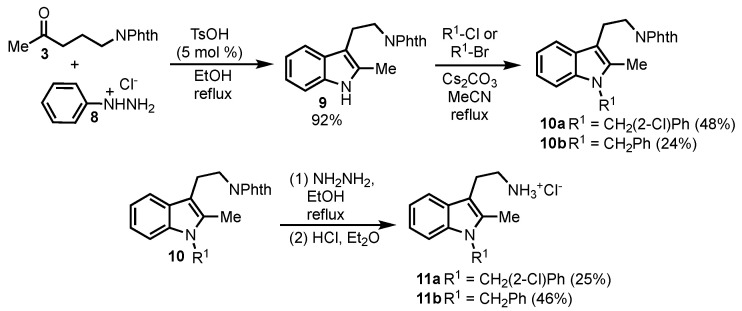
Synthesis of sulfur-free K103 analogs.

Analogs without the thiomethyl group on the tryptamine were also prepared (Scheme 2). The synthesis of these compounds employs phenylhydrazine hydrochloride **8** and ketone **3** in the Fischer indole reaction. Alkylation of the indole nitrogen in compound **9** with benzyl bromide or 2-chlorobenzylbromide gave the alkylated indoles **10a** and **10b,** respectively. These compounds were then deprotected leading to **11a** and **11b** after HCl salt formation.

**Scheme 3:**
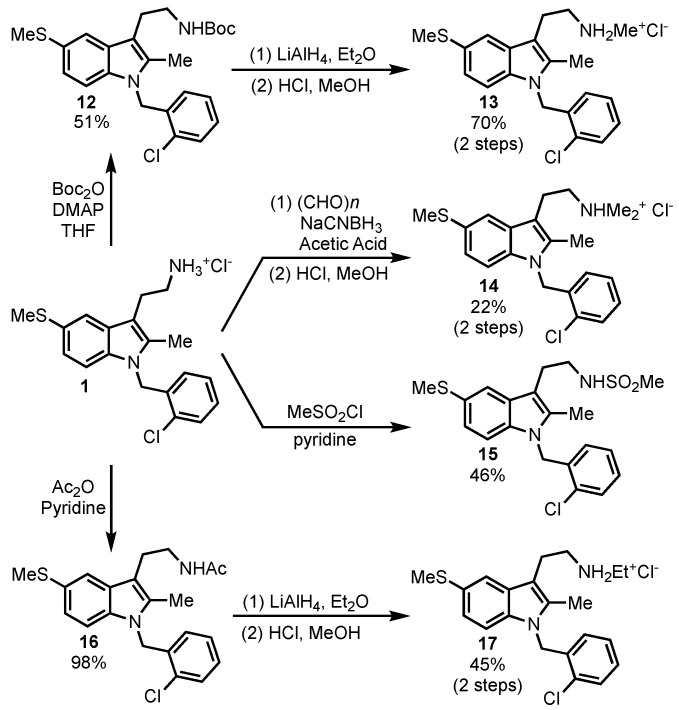
Modifications of the primary amine.

Substitution on the tryptamine primary amine was also briefly explored (Scheme 3). The primary amine was converted to methylsulfonamide **15** and acetamide **16** to determine if a basic nitrogen was needed for SHIP inhibition. Reduction of the amide **16** led to the ethylamine derivative **17**, while treatment of the parent compound with excess paraformaldehyde under reductive amination conditions gave the dimethylamino derivative **14**. The methylamine analog was also accessed by the formation of the Boc-protected amine **12**, followed by reduction to the methylamine with LiAlH_4_ and then the formation of the HCl salt **13**. This method provided more consistent results than reductive amination methods, which usually led to a mixture of starting material, methylamine and dimethylamine when one equivalent of paraformaldehyde was used.

These compounds were then tested for their activity against the two SHIP paralogs, SHIP1 and SHIP2 in the colorimetric malachite green phosphatase assay [36]. The malachite green assay detects the amount of free phosphate produced from enzymatic reactions with SHIP and an inositol phospholipid substrate [9]. The Malachite assay is useful for ruling out compounds that are fluorescent or have aggregation issues and is inexpensive, so it was utilized as the primary screen even though it is not as sensitive as other assays. Compounds that showed significant inhibition of SHIP in the Malachite had their IC_50_ against SHIP1 determined using a Fluorescence Polarization (FP) assay that we have previously described [9,14]. The FP assay is significantly more sensitive than the Malachite assay, and so this assay was used to determine IC_50_ of inhibitors that appeared active in the Malachite assay.

Testing of these tryptamines indicated that the halogen on the *N*-benzyl group seemed to improve inhibition of SHIP, as compounds **1**, **7b**–**7d** and **11b** showed significant activity while **7f** and **11a** showed virtually no activity against SHIP1 or SHIP2 (Table 1). Deletion of the thiomethyl group did allow for an active compound (**11b**), but the determination of the IC_50_ showed that there was some loss of activity when compared to **1**. Interestingly, returning the thiomethyl group to the 5-position of the indole along with the addition of an unsubstituted benzyl group did provide an active analog (**7e**), appearing to indicate that the thiomethyl is more responsible for activity than the halogen on the *N*-benzyl group. Modification of the *N*-benzyl group by moving the chloride to the 4-positon (**7b**) or using a 2,3-dichlorbenzyl (**7d**) provided analogs with significant activity, while the addition of a 4-bromobenzyl group gave less active analog.

Compounds with modifications of the primary amine of the tryptamine were also tested. Acylation was first explored, but acetamide **16** proved to be difficult to test, as it tended to precipitate when mixed with buffer. The methylsulfonamide **15** oddly proved to be a weak agonist of SHIP1. While SHIP1 agonists may have applications as anti-inflammatories [39,40], the agonist activity of **15** was weak and therefore was not further pursued. The addition of two methyl groups to the amine was also explored, with the tertiary amine salt **14** being inactive. Monoalkylation with either a methyl (**13**) or ethyl group (**17**) did lead to analogs with activity against SHIP2; however, no inhibition of SHIP1 was observed. While these analogs may present a path towards selective inhibitors of SHIP2, these compounds will not be useful in determining the effects of pan-SHIP inhibition in tumor models.

**Figure 3:**
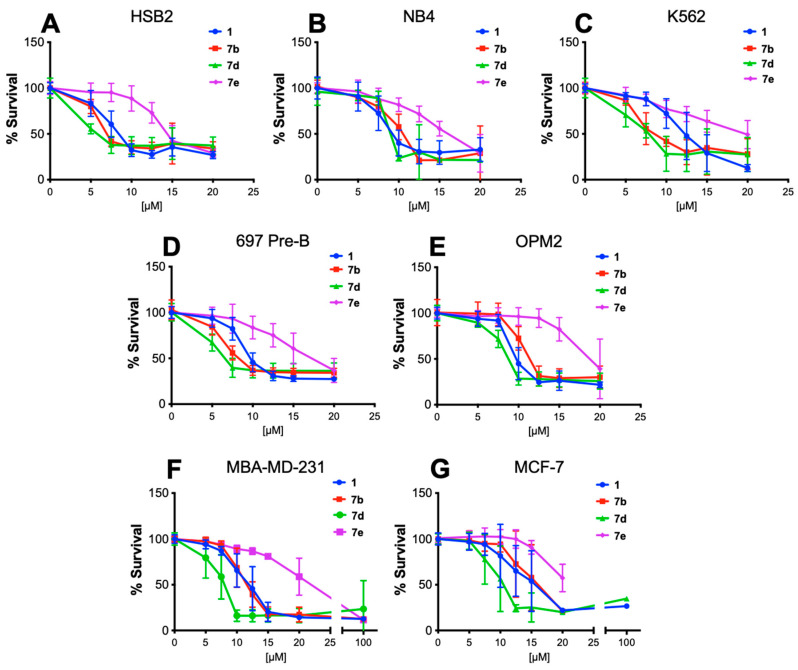
Activity of pan-SHIP1/2 Inhibitors against leukemia, multiple myeloma, and breast cancer cell lines. MTT assays show that some tryptamines kill tumor cell lines. Results are shown with connecting lines for clarity and expressed as mean ± standard error of the mean (SEM) of three individual experiments.

The most active pan-SHIP1/2 inhibitors (**1**, **7b**, **7d**, and **7e**) were then evaluated for cytotoxicity on several cancer cell lines using the MTT colorimetric assay for cell metabolic activity (Figure 3) [41]. The assay monitors the ability of NADPH-dependent cellular oxidoreductases to reduce the dye MTT (3-(4,5-dimethylthiazol-2-yl)-2,5-diphenyltetrazolium bromide), which when reduced produces a purple color that can be easily quantified. This assay is often used to determine cell viability [42]. Initially, the three new tryptamines and the parent compound **1** were evaluated on a number of leukemia cell lines (NB4, HSB2, K562 and 697-Pre-B) as well as the multiple myeloma cell line OPM-2. All of these cell lines express SHIP1 due to their hematopoietic origin, as SHIP1 is selectively expressed in blood and bone marrow cells [43]. Cytotoxicity was also found on the breast cancer cell lines MBA-MD-231 and MCF-7, which is likely due to the inhibition of SHIP2. SHIP2 is known to be overexpressed in breast cancer cells [11,13], and inhibition of SHIP2 has been shown to be cytotoxic to these cells previously [14,23]. Unexpectedly, the compound that exerted the greatest effect on these cell lines was **7d**, which in nearly every cell line exerted the greatest cytotoxicity. While not quite as active as **1** in the FP assay, **7d** is more hydrophobic with a ClogP of 4.6 vs. the ClogP of 4.11 for **1**. Being more hydrophobic, tryptamine **7d** may spend more time associated with the plasma membrane where SHIP activity is most important in signal transduction, and this may explain the greater influence on cells in the MTT assay.

## 3. Conclusions

In summary, an initial evaluation of some tryptamine pan-SHIP1/2 inhibitors has been conducted. The parent tryptamine and some analogous tryptamines have been synthesized and evaluated for activity against both SHIP paralogs in the Malachite Green assay. Several of these analogs showed significant activity against these inositol phosphatase enzymes. The most active analogs all contained chlorinated benzyl groups on the indole N1 nitrogen and a thiomethyl group at the 5-position on the indole. Some alkyl substitution was tolerated on the primary amine of the tryptamine, but disubstitution with alkyl groups or conversion to an amide or sulfonamide led significantly to reduced SHIP inhibition. The most active analogs showed good activity against a number of cancer cell lines. The analog that was best at killing cancer cells was not the most potent SHIP inhibitor, but this may be due to the lipophilicity of the compounds, as the most lipophilic compound was the most potent on cells and SHIP is known to be recruited the cell membrane. Future work will include efforts to further differentiate the structure from the tryptamine scaffold to avoid any psychotropic side effects.

## 4. Experimental

### 4.1. General Experimental Information

All anhydrous reactions were run under a positive pressure of argon. Dichloromethane (DCM) was dried by passage through an alumina column. 1,2-Dichloroethane (DCE) was freshly distilled from calcium hydride before use. Tetrahydrofuran (THF) was freshly distilled from Na/benzophenone still before use. Ethyl acetate (EA) and hexanes were purchased from commercial sources and used as received. Silica gel column chromatography was performed using 60 Å silica gel (230−400 mesh). Melting points are uncorrected. The 4-oxo-1-phthalimidopentane (**1**) [44] and the 4-(methylthio)phenylhydrazine hydrochloride (**2**) used in the indole synthesis were prepared as reported previously [45].

### 4.2. Synthesis of Protected Tryptamines ***5*** and ***9***

2-(2-(2-Methyl-5-(methylthio)-1H-indol-3-yl)ethyl)isoindoline-1,3-dione (**5**). 4-(Methylthio)phenylhydrazine hydrochloride **2** (1.96 g, 10.3 mmol) and 4-oxo-1-phthalimidopentane **3** (2.66 g, 11.5 mmol) were dissolved in ethanol (40 mL, 0.3 M). p-Toluenesulfonic acid monohydrate (3.51 g, 46.3 mmol) was added and the mixture was heated to reflux for 18 h. The mixture was then allowed to cool to room temperature and poured into 1 M aq. NaOH solution. The mixture was then extracted with DCM, and the organic extracts were dried over anhydrous Na_2_SO_4_, filtered and concentrated. The residue was purified by precipitating the desired tryptamine with EA yielding a yellow solid (3.100 g, 86%). TLC R_f_ = 0.34 (20% EA/80% hexanes); IR (ATR) 3337, 1706, 1394, 717 cm^−1^; ^1^H NMR (400 MHz, DMSO-*d_6_*) δ 10.79 (s, 1H), 7.79–7.84 (m, 4H), 7.37 (d, *J* = 1.8 Hz, 1H), 7.15 (d, *J* = 8.4 Hz, 1H), 6.94 (dd, *J* = 8.3, 1.8 Hz, 1H), 3.73 (t, *J* = 7.6 Hz, 2H), 2.94 (t, *J* = 7.3 Hz, 2H), 2.38 (s, 3H), 2.25 (s, 3H); ^13^C NMR (100 MHz, DMSO-*d_6_*) δ 168.3, 134.8, 134.3, 133.8, 132.0, 129.5, 126.1, 123.4, 121.9, 117.8, 111.6, 106.7, 38.5, 23.0, 18.3, 11.5. This compound has been previously reported [34].

2-(2-(2-Methyl-1H-indol-3-yl)ethyl)isoindoline-1,3-dione (**9**). Phenylhydrazine hydrochloride **8** (0.76 g, 7.72 mmol) and 4-oxo-1-phthalimidopentane **3** (2.00 g, 8.65 mmol) were dissolved in ethanol (3 mL, 0.3 M). p-Toluenesulfonic acid monohydrate (6.61 g, 34.75 mmol) was added to the mixture and heated to reflux for 18 h. The mixture was then allowed to cool to room temperature and poured into 1 M NaOH. The mixture was then extracted with DCM, and the combined organic extracts were dried over anhydrous Na_2_SO_4_, filtered and concentrated. The residue was purified by precipitating the desired tryptamine with EA yielding an orange powder (2.15 g, 92%). TLC R_f_ = 0.36 (30% EA/70% hexanes); IR (ATR) 3377, 3349, 2941, 1761, 1697, 1395, 711 cm^−1^; ^1^H NMR (400 MHz, DMSO-*d*_6_) δ 10.72 (s, 1H), 7.80–7.85 (m, 4H), 7.42 (d, *J* = 7.4 Hz, 1H), 7.21 (d, *J* = 7.8 Hz, 1H), 6.96 (d, *J* = 7.0 Hz, 1H), 6.90 (d, *J* = 7.6 Hz, 1H), 3.74 (t, *J* = 7.2 Hz, 2H), 2.96 (t, *J* = 7.3 Hz, 2H), 2.27 (s, 3H); ^13^C NMR (100 MHz, DMSO) δ 168.2, 135.6, 134.8, 132.8, 132.1, 128.6, 123.4, 120.5, 118.7, 117.4, 110.9, 106.8, 38.5, 23.2, 11.5. This compound has been previously reported [46].

### 4.3. General Procedure for N-Alkylation of Indoles

The indole (1 equiv) was dissolved in acetonitrile (0.1 M). Cs_2_CO_3_ (6 equiv) and the benzyl halide (3 equiv) were added and the mixture was heated to 80 °C for 18 h. The mixture was cooled to rt, and then filtered to remove the remaining Cs_2_CO_3_ and other salts. The filtrate was poured into brine and extracted with DCM. The combined organic extracts were dried over anhydrous Na_2_SO_4_, filtered, and concentrated. Silica gel chromatography then provided the desired products.

### 4.4. Tabulated Spectral Data for Protected Tryptamines ***6a**–**6e***, ***10a**–**10b***

2-(2-(1-(2-Chlorobenzyl)-2-methyl-5-(methylthio)-1H-indol-3-yl)ethyl)isoindoline-1,3-dione (**6a**). The crude oil was purified via silica gel chromatography with 100% chloroform yielding a yellow powder (2.65 g, 65%). TLC R_f_ = 0.43 (100% CHCl_3_); IR (ATR) 2936, 2929, 1707, 716 cm^−1^; ^1^H NMR (400 MHz, CDCl_3_) δ 7.79–7.82 (m, 2H), 7.67–7.69 (m, 2H), 7.64 (s, 1H), 7.39 (d, *J* = 8.0 Hz, 1H), 7.17 (t, *J* = 7.6 Hz, 1H), 7.07 (dd, *J* = 1.4, 8.4 Hz, 1H), 7.02 (t, *J* = 8.5 Hz, 2H), 6.19 (d, *J* = 8.5 Hz, 1H), 5.30 (s, 2H), 3.93 (t, *J* = 7.4 Hz, 2H), 3.12 (t, *J* = 7.4 Hz, 2H), 2.49 (s, 3H), 2.27 (s, 3H); ^13^C NMR (100 MHz, CDCl_3_) δ 168.3, 135.1, 135.0, 134.3, 133.8, 132.1, 131.8, 129.3, 128.8, 128.5, 127.7, 127.4, 126.9, 123.1, 123.0, 118.5, 109.5, 108.0, 44.4, 38.4, 23.5, 18.6, 10.0; Anal. Calcd for C_27_H_23_ClN_2_O_2_S: C, 68.27; H, 4.88; N, 5.90. Found: C, 68.34; H, 4.51; N, 6.18.

2-(2-(1-(4-Chlorobenzyl)-2-methyl-5-(methylthio)-1H-indol-3-yl)ethyl)isoindoline-1,3-dione (**6b**). The crude oil was purified via silica gel chromatography with 100% DCM yielding a yellow oil (0.470 g, 35%). TLC R_f_ = 0.63 (100% DCM); IR (ATR) 3038, 2920, 1702, 1027, 717 cm^−1^; ^1^H NMR (400 MHz, CDCl_3_) δ 7.77–7.79 (m, 2H), 7.66–7.69 (m, 2H), 7.63 (s, 1H), 7.21 (d, *J* = 7.7 Hz, 2H), 7.08 (d, *J* = 8.4 Hz, 1H), 7.03 (d, *J* = 8.4 Hz, 1H), 6.83 (d, *J* = 8.1 Hz, 2H), 5.20 (s, 2H), 3.90 (t, *J* = 7.4 Hz, 2H), 3.10 (t, *J* = 7.4 Hz, 2H), 2.49 (s, 3H), 2.26 (s, 3H); ^13^C NMR (100 MHz, CDCl_3_) δ 168.2, 136.2, 135.1, 134.2, 133.8, 133.1, 132.1, 128.9, 128.7, 127.7, 127.3, 123.1, 123.0, 118.6, 109.4, 108.0, 46.0, 38.4, 23.5, 18.7, 10.2. This compound has been reported previously [47].

2-(2-(1-(4-Bromobenzyl)-2-methyl-5-(methylthio)-1H-indol-3-yl)ethyl)isoindoline-1,3-dione (**6c**). The crude residue was purified via silica gel chromatography with 30% EA: 70% hexanes yielding an orange oil (0.780 g, 53%). TLC R_f_ = 0.43 (30% EA: 70% hexanes); IR (ATR) 2919, 2857, 1705, 1010, 715 cm^−1^; ^1^H NMR (400 MHz, CDCl_3_) δ 7.77–7.80 (m, 2H), 7.67–7.69 (m, 2H), 7.62 (s, 1H), 7.36 (d, *J* = 8.4 Hz, 2H), 7.08 (dd, *J* = 1.2, 8.4 Hz, 1H), 7.03 (d, *J* = 8.4 Hz, 1H), 6.76 (d, *J* = 8.3 Hz, 2H), 5.18 (s, 2H), 3.90 (t, *J* = 8.0 Hz, 2H), 3.10 (t, *J* = 8.0 Hz, 2H), 2.49 (s, 3H), 2.26 (s, 3H); ^13^C NMR (100 MHz, CDCl_3_) δ 168.2, 136.7, 135.0, 134.2, 133.8, 132.1, 131.9, 128.7, 127.7, 127.7, 123.1, 123.0, 121.1, 118.6, 109.4, 108.0, 46.0, 38.4, 23.5, 18.7, 10.1. This compound has been reported previously [47].

2-(2-(1-(2,4-Dichlorobenzyl)-2-methyl-5-(methylthio)-1H-indol-3-yl)ethyl)isoindoline-1,3-dione (**6d**). The crude oil was purified via silica gel column chromatography (80% hexanes: 20% EA) yielding a yellow solid (5.18 g, 60%). TLC R_f_ = 0.48 (80% hexanes: 20% EA), ^1^H NMR (400 MHz, CDCl_3_) δ 7.78–7.80 (m, 2H), 7.67–7.69 (m, 2H), 7.60 (d, *J* = 1.5 Hz, 1H), 7.40 (d, *J* = 2.0 Hz, 1H), 7.38 (d, *J* = 2.0 Hz, 1H), 7.02–7.09 (m, 2H), 6.10 (d, *J* = 8.4 Hz, 1H), 5.25 (s, 2H), 3.92 (t, *J =* 7.2 Hz, 2H), 3.12 (t, *J* = 7.3 Hz, 2H), 2.47 (s, 3H), 2.24 (s, 3H); ^13^C NMR (75 MHz, CDCl_3_) δ 168.3, 134.9, 134.0, 133.9, 133.7, 133.6, 132.3, 132.1, 131.8, 129.1, 128.8, 128.0, 127.9, 127.7, 123.1, 118.5, 109.3, 108.4, 44.0, 38.4, 23.5, 18.5, 10.0. HRMS (ESI) *m*/*z* calcd for C_27_H_23_Cl2N_2_O_4_S [M+H] 509.0851, found: 509.0850.

2-(2-(1-Benzyl-2-methyl-5-(methylthio)-1H-indol-3-yl)ethyl)isoindoline-1,3-dione (**6e**). The crude oil was purified via silica gel chromatography with 30% EA: 70% hexanes yielding a yellow oil (1.04 g, 83%). TLC R_f_ = 0.50 (30% EA: 70% hexanes); IR (ATR) 3028, 2917, 1703 cm^−1^; ^1^H NMR (400 MHz, CDCl_3_) δ 7.97–8.00 (m, 2H), 7.84–7.86 (m, 2H), 7.83 (s, 1H), 7.37–7.45 (m, 4H), 7.26 (s, 1H), 7.09 (d, *J* = 7.0 Hz, 2H), 5.43 (s, 2H), 4.08 (t, *J* = 7.9 Hz, 2H), 3.28 (t, *J* = 7.9 Hz, 2H), 2.68 (s, 3H), 2.48 (s, 3H); ^13^C NMR (100 MHz, CDCl_3_) δ 168.3, 137.7, 135.2, 134.4, 133.8, 132.1, 128.8, 128.7, 127.4, 127.3, 125.9, 123.1, 122.9, 118.6, 109.6, 107.7, 46.6, 38.4, 23.6, 18.7, 10.2. This compound has been reported previously [34].

2-(2-(1-(2-Chlorobenzyl)-2-methyl-1H-indol-3-yl)ethyl)isoindoline-1,3-dione (**10a**). The crude oil was purified via silica gel chromatography with 20% EA: 80% hexanes yielding a yellow solid (0.670 g, 48%). TLC R_f_ = 0.47 (20% EA: 80% hexanes); IR (ATR) 3300, 2918, 2860, 1704, 1042, 742 cm^−1^; ^1^H NMR (400 MHz, CDCl_3_) δ 7.81–7.83 (m, 2H), 7.68–7.72 (m, 3H), 7.39 (d, *J* = 8.2 Hz, 1H), 7.16 (t, *J* = 7.7 Hz, 1H), 7.08–7.11 (m, 3H), 7.02 (t, *J* = 7.7 Hz, 1H), 6.21 (d, *J* = 7.7 Hz, 1H), 5.34 (s, 2H), 3.95 (t, *J* = 8.2 Hz, 2H), 3.16 (t, *J* = 7.7 Hz, 2H), 2.26 (s, 3H); ^13^C NMR (100 MHz, CDCl_3_) δ 168.3, 136.3, 135.3, 133.8, 133.5, 132.2, 131.8, 129.3, 128.4, 128.0, 127.3, 126.9, 123.1, 121.2, 119.6, 118.1, 108.9, 108.2, 44.3, 38.5, 23.7, 9.9. This compound has been reported previously [47].

2-(2-(1-Benzyl-2-methyl-1H-indol-3-yl)ethyl)isoindoline-1,3-dione (**10b**). The crude oil was purified via silica gel chromatography with 20% EA: 80% hexanes yielding a yellow-white solid (0.310 g, 24%). TLC R_f_ = 0.26 (20% EA: 80% hexanes); IR (ATR) 3028, 2920, 2854, 1704, 714, 695 cm^−1^; ^1^H NMR (400 MHz, CDCl_3_) δ 7.81–7.83 (m, 2H), 7.68–7.70 (m, 3H), 7.16–7.25 (m, 4H), 7.08–7.10 (m, 2H), 6.93 (d, *J* = 7.0 Hz, 2H), 5.29 (s, 2H), 3.91 (t, *J* = 8.1 Hz, 2H), 3.12 (t, *J* = 8.1 Hz, 2H), 2.32 (s, 3H); ^13^C NMR (100 MHz, CDCl_3_) δ 168.3, 137.9, 136.5, 133.8, 133.6, 132.2, 128.7, 127.9, 127.2, 125.9, 123.1, 121.0, 119.3, 118.0, 109.0, 107.9, 46.5, 38.5, 23.7, 10.1. This compound has been reported previously [34].

### 4.5. General Procedure for Deprotection of the Phthalamide

To a solution of phthalimide-protected tryptamine (1 equiv) dissolved in methanol (0.3 M) was added hydrazine hydrate (5 equiv). The reaction mixture was brought to reflux at 75 °C for 30 min. The mixture was then allowed to cool, filtered, and concentrated under vacuum. The residue was purified via silica gel column chromatography using a 95% DCM: 5% methanol: 1% conc. aq. NH_4_OH. The resulting tryptamine was then dissolved in ethyl ether and added to a solution ethyl ether-hydrochloric acid (1 M) and allowed to stir for 30 min. The mixture was concentrated under vacuum and purified via recrystallization from ether/methanol.

2-(1-(2-Chlorobenzyl)-2-methyl-5-(methylthio)-1H-indol-3-yl)ethanaminium chloride (**1**). Recovered as white crystals (0.130 g, 74%). mp = 187–190 °C; IR (ATR) 2990, 2870, 2794, 1467, 1037, 752 cm^−1^; ^1^H NMR (400 MHz, CD_3_OD) δ 7.60 (s, 1H), 7.46 (d, *J* = 8.0 Hz, 2H), 7.23 (t, *J* = 7.8 Hz, 1H), 7.14 (s, 1H), 7.07 (t, *J* = 7.5 Hz, 1H), 6.23 (d, *J* = 7.5 Hz, 1H), 5.44 (s, 2H), 3.11–3.16 (m, 4H), 2.49 (s, 3H), 2.31 (s, 3H); ^13^C NMR (100 MHz, CD_3_OD) δ 135.5, 135.1 135.0, 131.6, 129.2, 128.5, 128.2, 128.2, 127.0, 126.5, 123.1, 118.1, 109.6, 105.7, 43.9, 39.8, 22.2, 17.4, 8.6; Anal. Calcd for C_19_H_22_Cl_2_N_2_S: C, 59.84; H, 5.81; N, 7.35. Found: C, 59.82; H, 5.71; N, 7.30.

2-(1-(4-Chlorobenzyl)-2-methyl-5-(methylthio)-1H-indol-3-yl)ethanaminium chloride (**7b**). Recovered as an off-white solid (0.047 g, 15%). IR (ATR) 2911, 2137, 1608, 1577, 1489, 1012, 794 cm^−1^; ^1^H NMR (400 MHz, CD_3_OD) δ 7.58 (s, 1H), 7.23–7.27 (m, 3H), 7.13 (d, *J* = 9.2 Hz, 1H), 6.94 (d, *J* = 7.4 Hz, 2H), 5.36 (s, 2H), 3.12 (bs, 4H), 2.48 (s, 3H), 2.34 (s, 3H); ^13^C NMR (100 MHz, CD_3_OD) δ 136.9, 135.5, 135.0, 132.7, 128.4, 128.2, 127.9, 127.4, 123.0, 118.1, 109.7, 105.5, 45.4, 39.8, 22.2, 17.4, 8.8. HRMS (ESI) *m*/*z* calcd for C_19_H_22_ClN_2_S [M+H] 345.1186, found: 345.1184.

2-(1-(4-Bromobenzyl)-2-methyl-5-(methylthio)-1H-indol-3-yl)ethanaminium chloride (**7c**). Recovered as an off-white solid (0.0113 g, 40%). IR (ATR) 2964, 2914, 2854, 1606, 1577, 1469, 1009, 791 cm^−1^; ^1^H NMR (400 MHz, CD_3_OD) δ 7.48 (d, *J* = 1.7 Hz, 1H), 7.30 (d, *J* = 8.4 Hz, 2H), 7.12 (d, *J* = 8.4 Hz, 1H), 7.03 (dd, *J* = 8.5, 1.8 Hz, 1H), 6.78 (d, *J* = 8.4 Hz, 2H), 5.24 (s, 2H), 2.97–3.06 (m, 4H), 2.38 (s, 3H), 2.23 (s, 3H); ^13^C NMR (100 MHz, CD_3_OD) δ 137.4, 135.5, 135.0, 131.4, 128.2, 127.9, 127.8, 123.0, 120.6, 118.1, 109.7, 105.5, 45.5, 39.8, 22.2, 17.4, 8.9. HRMS (ESI) *m*/*z* calcd for C_19_H_22_BrN_2_S [M+H] 389.0681, found: 389.0680.

2-(1-(2,4-Dichlorobenzyl)-2-methyl-5-(methylthio)-1H-indol-3-yl)ethanaminium chloride (**7d**). Recovered as an off-white solid (0.403 g, 28%). IR (ATR) 2981, 2878, 2811, 1610, 1587, 1564, 1415 cm^−1^; ^1^H NMR (400 MHz, CD_3_OD) δ 7.82 (s, 3H), 7.71 (d, *J* = 2.0 Hz, 1H), 7.57 (s, 1H), 7.24–7.28 (m, 2H), 7.05 (dd, *J* = 1.2, 8.2 Hz, 1H), 6.19 (d, *J* = 8.5 Hz, 1H), 5.41 (s, 2H), 2.94–3.01 (m, 4H), 2.49 (s, 3H), 2.25 (s, 3H); ^13^C NMR (100 MHz, CD_3_OD) δ 135.4, 134.9, 134.2, 133.5, 132.4, 128.9, 128.4, 128.2, 127.8, 127.3, 123.2, 118.1, 109.6, 105.9, 43.6, 39.8, 22.2, 17.3, 8.6; Anal. Calcd for C_19_H_22_Cl_2_N_2_S: C, 59.84; H, 5.81; N, 7.35. Found: C, 59.82; H, 5.71; N, 7.30.

2-(1-Benzyl-2-methyl-5-(methylthio)-1H-indol-3-yl)ethanaminium chloride (**7e**). Recovered as an off-white solid (0.0443 g, 36%). IR (ATR) 2965, 2908, 1605, 1578, 1470, 1020, 717 cm^−1^; ^1^H NMR (400 MHz, CD_3_OD) δ 7.57 (s, 1H), 7.21–7.28 (m, 4H), 7.14 (dd, *J* = 2.1, 8.5 Hz, 1H), 6.97 (d, *J* = 7.1 Hz, 2H), 5.38 (s, 2H), 3.107–3.16 (m, 4H), 2.48 (s, 3H), 2.34 (s, 3H); ^13^C NMR (100 MHz, CD_3_OD) δ 138.0, 135.6, 135.1, 128.3, 128.1, 127.7, 126.9, 125.8, 123.0, 118.1, 109.8, 105.2, 46.0, 39.9, 22.2, 17.5, 8.8. This compound has been reported previously [34].

2-(2-Methyl-5-(methylthio)-1H-indol-3-yl)ethanaminium chloride (**7f**). Recovered as a brown plate-like solid (0.124 g, 66%). IR (ATR) 2980, 2915, 2162,1458, 1432, 1294, 797 cm^−1^; ^1^H NMR (400 MHz, CD_3_OD) δ 7.52 (d, *J* = 1.6 Hz, 1H), 7.22 (d, *J* = 8.4 Hz, 1H), 7.04 (dd, *J* = 8.4, 1.6 Hz, 1H), 3.05–3.11 (m, 4H), 2.44 (s, 3H), 2.38 (s, 3H); ^13^C NMR (100 MHz, CD_3_OD) δ 134.6, 133.9, 128.8, 126.8, 122.5, 117.9, 111.0, 104.4, 39.9, 21.9, 17.7, 10.2. This compound has been reported previously [34].

2-(1-(2-Chlorobenzyl)-2-methyl-5-1H-indol-3-yl)ethanaminium chloride (**11a**). Recovered as an off-white solid (0.130 g, 25%). IR (ATR) 2980, 2970, 1736, 1591, 1570, 1469, 743 cm^−1^; ^1^H NMR (400 MHz, CD_3_OD) δ 7.58–7.60 (m, 1H), 7.45 (d, *J* = 7.7 Hz, 1H), 7.16–7.25 (m, 2H), 7.03–7.10 (m, 3H), 6.22 (d, *J* = 7.2 Hz, 1H), 5.45 (s, 2H), 3.17 (bs, 4H), 2.32 (s, 3H); ^13^C NMR (100 MHz, CD_3_OD) δ 140.6, 139.2, 137.9, 135.6, 133.1, 132.3, 131.4, 130.9, 130.5, 125.1, 123.3, 121.1, 112.8, 109.8, 47.7, 43.8, 26.2, 12.5. HRMS (ESI) *m*/*z* calcd for C_18_H_20_ClN_2_ [M+H] 299.1309, found: 299.1308.

2-(1-Benzyl-2-methyl-1H-indol-3-yl) ethanaminium chloride (**11b**). Recovered as an off-white solid (0.1080 g, 46%). IR (ATR) 3026, 2913, 1604, 1568, 1467, 733 cm^−1^; ^1^H NMR (400 MHz, CD_3_OD) δ 7.53 (d, *J* = 6.8 Hz, 1H), 7.20–7.30 (m, 4H), 7.04–7.12 (m, 2H), 6.97 (d, *J* = 7.2 Hz, 2H), 5.40 (s, 2H), 3.12–3.15 (m, 4H), 2.35 (s, 3H); ^13^C NMR (100 MHz, CD_3_OD) δ 138.2, 136.8, 134.1, 128.3, 127.3, 126.8, 125.8, 120.9, 119.1, 116.9, 109.1, 105.4, 45.9, 39.9, 22.3, 8.8. HRMS (ESI) *m*/*z* calcd for C_18_H_21_N_2_ [M+H] 265.1699, found: 265.1697.

### 4.6. Modification of the Tryptamine Primary Amine

*tert*-Butyl-N-(2{1-[(2-chlorophenyl)methyl]-2-methyl-5-(methylsulfanyl)-1H-indol-3-yl}ethyl)carbamate (**12**). Tryptamine **1** (0.200 g, 0.530 mmol) was dissolved in 3 mL of 2:1 methanol/THF. Triethylamine (0.081 mL, 0.53 mmol) and di-*tert*-butyl dicarbonate (0.108 g, 0.57 mmol) were added to the mixture. After 4 h at rt the solvent was removed via rotary evaporation and the residue was taken up in EA. The solution was washed with sat. NaHCO_3_ (3 × 15 mL). The organic layer was collected, dried over anhydrous Na_2_SO_4_, filtered and concentrated. The residue was purified via silica gel chromatography using a 90% hexanes: 10% EA eluent yielding carbamate **12** as a foamy white solid (0.120 g, 51%). TLC R_f_ = 0.25 (10% EA: 90% hexanes); IR (ATR) 3350, 2975, 1692, 1162 cm^−1^; ^1^H NMR (400 MHz, CDCl_3_) δ 7.57 (s, 1H), 7.40 (d, *J* = 8.0 Hz, 1H), 7.13–7.19 (m, 2H), 7.01–7.08 (m, 2H), 6.21 (d, *J* = 7.7 Hz, 1H), 5.33 (s, 2H), 4.56 (bs, 1H), 3.37 (bs, 2H), 2.94 (t, *J* = 6.5 Hz, 2H), 2.52 (s, 3H), 2.27 (s, 3H), 1.43 (s, 9H); ^13^C NMR (100 MHz, CD_3_CN) δ 156.4, 136.2, 135.7, 135.5, 132.2, 129.9, 129.7, 129.3, 128.0, 127.9, 127.4, 122.8, 119.1, 110.2, 109.6, 78.5, 44.7, 41.5, 28.2, 25.2, 18.2, 9.9. HRMS (ESI) *m*/*z* calcd for C_24_H_30_ClN_2_O_2_S [M+H] 445.1711, found: 445.1708.

(2{1-[(2-Chlorophenyl)methyl]-2-methyl-5-(methylsulfanyl-1H-indol-3-yl}ethyl)methylaminium chloride (**13**). Carbamate **12** (0.200 g, 0.450 mmol) was dissolved in dry THF (5 mL, 0.1 M) and cooled to 0 °C. LiAlH_4_ solution (1 M in THF, 1.92 mL, 1.92 mmol) was added slowly to the mixture at 0 °C. The reaction was allowed to warm to rt and then heated to reflux for 4h. After cooling the reaction to rt, the mixture was diluted with 10 mL of ethyl ether and quenched by the sequential addition of 80 µL of water, 240 µL of 15% aq. NaOH solution, and 80 µL of water. Approximately 1 g of anhydrous MgSO_4_ was then added and, after stirring for 15 min, the mixture filtered through celite with ethyl ether and concentrated. The residue was purified via silica gel chromatography using a solvent gradient of 100% EA to 95% EA: 5% NH_4_OH yielding the methylamine derivative. This was then added to a 1:1 ether-hydrochloric acid solution (1 mL, 2 M) and allowed to stir for 30 min. The mixture was concentrated under vacuum and purified via recrystallization from ether/methanol yielding **13** as a red solid (0.124 g, 70% from **12**). IR (ATR) 2917, 2696, 2431, 1466, 1441, 1038, 751 cm^−1^; ^1^H NMR (400 MHz, CDCl_3_) δ 7.64 (s, 1H), 7.42 (d, *J* = 7.8 Hz, 1H), 7.19 (d, *J* = 7.8 Hz, 1H), 7.07 (s, 1H), 7.02 (t, *J* = 7.8 Hz, 1H), 6.20 (d, *J* = 7.4 Hz, 1H), 5.36 (s, 2H), 3.11–3.23 (m, 4H), 2.74 (s, 3H), 2.47 (s, 3H), 2.29 (s, 3H); ^13^C NMR (100 MHz, CD_3_OD) δ 135.5, 135.1, 131.7, 129.2, 128.5, 128.2, 128.1, 127.0, 126.5, 123.1, 118.1, 111.4, 109.6, 105.3, 49.4, 43.9, 32.4, 21.1, 17.4, 8.6. HRMS (ESI) *m*/*z* calcd for C_20_H_24_ClN_2_S [M+H] 359.1343, found: 359.1340.

(2{1-[(2-Chlorophenyl)methyl]-2-methyl-5-(methylsulfanyl-1H-indol-3-yl}ethyl) dimethylaminium chloride (**14**). Tryptamine **1** (0.175 g, 0.460 mmol) was dissolved in 0.60 mL of acetic acid. Sodium cyanoborohydride (0.289 g, 4.6 mmol) dissolved in 1 mL of methanol was added to the reaction followed by the addition of formalin (37%, 1.8 mL, 23 mmol). The mixture was stirred at rt for 18 h, followed by solvent removal in vacuo. The residue was dissolved in DCM and the mixture was washed using 2 M aq. NaOH solution (3 × 5 mL). The organic layer was then dried over anhydrous Na_2_SO_4_, filtered, and concentrated. The crude oil was purified via silica gel chromatography using a 90% DCM: 10% MeOH eluent yielding a clear oil that was then added to a 1:1 ether-hydrochloric acid solution (1 mL, 2 M) and allowed to stir for 30 min. The mixture was concentrated under vacuum and purified via recrystallization from ether/methanol yielding **14** a red solid (0.040 g, 21% over 2 steps). IR (ATR) 2917, 2780, 2665, 2469, 1466, 1441, 1413, 1048, 750 cm^−1^; ^1^H NMR (400 MHz, CDCl_3_) δ 12.94 (bs, 1H), 7.55 (s, 1H), 7.41 (d, *J* = 7.8 Hz, 1H), 7.15–7.21 (m, 2H), 7.01–7.09 (m, 2H), 6.17 (d, *J* = 7.5 Hz, 1H), 5.33 (s, 2H), 3.38–3.42 (m, 2H), 3.20 (m, 2H), 2.91 (s, 3H), 2.89 (s, 3H), 2.55 (s, 3H), 2.36 (s, 3H); ^13^C NMR (100 MHz, CD_3_OD) δ 135.3, 135.2, 134.6, 131.9, 129.5, 128.7, 128.3, 127.8, 127.3, 126.6, 123.4, 118.2, 110.0, 105.3, 58.1, 44.5, 42.9, 20.2, 18.8, 10.3. HRMS (ESI) *m*/*z* calcd for C_21_H_26_ClN_2_S [M+H] 373.1499, found: 373.1496.

*N*-(2{1-[(2-Chlorophenyl)methyl]-2-methyl-5-(methylsulfanyl)-1H-indol-3-yl}ethyl) methanesulfonamide (**15**). Tryptamine **1** (0.250 g, 0.530 mmol) was dissolved in 0.6 mL of DCM and cooled to −78 °C. Triethylamine (0.074 mL, 0.527 mmol) was added to the mixture and, after 5 min, methanesulfonyl chloride (0.041 mL, 0.527 mmol) dissolved in 0.6 mL of DCM was added. The mixture was kept at −78 °C for 2 h, and then warmed to rt and diluted with water. The mixture was extracted using DCM (3 × 5 mL) and the combined organic layers were washed with sat. aq. NaCl solution. The organic layer was then dried over anhydrous Na_2_SO_4_, filtered and concentrated. The residue was purified via silica gel chromatography (30% EA: 70% hexanes) yielding sulfonamide **15** a white powder (0.091 g, 46%). TLC R_f_ = 0.22 (30% EA: 70% hexanes); IR (ATR) 3659, 3287, 2980, 2919, 1463, 1440, 1313, 1145, cm^−1^; ^1^H NMR (400 MHz, CDCl_3_) δ 7.59 (s, 1H), 7.43 (d, *J* = 7.3 Hz, 1H), 7.16–7.20 (m, 2H), 7.06–7.11 (m, 2H), 6.24 (d, *J* = 7.3 Hz, 1H), 5.36 (s, 2H), 4.42 (bs, 1H), 3.43–3.41 (m, 2H), 3.09–3.05 (m, 2H), 2.89 (s, 3H), 2.55 (s, 3H), 2.33 (s, 3H); ^13^C NMR (100 MHz, CD_3_OD) δ 135.3, 135.0, 134.9, 131.9, 129.5, 128.7, 128.4, 128.0, 127.4, 126.7, 123.3, 118.6, 109.8, 107.3, 44.5, 43.6, 40.4, 25.7, 18.7, 10.2. HRMS (ESI) *m*/*z* calcd for C_20_H_24_ClN_2_O_2_S_2_ [M+H] 423.0962, found: 423.0960.

*N*-(2{1-[(2-Chlorophenyl)methyl]-2-methyl-5-(methylsulfanyl-1H-indol-3-yl}ethyl) acetamide (**16**). Tryptamine **1** (0.120 g, 0.314 mmol) was dissolved in 1.5 mL of 2:1 methanol/THF and cooled to 0 °C. Triethylamine (0.086 mL, 0.692 mmol) was added to the mixture and stirred 5 min. Acetic anhydride (0.033 mL, 0.346 mmol) was added and the reaction was brought to rt and stirred for 2 h. The mixture was then diluted in EA and washed with sat. aq. NaHCO_3_ (3 × 15 mL). The organic layer was dried over anhydrous Na_2_SO_4_, filtered and concentrated. The residue was purified via silica gel chromatography (40% EA: 60% hexanes) yielding acetamide **16** as a red foamy solid (0.110 g, 98%). TLC R_f_ = 0.33 (40% EA: 60% hexanes); IR (ATR) 3249, 3067, 2919, 1630, 1468,1368, 752 cm^−1^; ^1^H NMR (400 MHz, CDCl_3_) δ 7.57 (s, 1H), 7.40 (d, *J* = 8.0 Hz, 1H), 7.12–7.19 (m, 2H), 7.00–7.07 (m, 2H), 6.19 (d, *J* = 7.7 Hz, 1H), 5.79 (bs, 1H), 5.32 (s, 2H), 3.50 (q, *J* = 6.3 Hz, 2H), 2.96 (t, *J* = 6.8 Hz, 2H), 2.51 (s, 3H), 2.26 (s, 3H), 1.92 (s, 3H); ^13^C NMR (100 MHz, CDCl_3_) δ 170.2, 135.3, 135.0, 134.5, 131.9, 129.5, 128.7, 128.6, 127.7, 127.3, 126.7, 123.2, 118.9, 109.7, 108.7, 44.5, 40.3, 24.5, 23.3, 18.8, 10.0. HRMS (ESI) *m*/*z* calcd for C_21_H_24_ClN_2_OS [M+H] 387.1292, found: 387.1290.

(2{1-[(2-Chlorophenyl)methyl]-2-methyl-5-(methylsulfanyl-1H-indol-3-yl}ethyl) ethylaminium chloride (**17**). Acetamide **16** (0.37 mmol, 150 mg) was dissolved in dry THF (5 mL, 0.1 M) and cooled to 0 °C. LiAlH_4_ (1 M in THF, 2.0 mL, 2.0 mmol) was added slowly to the mixture at 0 °C. The reaction was warmed to rt and then heated to reflux for 4h. After cooling the reaction to rt, the mixture was diluted with 10 mL of ethyl ether and quenched by the sequential addition of 100 µL of water, 300 µL of 15% aq. NaOH solution, and 100 µL of water. Approximately 1 g of anhydrous MgSO_4_ was then added and, after stirring for 15 min, the mixture filtered through celite with ethyl ether and concentrated. The residue was then purified via silica gel chromatography using (90% DCM: 9% methanol: 1% conc. aq. NH_4_OH) to provide the corresponding ethylamine as a yellow oil (63%). This oil was then added to a 1:1 ether-HCl acid solution (1 mL, 2 M) and allowed to stir for 30 min. The mixture was concentrated under vacuum and purified via recrystallization from ether/methanol yielding **17** as a white solid. mp = 196–198 °C (MeOH). IR (thin film) 3443, 2908, 2900, 1551, 1375, 1242 cm^−1^; ^1^H NMR (300 MHz, CDCl_3_) δ 7.61 (s. 1H), 7.38 (m, 1H), 7.11–7.19 (m, 2H), 6.98–7.06 (m, 2H), 6.18 (d, *J* = 7.6 Hz*, * 1H), 5.31 (s, 2H), 3.10 (bs, 2H), 3.03 (t, *J* = 6.5 Hz, 2H), 2.93 (t, *J* = 6.2 Hz, 2H), 2.75 (q, *J* = 7.1 Hz, 2H), 2.52 (s, 3H), 2.28 (s, 3H), 1.11–1.04 (t, 3H); ^13^C NMR (75 MHz, CDCl_3_) δ 135.4, 135.2, 134.7, 134.5, 132.0, 129.6, 128.9, 128.6, 127.9, 127.6, 126.9, 123.3, 118.6, 110.0, 52.9, 46.6, 44.6, 29.9, 21.0, 19.0, 10.5. HRMS (ESI) *m*/*z* calcd for C_21_H_26_ClN_2_S [M+H] 373.1499, found: 373.1495.

### 4.7. Malachite Green Phosphatase Release Assays

Malachite green assays to monitor phosphatase release (Echelon Biosciences) were performed with recombinant human truncated SHIP1 (tSHIP1) [48] and SHIP2 (Echelon Biosciences). Serial dilutions of the compounds dissolved in DMSO were added to the recombinant enzymes diluted in reaction buffer Rx (50 mM Hepes pH 7.4, 150 mM NaCl, 1 mM MgCl_2_, 0.25 mM EDTA) in triplicate reaction wells in 96-well plates. After incubation for 2 min at room temperature, 2.5 µL of 1 mM phosphatidylinositol 3,4,5-trisphosphate diC8 (PI(3,4,5)P_3_-diC8, Echelon Biosciences) was added to each reaction to a final concentration of 100 µM in a final volume of 25 µL /well. Following a 20 min incubation at 37 °C, 100 µL of Malachite Green solution (Echelon Biosciences) was added to each well and the plates were incubated at room temperature in the dark for 15 min. Plates were then read at 620 nm on a plate reader (Synergy 2, BioTek, Santa Clara, CA, USA). In addition, 3α-Aminocholestane, a known SHIP1 inhibitor, and AS1949490, a known SHIP2 inhibitor, were used as positive controls.

### 4.8. Fluorescence Polarization (FP) Assays for the Detection of PI(3,4)P_2_

The FP Assay [49] for the detection of PI(3,4)P_2_ (Echelon Biosciences) was performed using tSHIP1 [48] with serial dilutions of the tryptamines in DMSO according to the manufacturer’s recommendation as previously described [14]. Briefly, serial dilution of the compounds and control with 2.5% solvent only (0 µM) is added to the enzyme in a volume of 40 µL of enzyme-specific reaction buffer (Rx) (S1 Reaction buffer for tSHIP1: 20 mM Tris pH 7.5, 150 mM NaCl, 0.05% Tween 20, 10 mM MgCl_2_), (S2 reaction buffer for tSHIP1 or SHIP2-Enz: 50 mM Hepes pH 7.4, 150 mM NaCl, 1mM MgCl_2_, 0.25 mM EDTA). 40 µL of 4 µM PIP_3_ (PI(3,4,5)P_3_-diC8) in Rx buffer is added to each reaction and incubated at 37 °C for 20 min, followed by heat inactivation for 3 min at 95 °C. Reactions were spun down briefly and 10 µL of each reaction was added in 6 replicate wells to a black 384-well plate provided with the FP kit. 10 µL of 1X reconstituted Detector (a phosphoinositide binding protein) in PBS is then added to each well, followed by 5 µL of 1X freshly diluted Probe (in PBS, a fluorophore-labeled phosphoinositide) protected from light. Assay plates are gently tapped to mix, the plate is sealed and spun down briefly and the reaction is allowed to equilibrate for 60 min at room temperature protected from light. Control wells are included with each assay (no enzyme control (NE): 5µL Rx buffer with 2.5% solvent + 5 µL PIP_3_ (4 µM), 10 µL Detector, 5 µL Probe, Probe Alone (PA) control (10 µL enzyme buffer, 10 µL PBS, 5 µL Probe). Fluorescence polarization is then read on a Bio-Tek Synergy 2 (BioTek, Santa Clara, CA, USA) plate reader and is expressed in milli Polarization (mP) units. 3α-Aminocholestane, a known SHIP1 inhibitor, and AS1949490, a known SHIP2 inhibitor, were used as positive controls.

### 4.9. MTT Cell Viability Assay

Cells were treated in triplicate or more with increasing concentrations of the compound. Cell viability was determined with a Cell Counting Kit (Dojindo Molecular Technologies, Rockville, MD, USA) per the manufacturer’s instructions. The odds density (OD) of compound-treated cells was divided by the OD of their vehicle control, and the viability was expressed as a percentage of untreated cells. Results are expressed as mean ± standard error of the mean (SEM) of three individual experiments. HSB2 (ATCC CCL-120.1), K562 (ATCC CCL-243), MBA-MD-231 (ATCC HTB-26), and MCF-7 (ATCC HTB-22) cell lines were obtained from the ATCC (American Type Culture Collection, Manassas, VA, USA), while the 697 Pre-B (ACC 42), NB-4 (ACC 207), and OPM-2 (ACC-50) cell lines were obtained from the DSMZ (German Collection of Microorganisms and Cell Cultures, Göttingen, Germany). Both the ATCC and DSMZ perform genetic validation on deposited cell lines.

## Figures and Tables

**Table 1:**
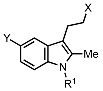
In Vitro Evaluation of Tryptamines.

Entry	Cmpd	R^1^	X	Y	CLogP ^b^	Malachite (500 µM) % Inhib. SHIP1	Malachite (250 µM) % Inhib. SHIP1	IC_50_ SHIP1 (Via FP)	Malachite (500 µM) % Inhib. SHIP2	Malachite (250 µM) % Inhib. SHIP2	IC_50_ SHIP2 (via FP)
1	**1**	2-chlorobenzyl	NH_3_^+^ Cl^−^	SMe	4.11	51%	7%	5 µM	59%	7%	10 µM
2	**7b**	4-chlorobenzyl	NH_3_^+^ Cl^−^	SMe	4.11	33%	0%	11 µM	49%	18%	14 µM
3	**7c**	4-bromobenzyl	NH_3_^+^ Cl^−^	SMe	4.23	47%	3%	51 µM	38%	30%	58 µM
4	**7d**	2,4-dichlorobenzyl	NH_3_^+^ Cl^−^	SMe	4.60	52%	32%	30 µM	58%	50%	30 µM
5	**7e**	benzyl	NH_3_^+^ Cl^−^	SMe	2.10	40%	0%	13 µM	39%	28%	12 µM
6	**7f**	H	NH_3_^+^ Cl^−^	SMe	3.62	0%	0%	-	2%	0%	-
7	**11a**	benzyl	NH_3_^+^ Cl^−^	H	3.12	0%	0%	-	10%	2%	-
8	**11b**	2-chlorobenzyl	NH_3_^+^ Cl^−^	H	3.62	58%	30%	55 µM	50%	11%	62 µM
9	**13**	2-chlorobenzyl	MeNH_2_^+^ Cl^−^	SMe	4.34	9%	0%	-	38%	14%	-
10	**14**	2-chlorobenzyl	Me_2_NH^+^ Cl^−^	SMe	4.56	5%	0%	-	6%	0%	-
11	**15**	2-chlorobenzyl	NHSO_2_Me	SMe	3.27	−27%	−10%	-	nd	nd	-
12	**16**	2-chlorobenzyl	NHAc	SMe	4.01	0% ^a^	25% ^a^	-	0%	0%	-
13	**17**	2-chlorobenzyl	EtNH_2_^+^ Cl^−^	SMe	4.56	21%	1%	-	47%	13%	-

^a^ Compound was precipitating due to poor solubility. ^b^ CLogP calculated for the free amine using the MLOGP method [31,37] with SwissADME [38]. A list of calculated physiochemical and pharmokinetic properties can be found in the Supplementary Material.

## Data Availability

Not applicable.

## Supplementary Materials

The following supporting information can be downloaded at: https://www.mdpi.com/article/10.3390/molecules27238451/s1, copies of ^1^H NMR and ^13^C NMR spectra; Table S1: Predicted Physiochemical Properties and Lipophilicity for the Tested Tryptaminesa; Table S2: Predicted Pharmacokinetic Properties for the Tested Tryptaminesa.

## Sample Availability

Samples of the compounds can be obtained from the authors subject to availability.
