# Supplementary material for: N1-Benzyl Tryptamine Pan-SHIP1/2 Inhibitors: Synthesis and Preliminary Biological Evaluation as Anti-Tumor Agents
Source: Molecules. 2022 Dec 2;27(23):8451. doi: 10.3390/molecules27238451 (PMC9738565; doi:10.3390/molecules27238451)
Supplement: Supplementary file 1 [file molecules-27-08451-s001.zip › molecules-1971808-supplementary.pdf]

molecules

Supplementary Materials

N1-Benzyl Tryptamine Pan-SHIP1/2 Inhibitors: Synthesis and Preliminary Biological Evaluation as Anti-Tumor Agents

Sandra Fernandes [1], Shea T. Meyer [2], Jigisha P. Shah [2], Arijit A. Adhikari [2], William G. Kerr [1] and John D. Chisholm [2,*]

[1] Department of Microbiology & Immunology, SUNY Upstate Medical University, Syracuse, NY 13210, USA
[2] Department of Chemistry, Syracuse University, Syracuse, NY 13244, USA
* Correspondence: jdchisho@syr.edu

Contents

Table of Contents	S1
NMR Spectra	S2-S24
Tables of Predicted Pharmacodynamic data	S25

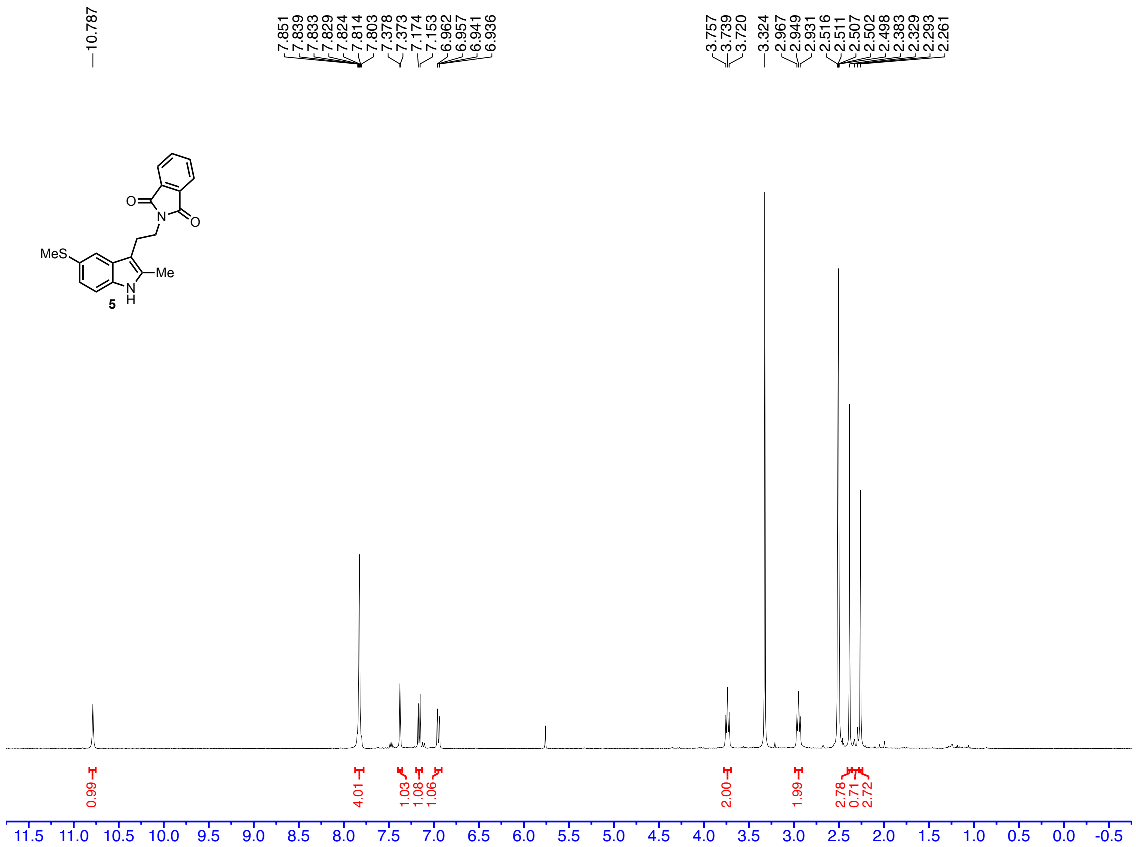

5

STM-2-117 - 1H NMR - 400.13 MHz - DMSO

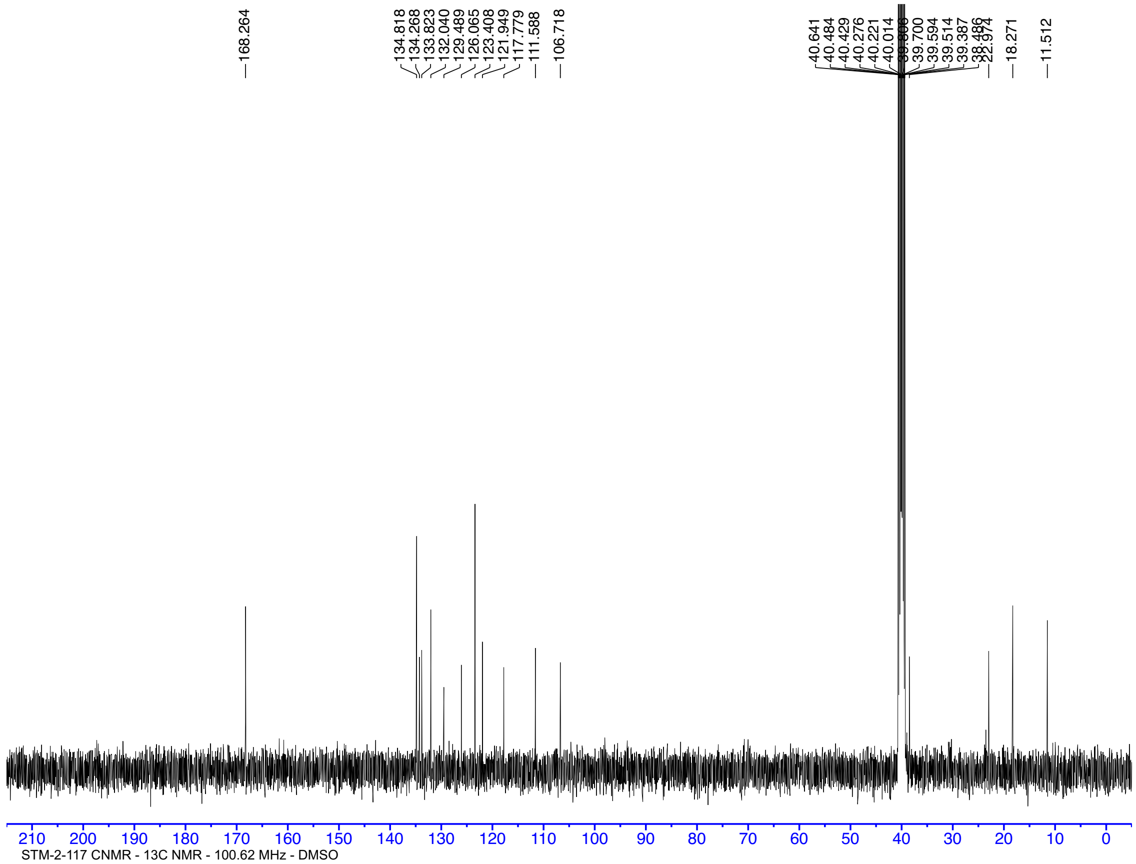

STM-2-117 CNMR - 13C NMR - 100.62 MHz - DMSO

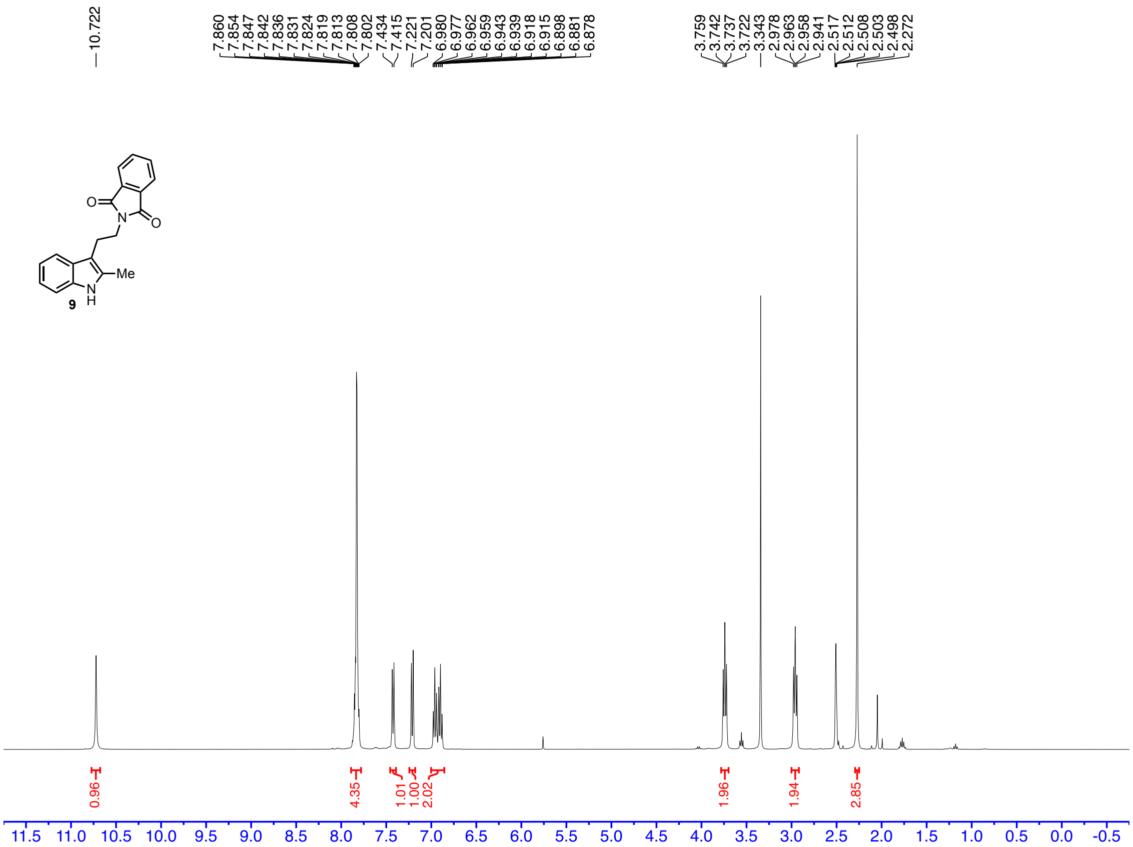

STM-3-2 (8-26-22) - 1H NMR - 400.13 MHz - DMSO

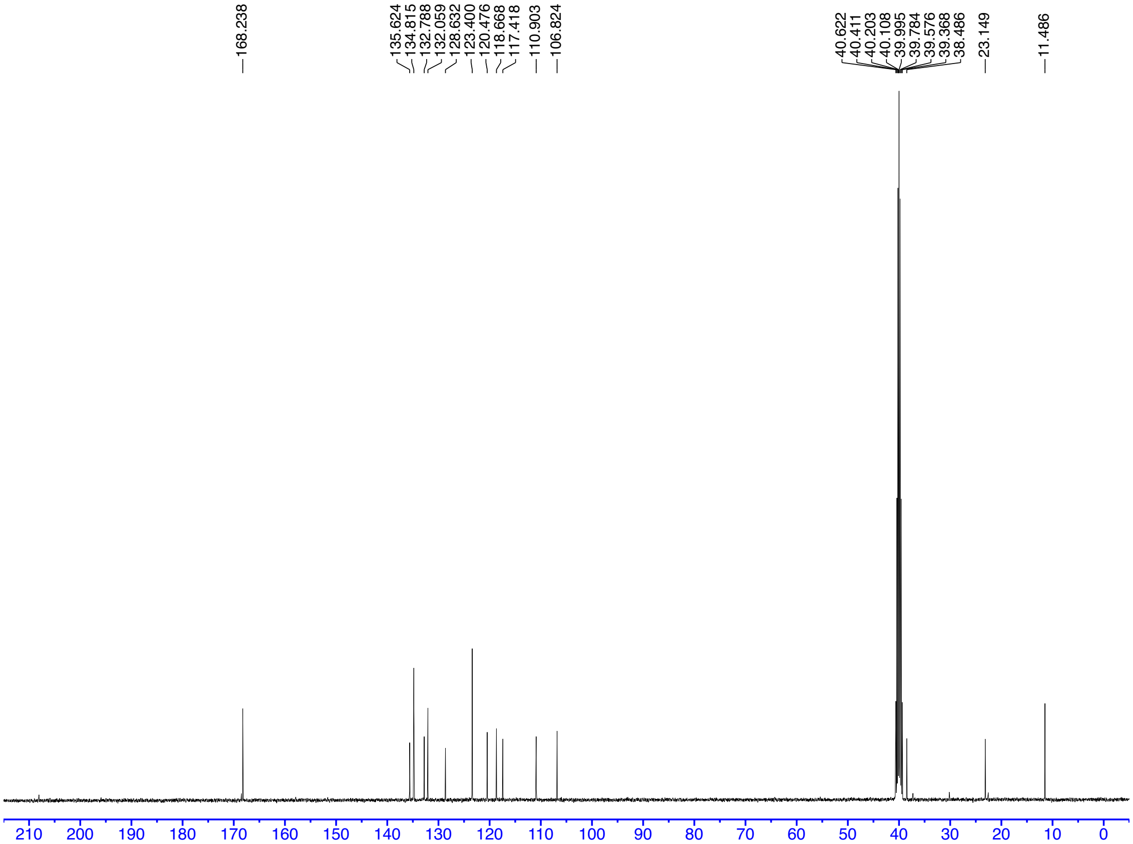

STM-3-2 CNMR (8-26-22) - 13C NMR - 100.62 MHz - DMSO

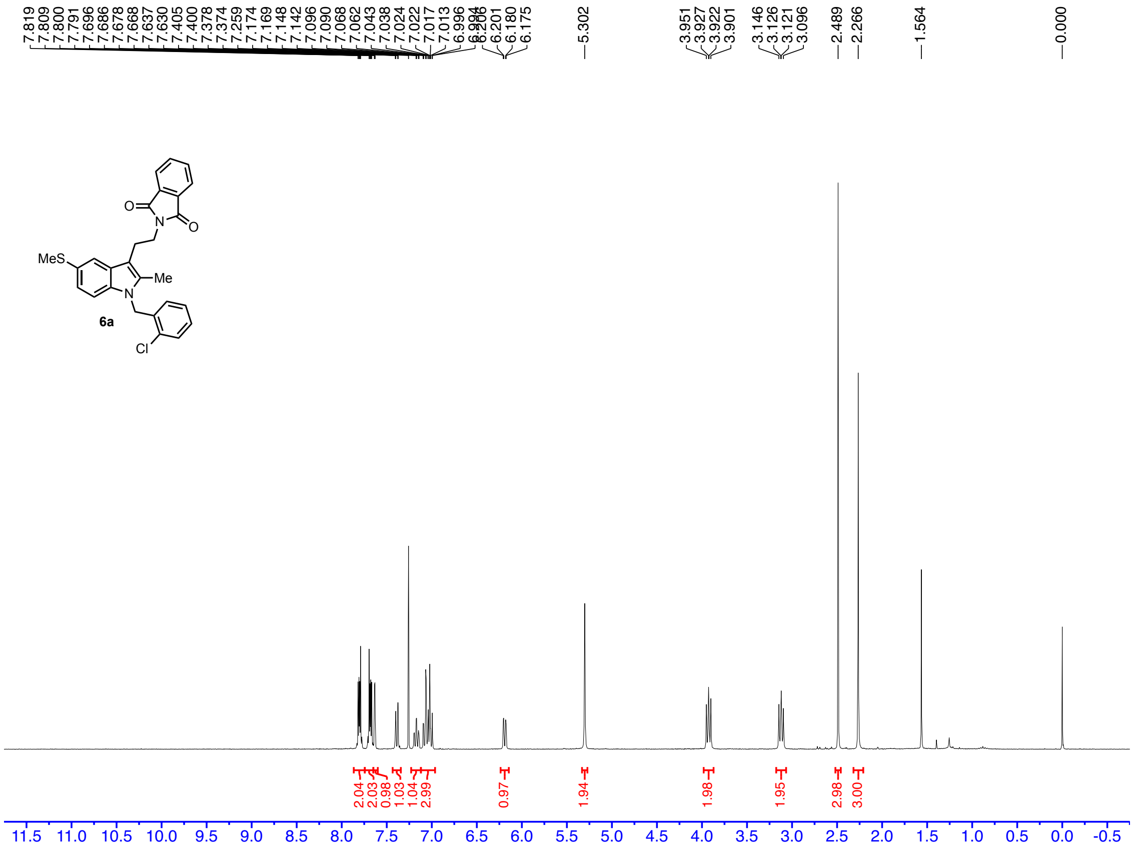

AAA-11-36.1.300 - 1H NMR - 300.20 MHz - CDCl3

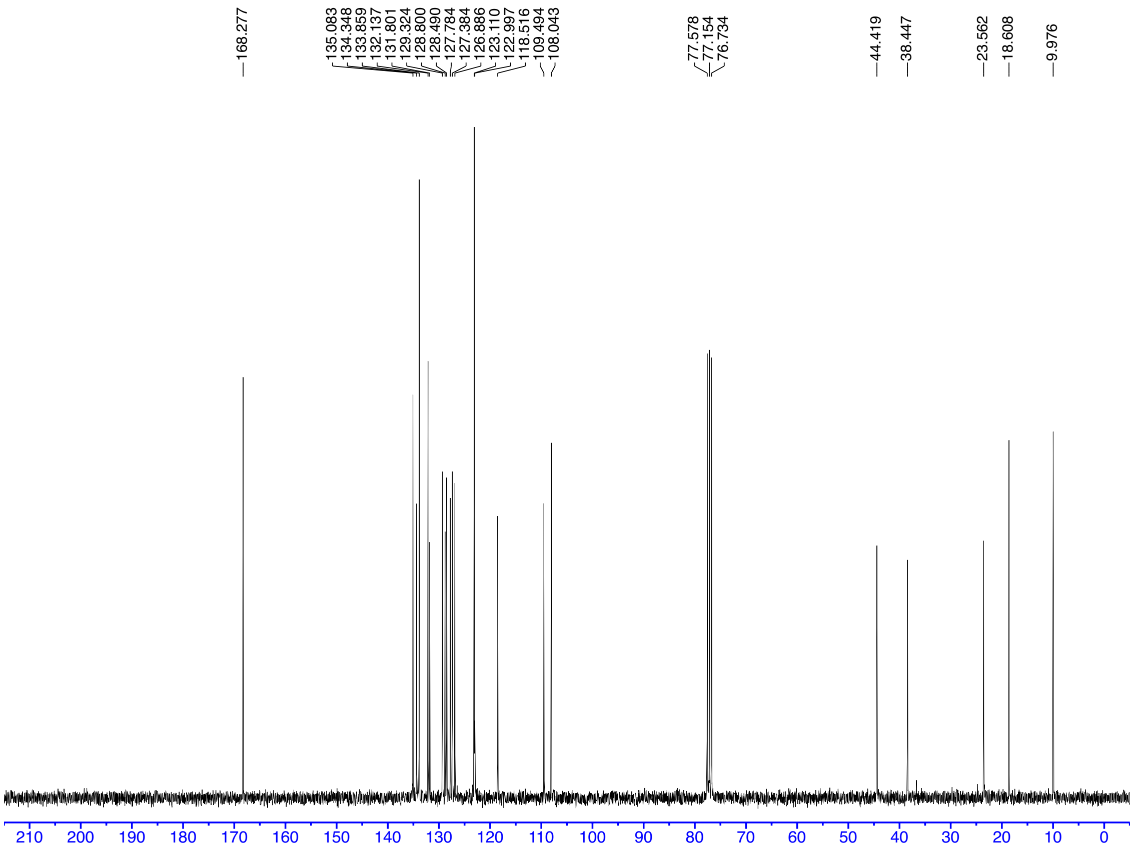

AAA-11-36.13C - 13C NMR - 75.49 MHz - CDCl3

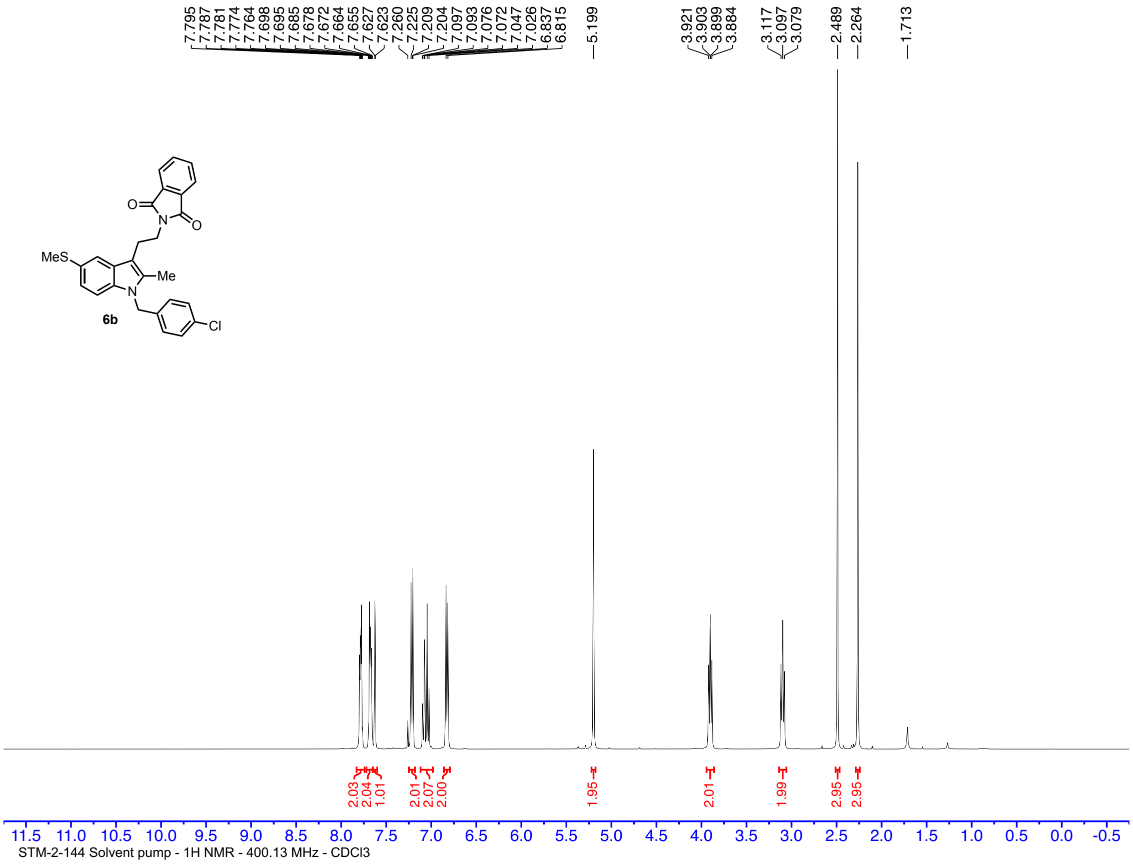

7.795
7.787
7.781
7.774
7.764
7.698
7.695
7.685
7.678
7.672
7.664
7.655
7.627
7.623
7.260
7.225
7.209
7.204
7.097
7.093
7.076
7.072
7.047
7.026
6.837
6.815
5.199
3.921
3.903
3.899
3.884
3.117
3.097
3.079
2.489
2.264
1.713
MeS
Me
N
O
O
N
Cl
6b
2.03
2.04
1.01
2.01
2.07
2.00
1.95
2.01
1.99
2.95
2.95
11.5 11.0 10.5 10.0 9.5 9.0 8.5 8.0 7.5 7.0 6.5 6.0 5.5 5.0 4.5 4.0 3.5 3.0 2.5 2.0 1.5 1.0 0.5 0.0 -0.5
STM-2-144 Solvent pump - 1H NMR - 400.13 MHz - CDCl3

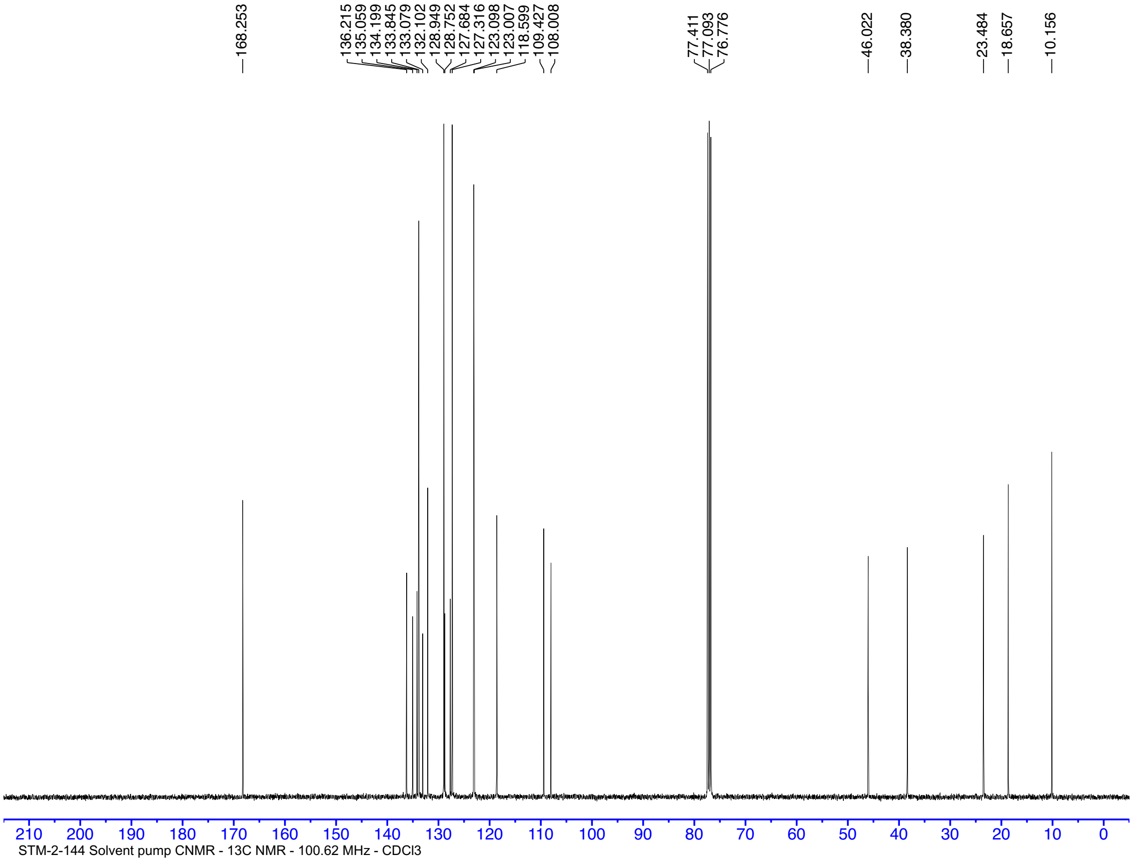

168.253
136.215
135.059
134.199
133.845
133.079
132.102
128.949
128.752
127.684
127.316
123.098
123.007
118.599
109.427
108.008
77.411
77.093
76.776
46.022
38.380
23.484
18.657
10.156
210 200 190 180 170 160 150 140 130 120 110 100 90 80 70 60 50 40 30 20 10 0
STM-2-144 Solvent pump CNMR - 13C NMR - 100.62 MHz - CDCl3

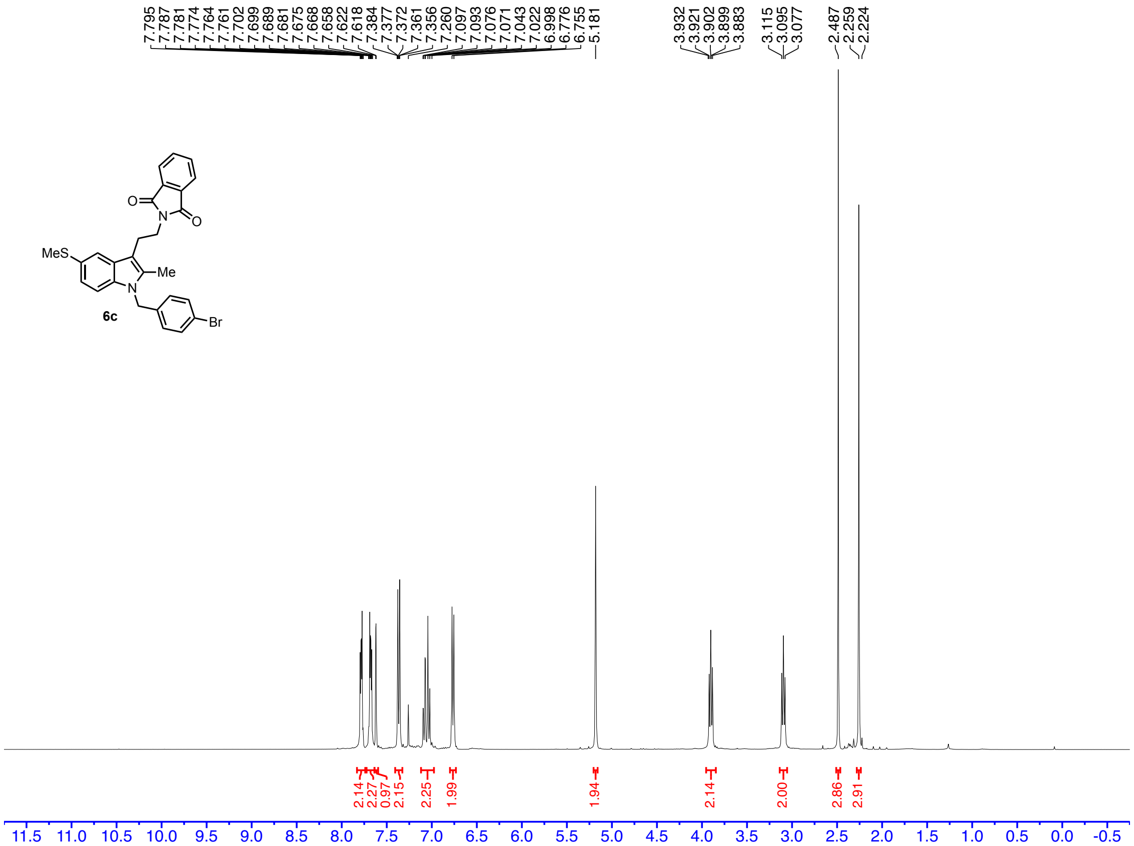

STM-3-36 Solvent Removal CNMR - 1H NMR - 400.13 MHz - CDCl3

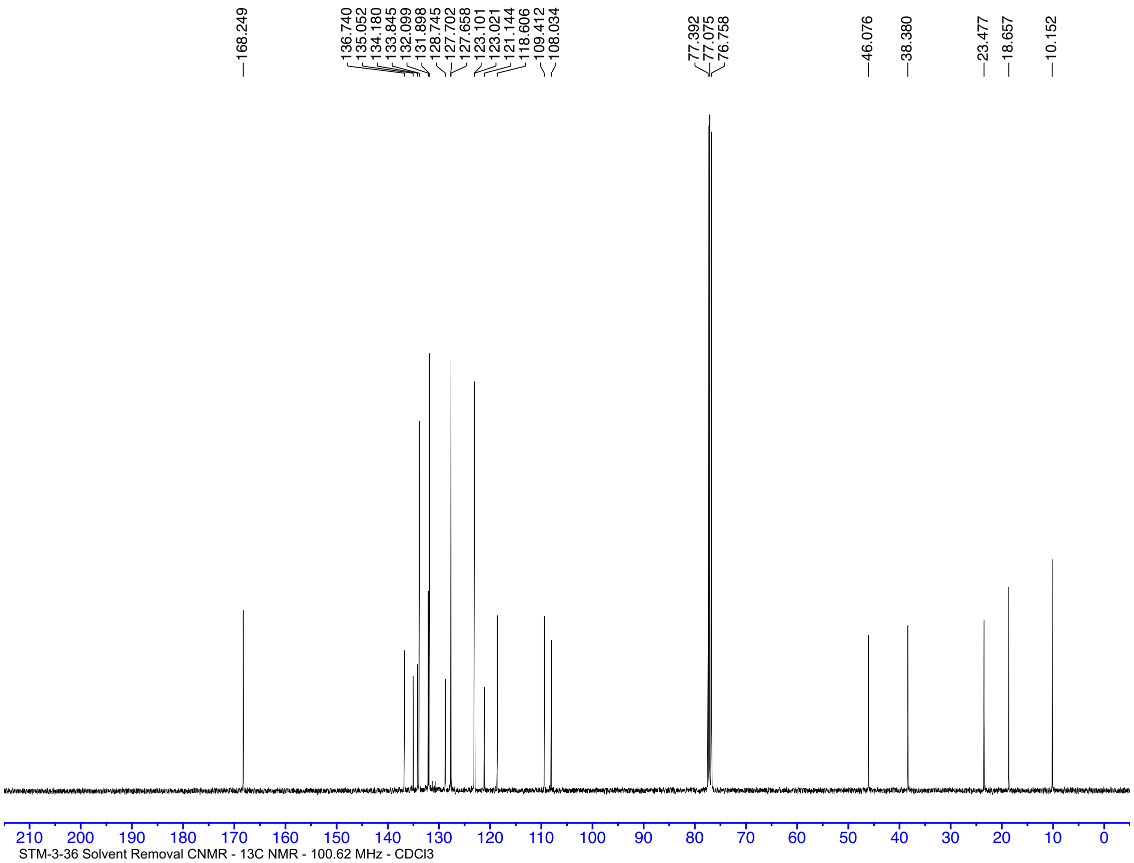

STM-3-36 Solvent Removal CNMR - 13C NMR - 100.62 MHz - CDCl3

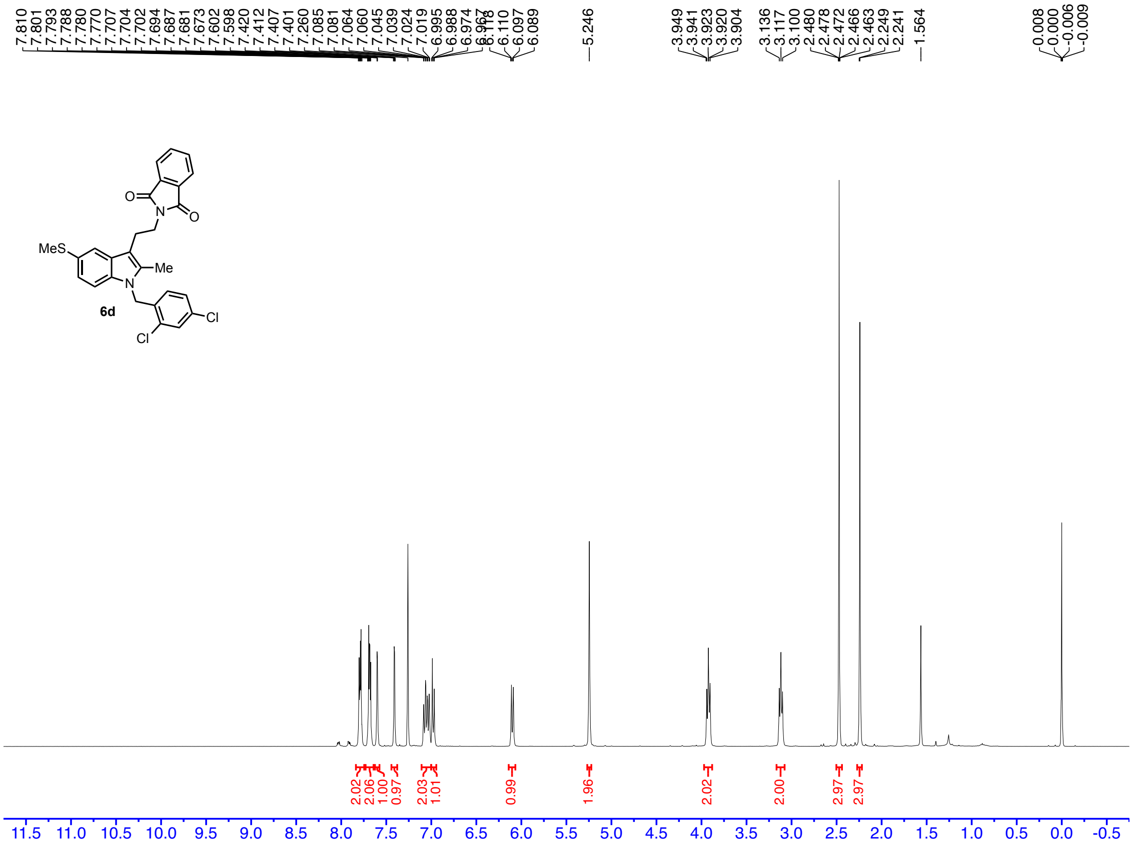

AAA-11-35.1H - 1H NMR - 400.13 MHz - CDCl3

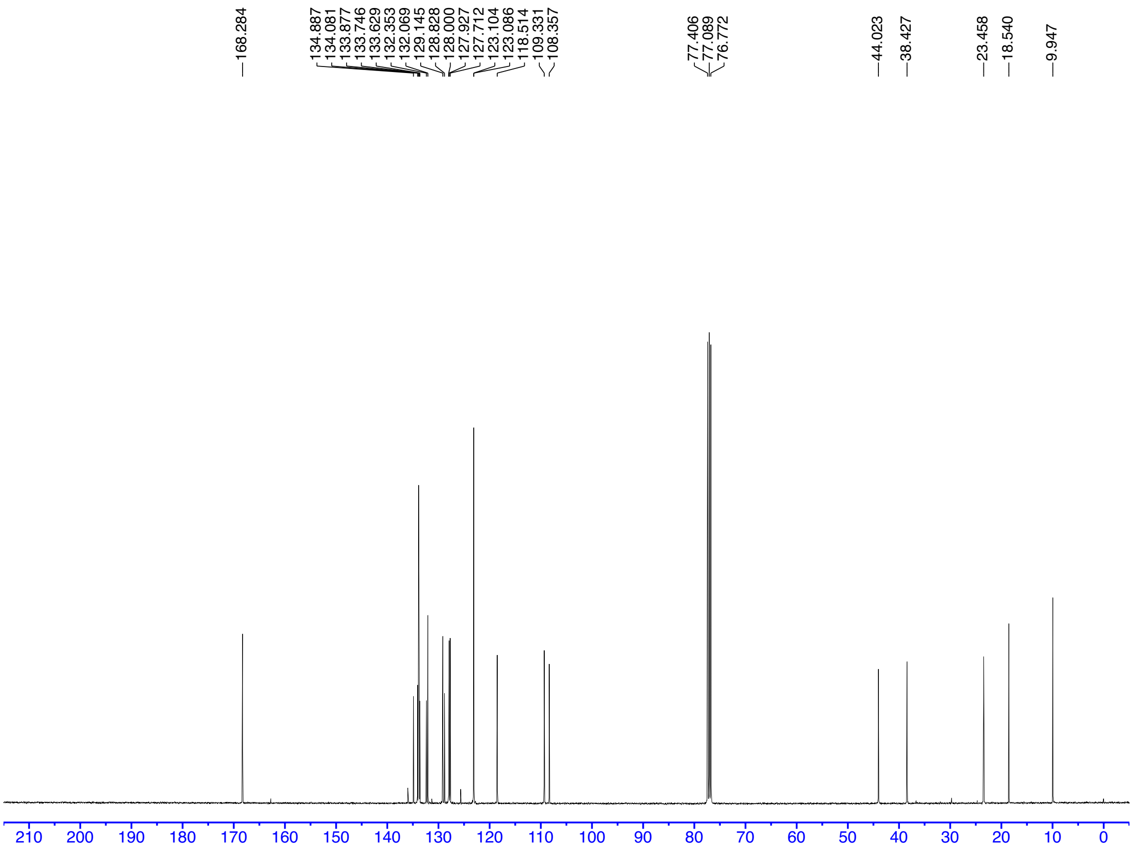

AAA-11-35.13C - 13C NMR - 100.62 MHz - CDCl3

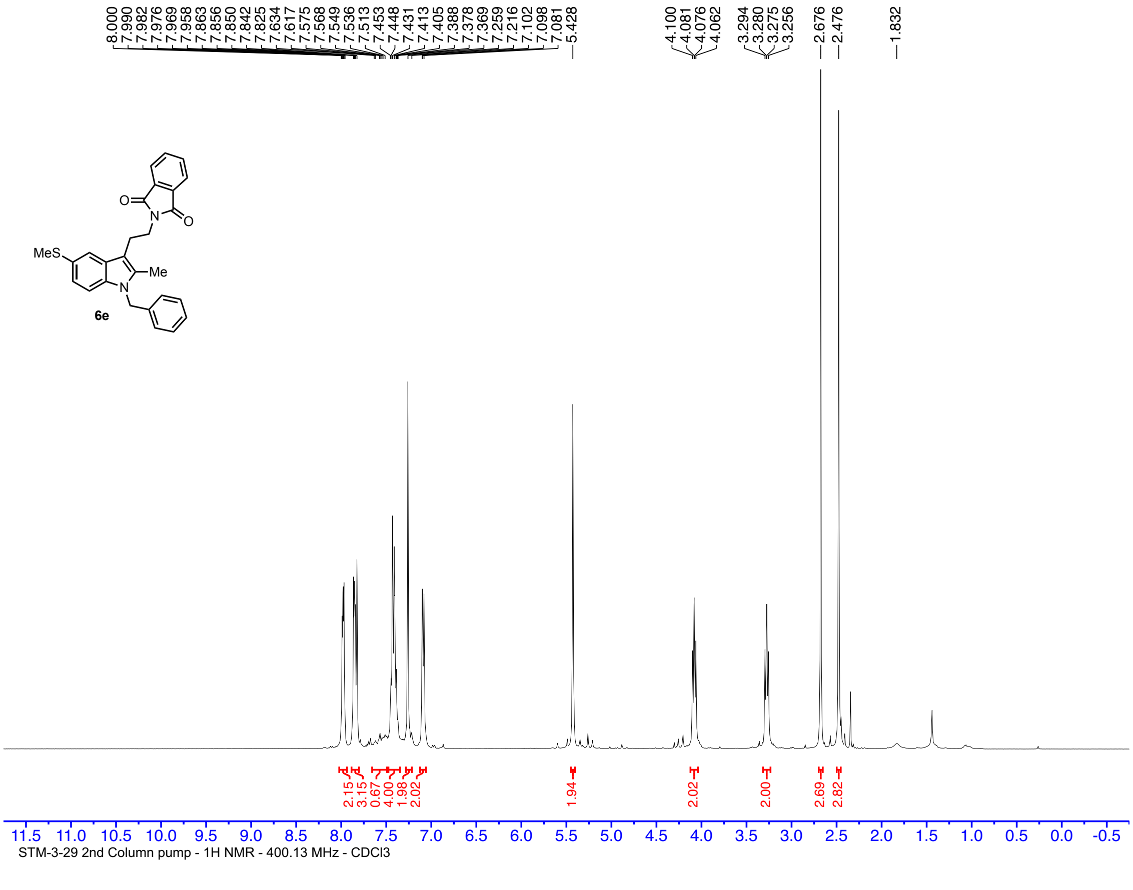
8.000
7.990
7.982
7.976
7.969
7.958
7.863
7.856
7.850
7.842
7.825
7.634
7.617
7.575
7.568
7.549
7.536
7.513
7.453
7.448
7.431
7.413
7.405
7.388
7.378
7.369
7.259
7.216
7.102
7.098
7.081
5.428
4.100
4.081
4.076
4.062
3.294
3.280
3.275
3.256
2.676
2.476
1.832
MeS
N
O
O
Me
6e
2.15
3.15
0.67
4.00
1.98
2.02
1.94
2.02
2.00
2.69
2.82
11.5 11.0 10.5 10.0 9.5 9.0 8.5 8.0 7.5 7.0 6.5 6.0 5.5 5.0 4.5 4.0 3.5 3.0 2.5 2.0 1.5 1.0 0.5 0.0 -0.5
STM-3-29 2nd Column pump - 1H NMR - 400.13 MHz - CDCl3

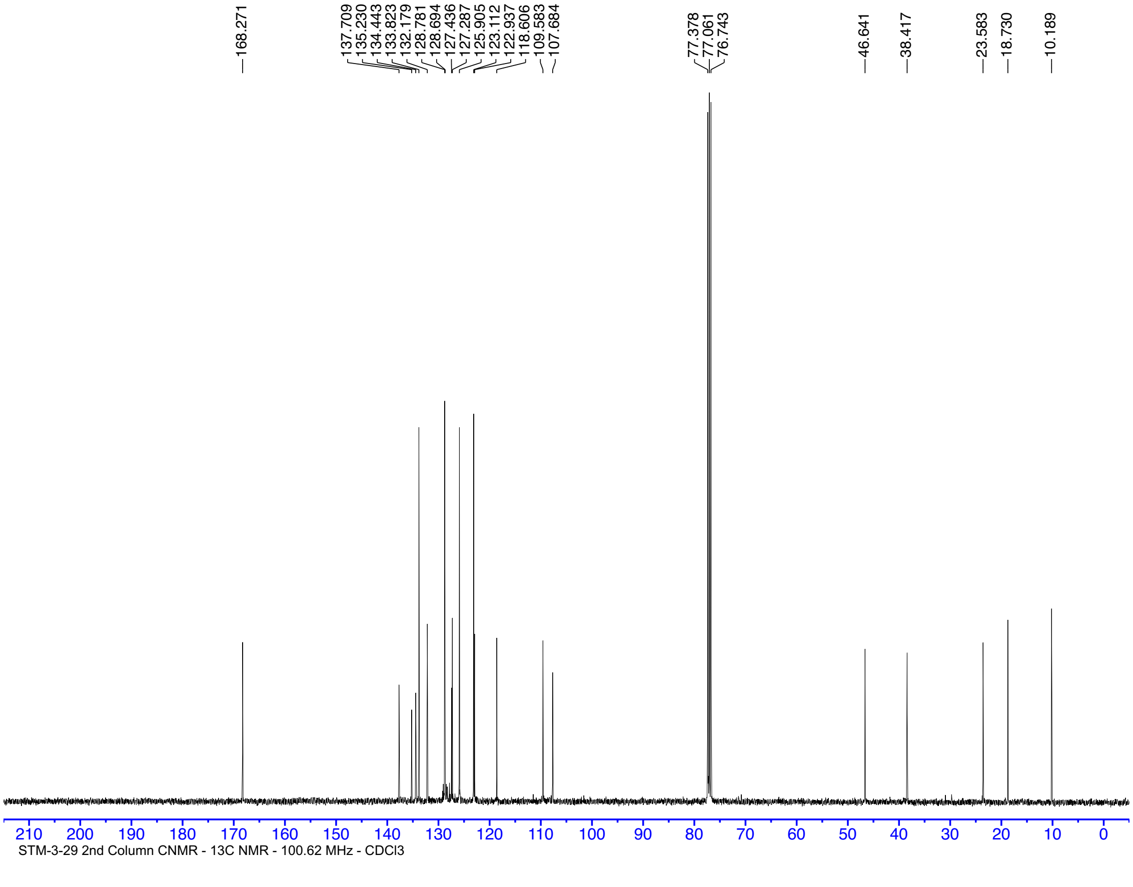
168.271
137.709
135.230
134.443
133.823
132.179
128.781
128.694
127.436
127.287
125.905
123.112
122.937
118.606
109.583
107.684
77.378
77.061
76.743
46.641
38.417
23.583
18.730
10.189
210 200 190 180 170 160 150 140 130 120 110 100 90 80 70 60 50 40 30 20 10 0
STM-3-29 2nd Column CNMR - 13C NMR - 100.62 MHz - CDCl3

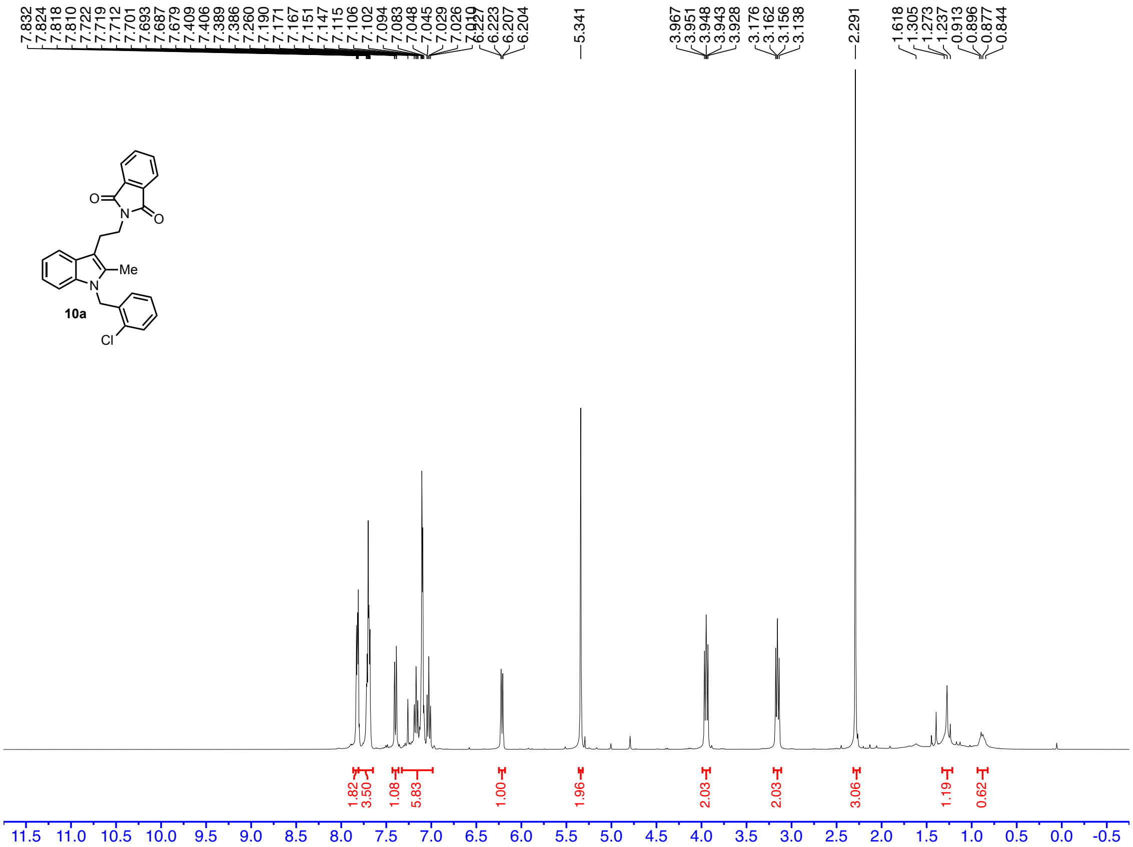

STM-3-17 - 1H NMR - 400.13 MHz - CDCl3

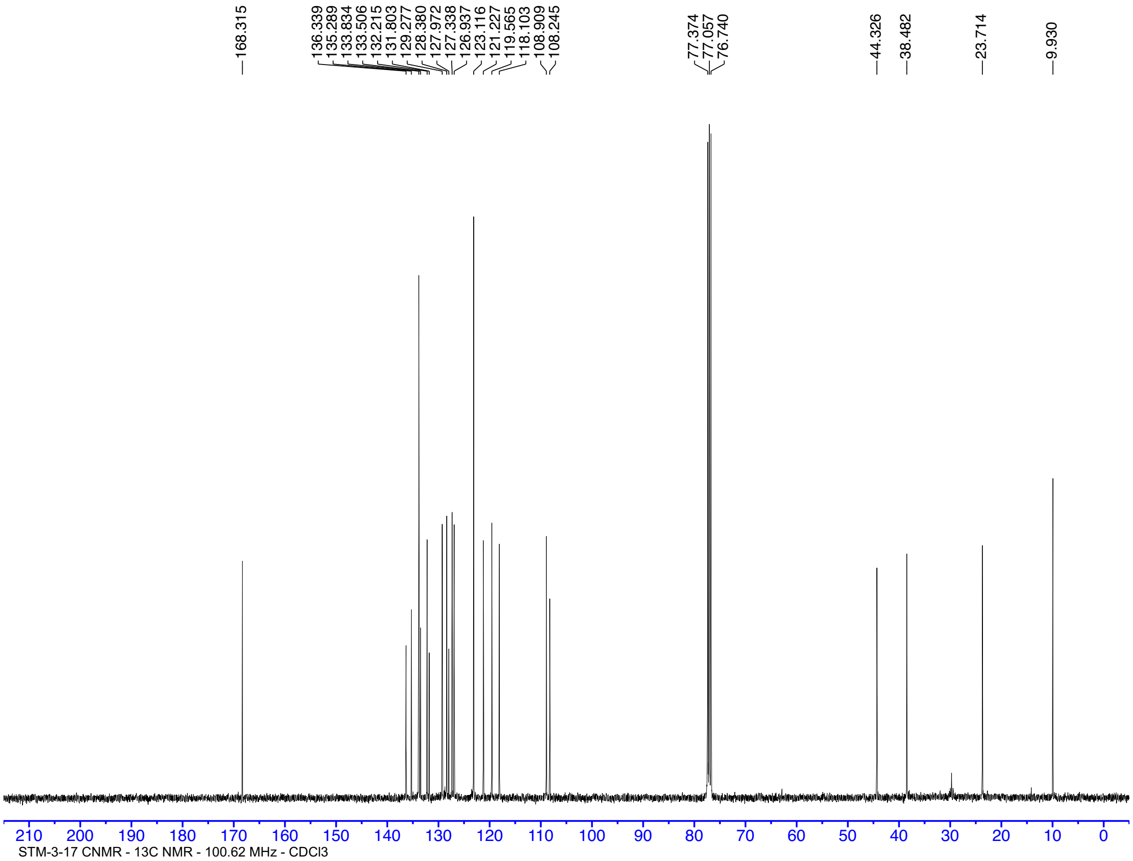

STM-3-17 CNMR - 13C NMR - 100.62 MHz - CDCl3

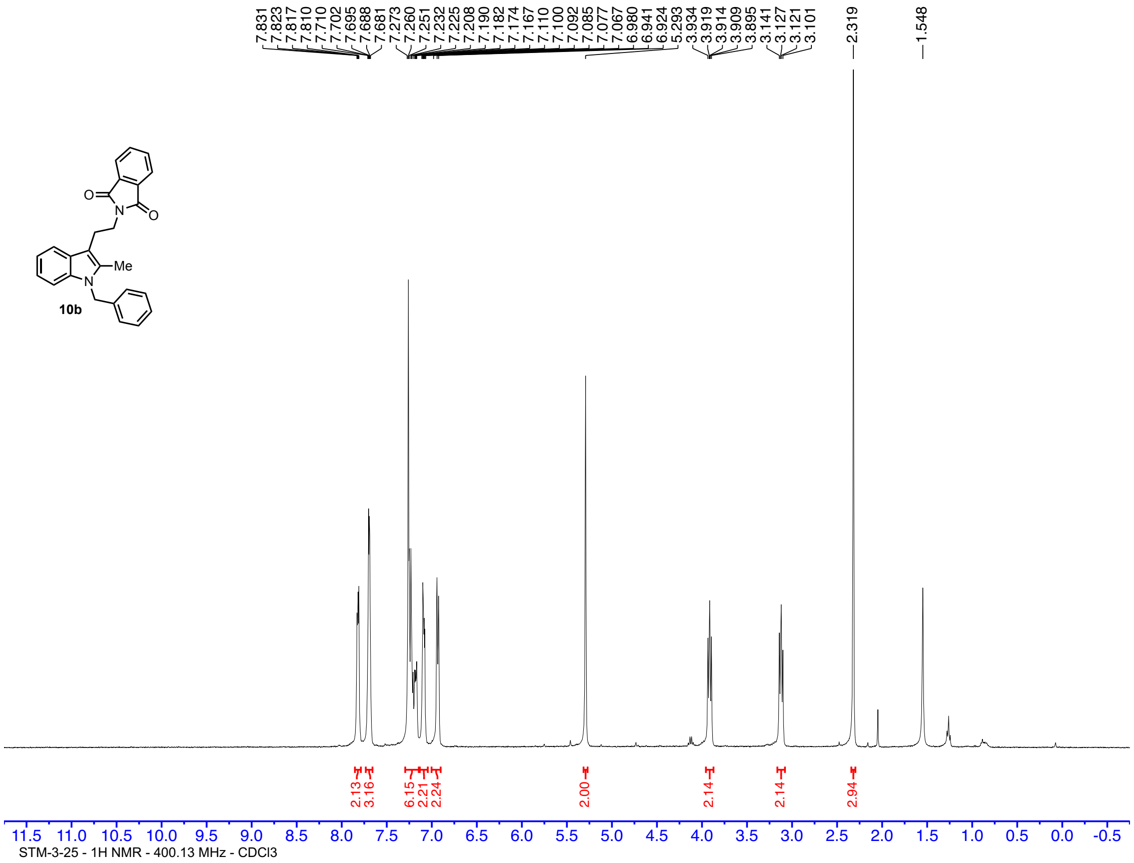
O
N
O
Me
N
10b
7.831
7.823
7.817
7.810
7.702
7.695
7.688
7.681
7.273
7.260
7.251
7.232
7.225
7.208
7.190
7.182
7.174
7.167
7.110
7.100
7.092
7.085
7.077
7.067
6.980
6.941
6.924
5.293
3.934
3.919
3.914
3.909
3.895
3.141
3.127
3.121
3.101
2.319
1.548
2.13
3.16
6.15
2.21
2.24
2.00
2.14
2.14
2.94
11.5 11.0 10.5 10.0 9.5 9.0 8.5 8.0 7.5 7.0 6.5 6.0 5.5 5.0 4.5 4.0 3.5 3.0 2.5 2.0 1.5 1.0 0.5 0.0 -0.5
STM-3-25 - 1H NMR - 400.13 MHz - CDCl3

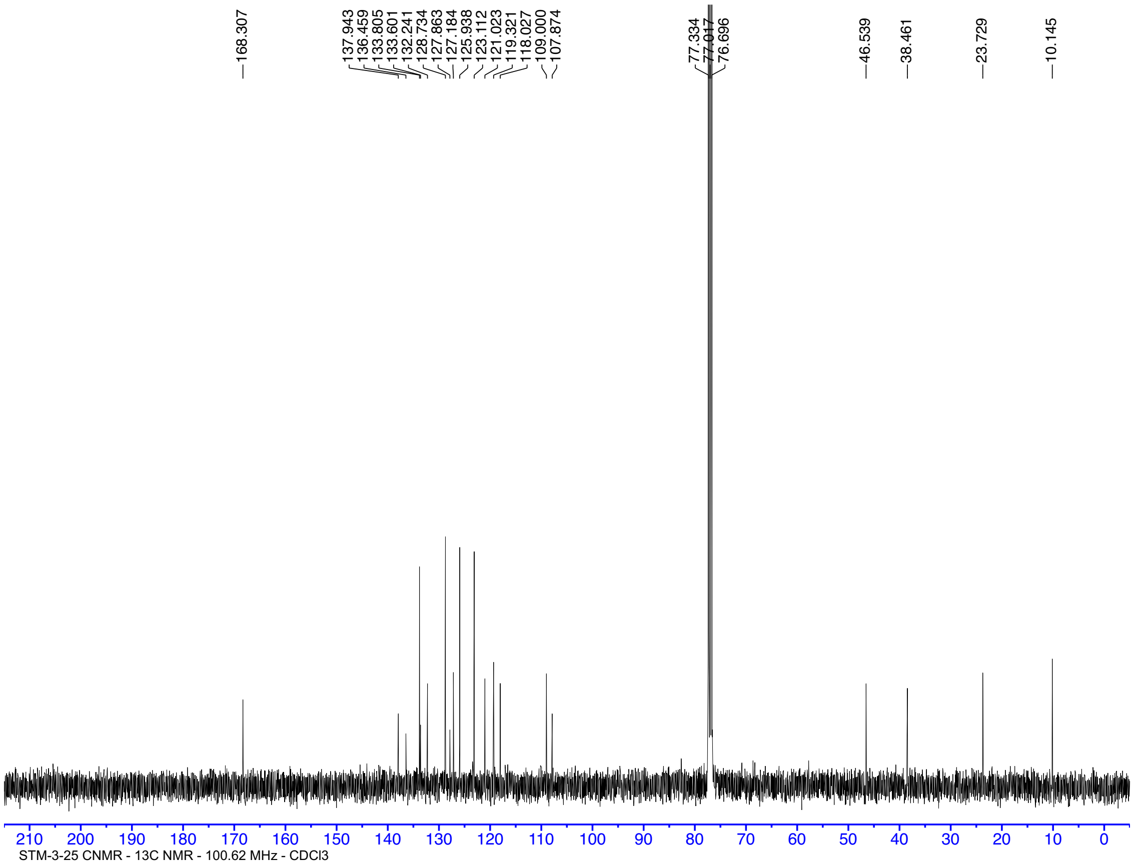
168.307
137.943
136.459
133.805
133.601
132.241
128.734
127.863
127.184
125.938
123.112
121.023
119.321
118.027
109.000
107.874
77.334
77.017
76.696
46.539
38.461
23.729
10.145
210 200 190 180 170 160 150 140 130 120 110 100 90 80 70 60 50 40 30 20 10 0
STM-3-25 CNMR - 13C NMR - 100.62 MHz - CDCl3

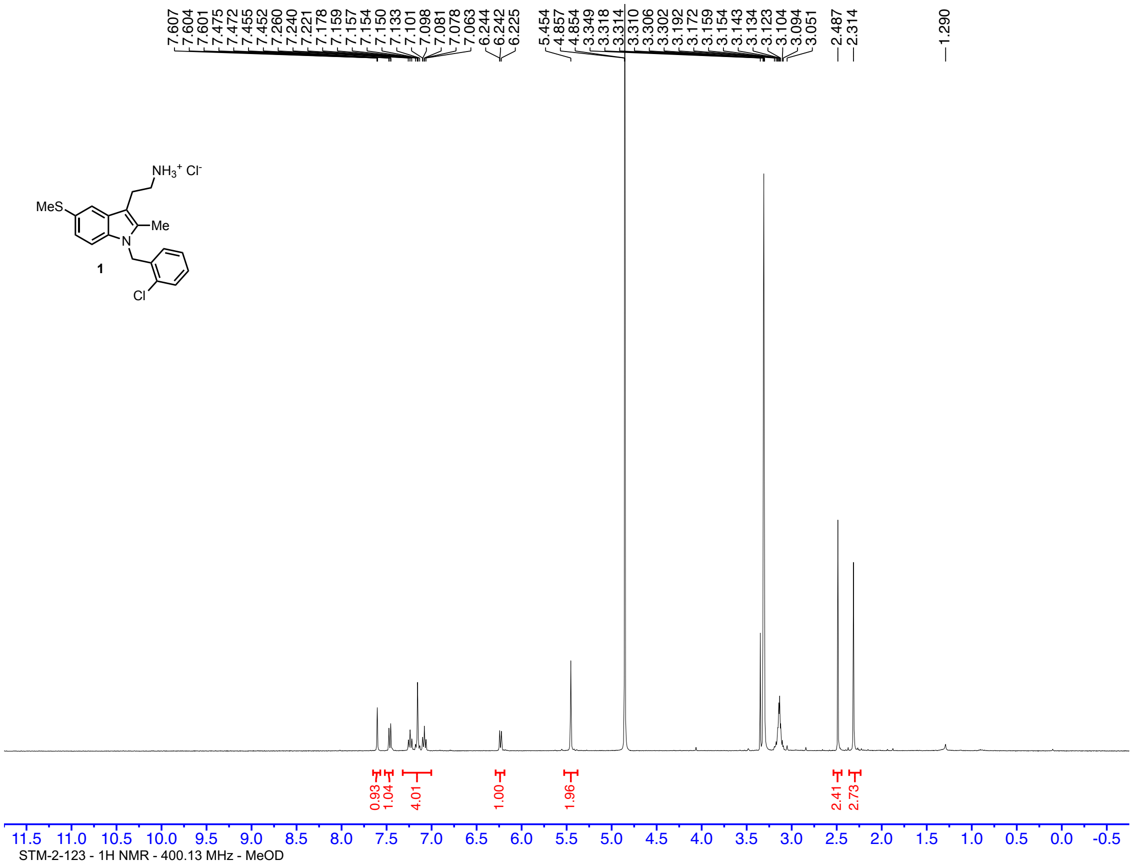

7.607 7.604 7.601 7.475 7.472 7.455 7.452 7.260 7.240 7.221 7.178 7.159 7.157 7.154 7.150 7.133 7.101 7.098 7.081 7.078 7.063 6.244 6.242 6.225 5.454 4.857 4.854 3.349 3.318 3.314 3.310 3.306 3.302 3.192 3.172 3.159 3.154 3.143 3.134 3.123 3.104 3.094 3.051 2.487 2.314 1.290
NH3+ Cl-
MeS
Me
N
Cl
1
0.93 1.04 4.01 1.00 1.96 2.41 2.73
11.5 11.0 10.5 10.0 9.5 9.0 8.5 8.0 7.5 7.0 6.5 6.0 5.5 5.0 4.5 4.0 3.5 3.0 2.5 2.0 1.5 1.0 0.5 0.0 -0.5
STM-2-123 - 1H NMR - 400.13 MHz - MeOD

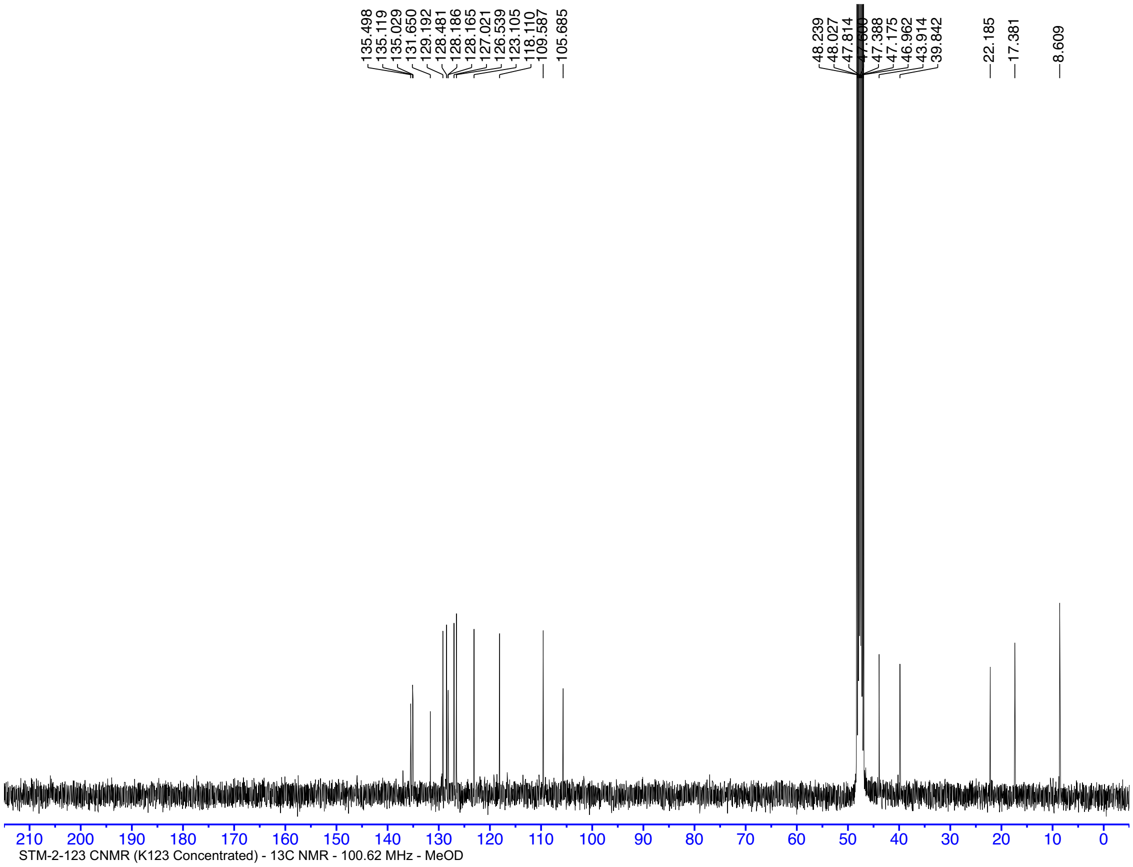

135.498 135.119 135.029 131.650 129.192 128.481 128.186 128.165 127.021 126.539 123.105 118.110 109.587 105.685
48.239 48.027 47.814 47.388 47.175 46.962 43.914 39.842
22.185 17.381 8.609
210 200 190 180 170 160 150 140 130 120 110 100 90 80 70 60 50 40 30 20 10 0
STM-2-123 CNMR (K123 Concentrated) - 13C NMR - 100.62 MHz - MeOD

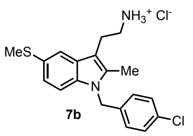

7b

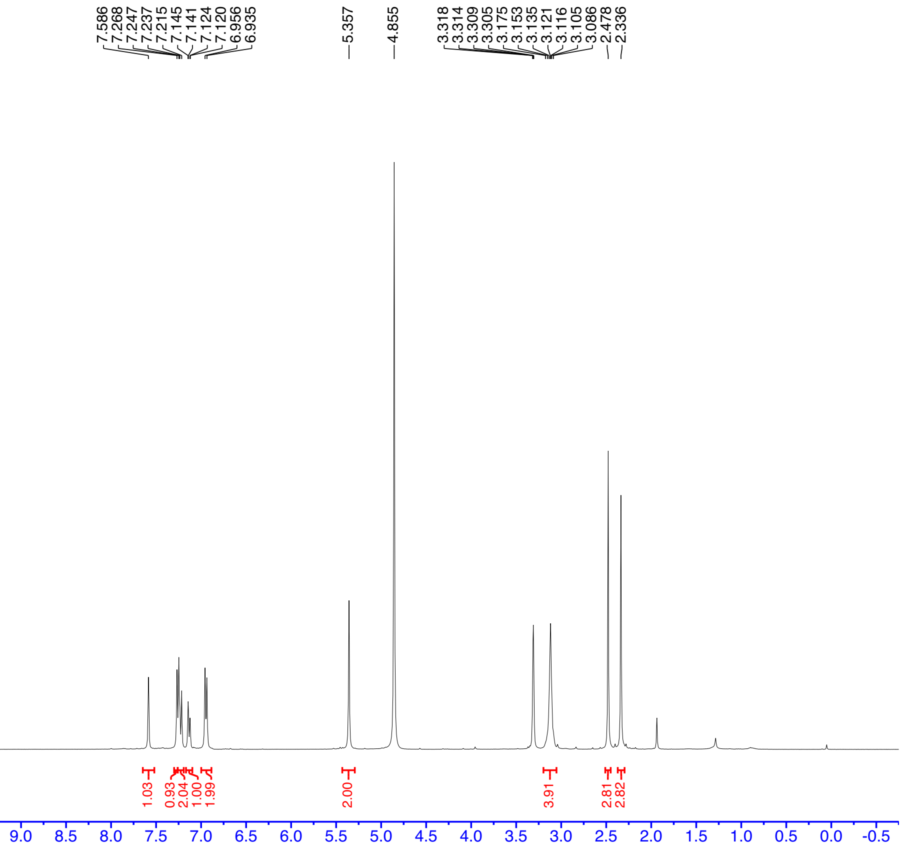

STM-2-150 diluted Second attempt - 1H NMR - 400.13 MHz - MeOD

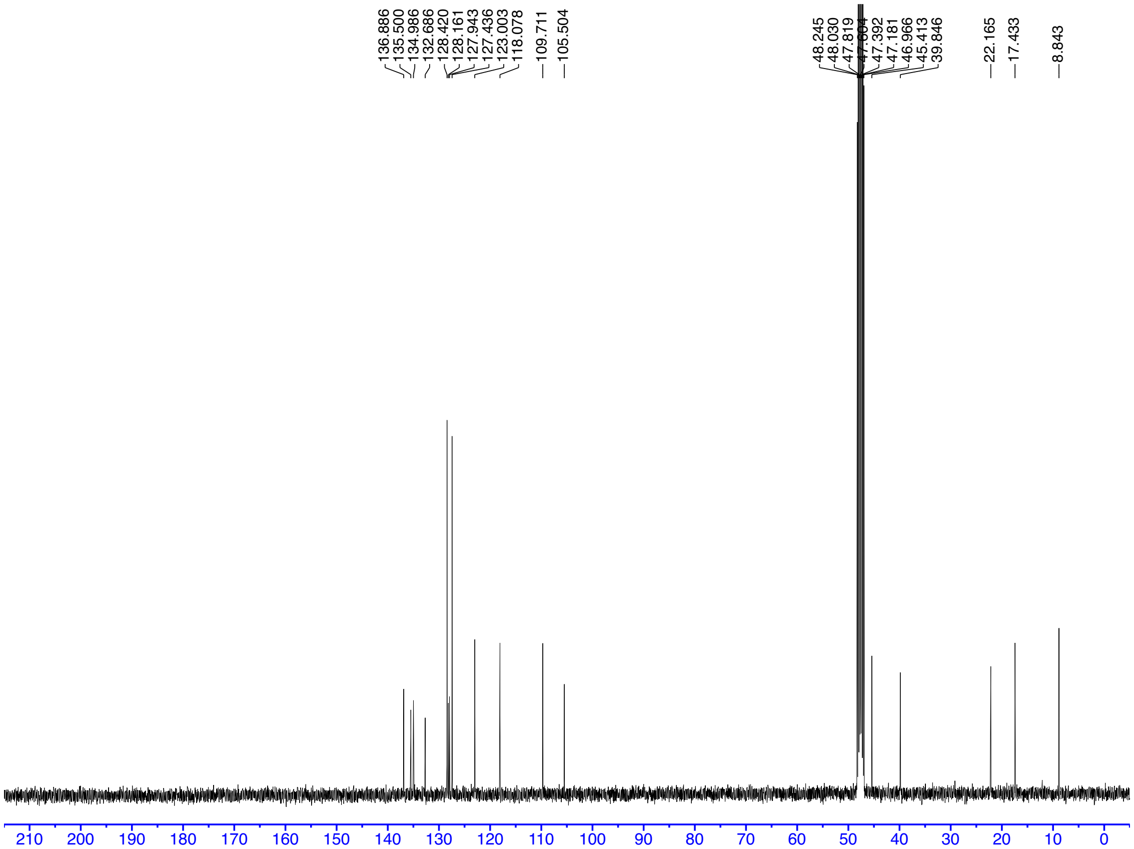

STM-2-150 CNMR - 13C NMR - 100.62 MHz - MeOD

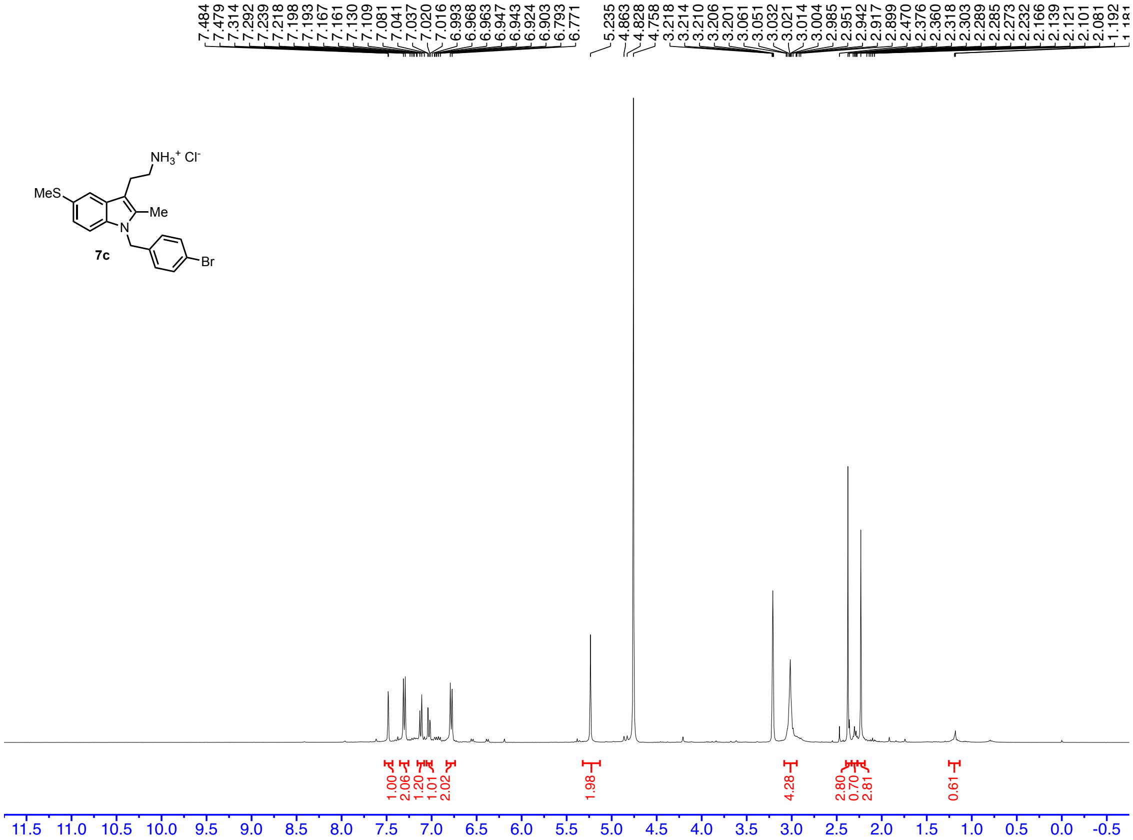

STM-3-75 CNMR - 1H NMR - 400.13 MHz - MeOD

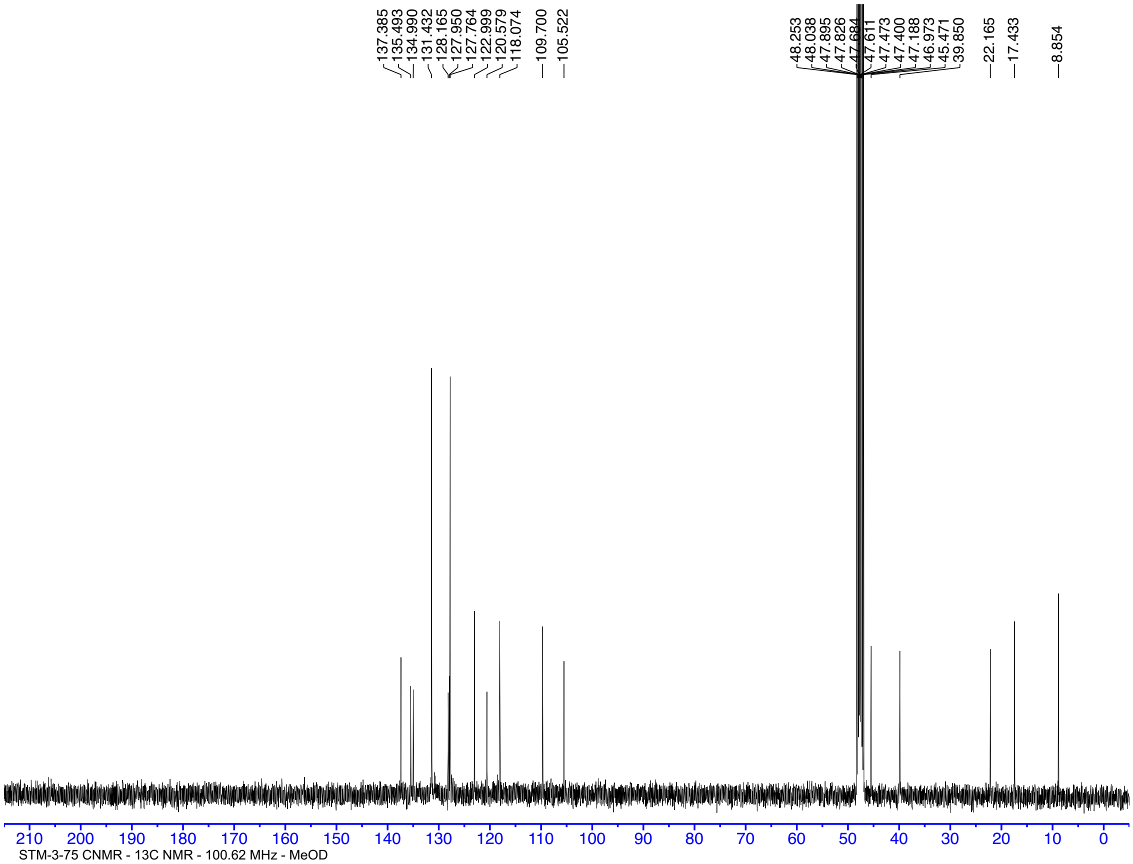

STM-3-75 CNMR - 13C NMR - 100.62 MHz - MeOD

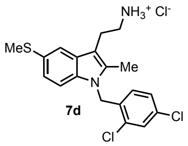

7d

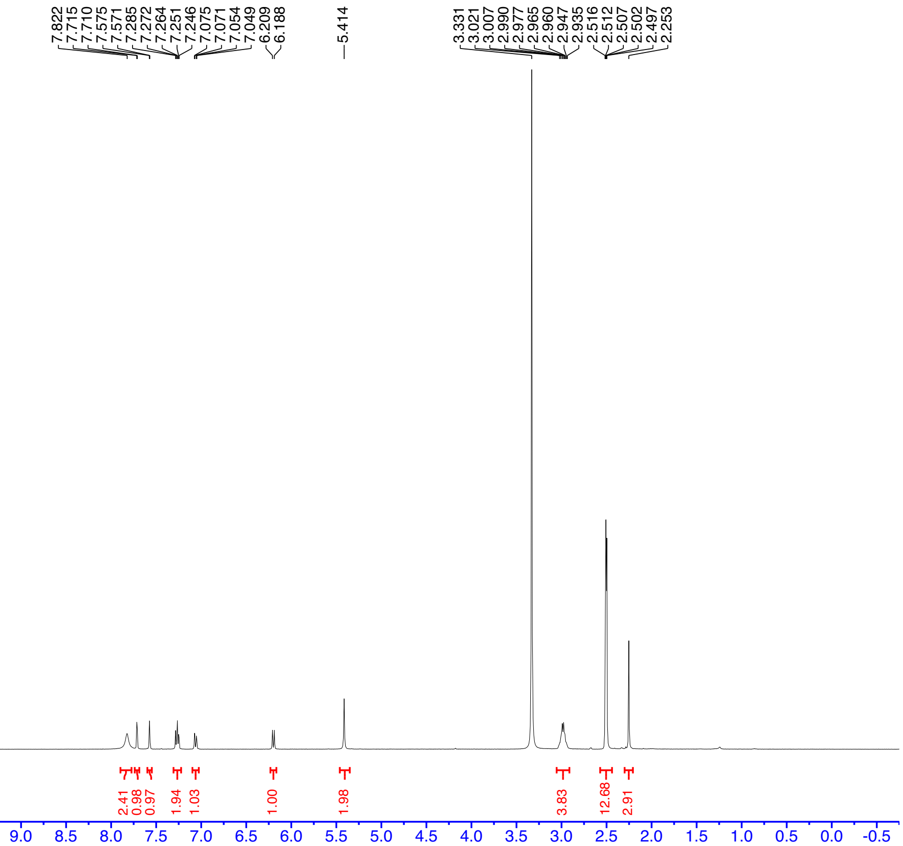

K194 Recrystalized - 1H NMR - 400.13 MHz - DMSO

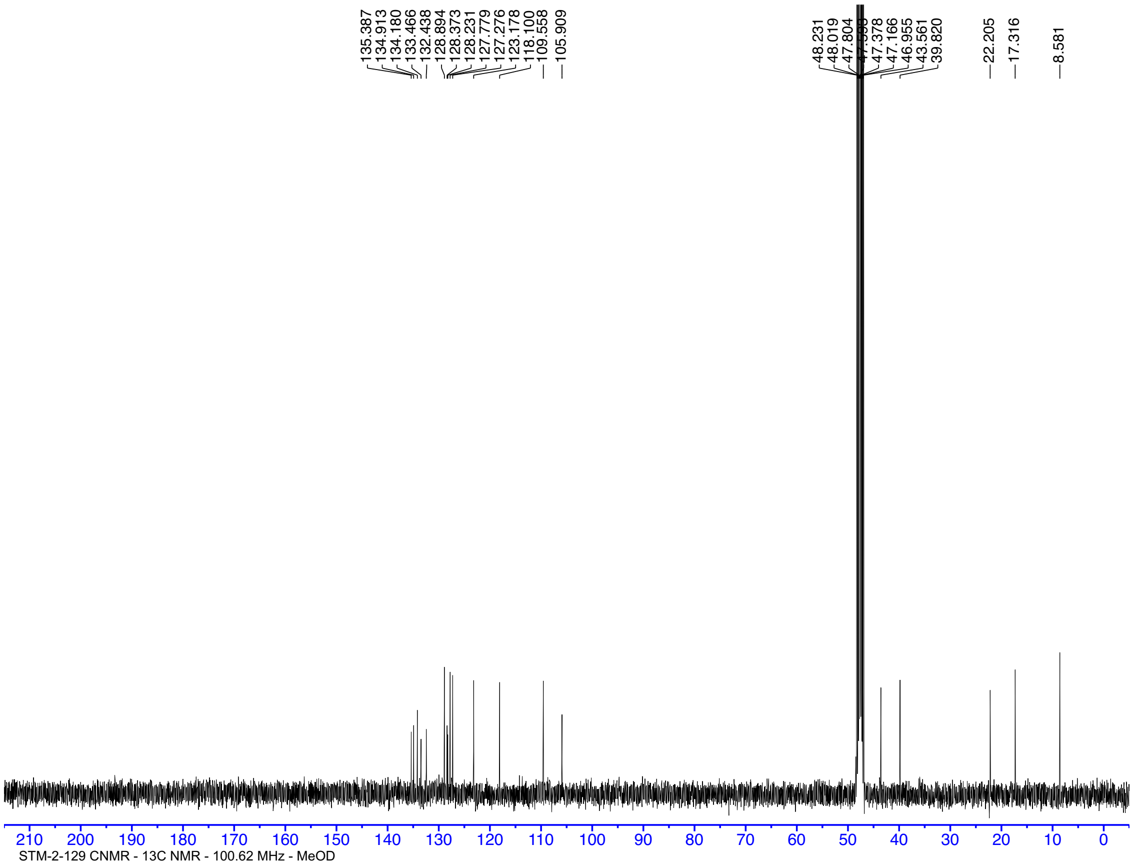

STM-2-129 CNMR - 13C NMR - 100.62 MHz - MeOD

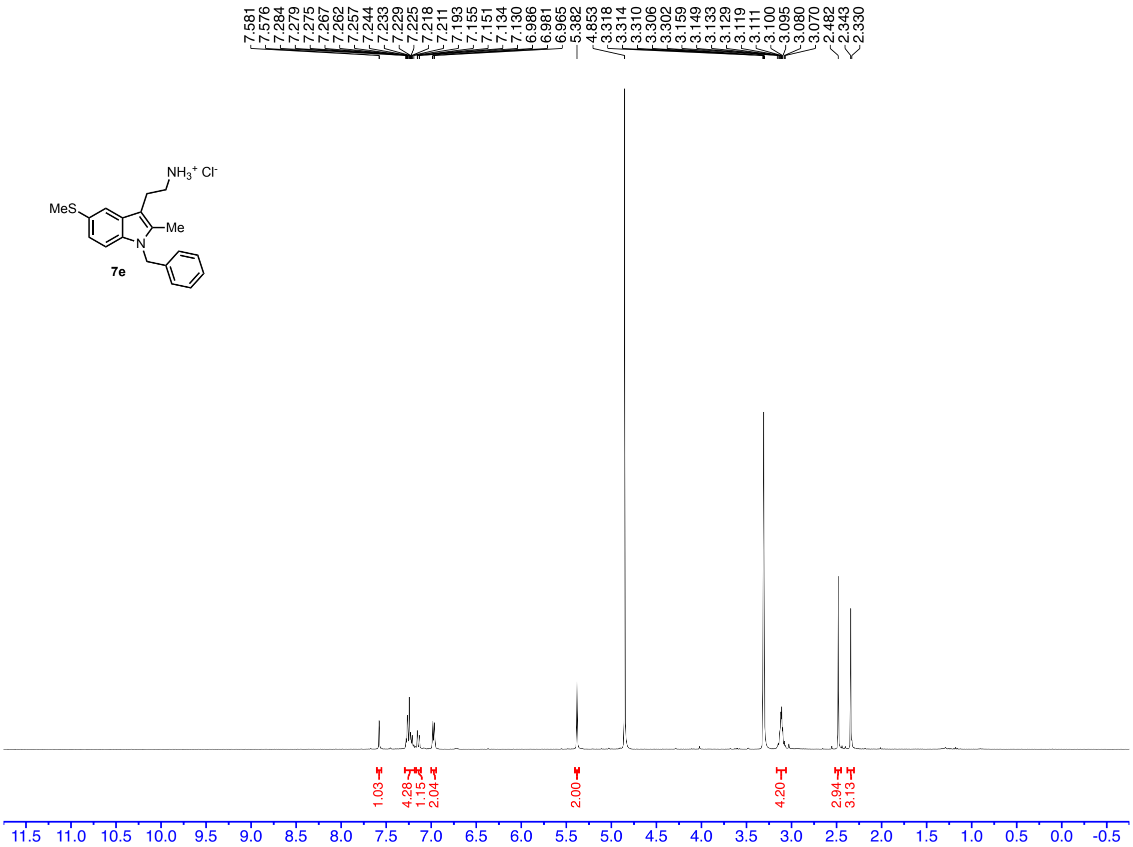

STM-3-64 - 1H NMR - 400.13 MHz - MeOD

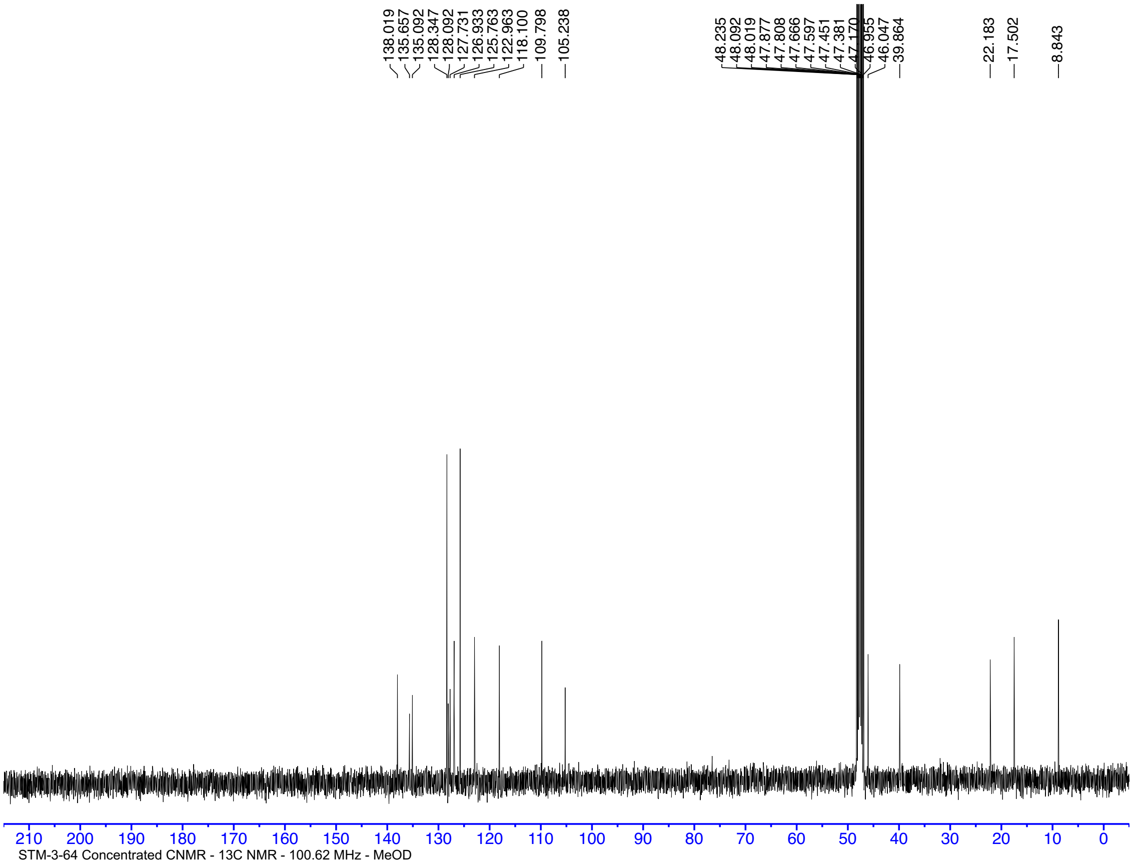

STM-3-64 Concentrated CNMR - 13C NMR - 100.62 MHz - MeOD

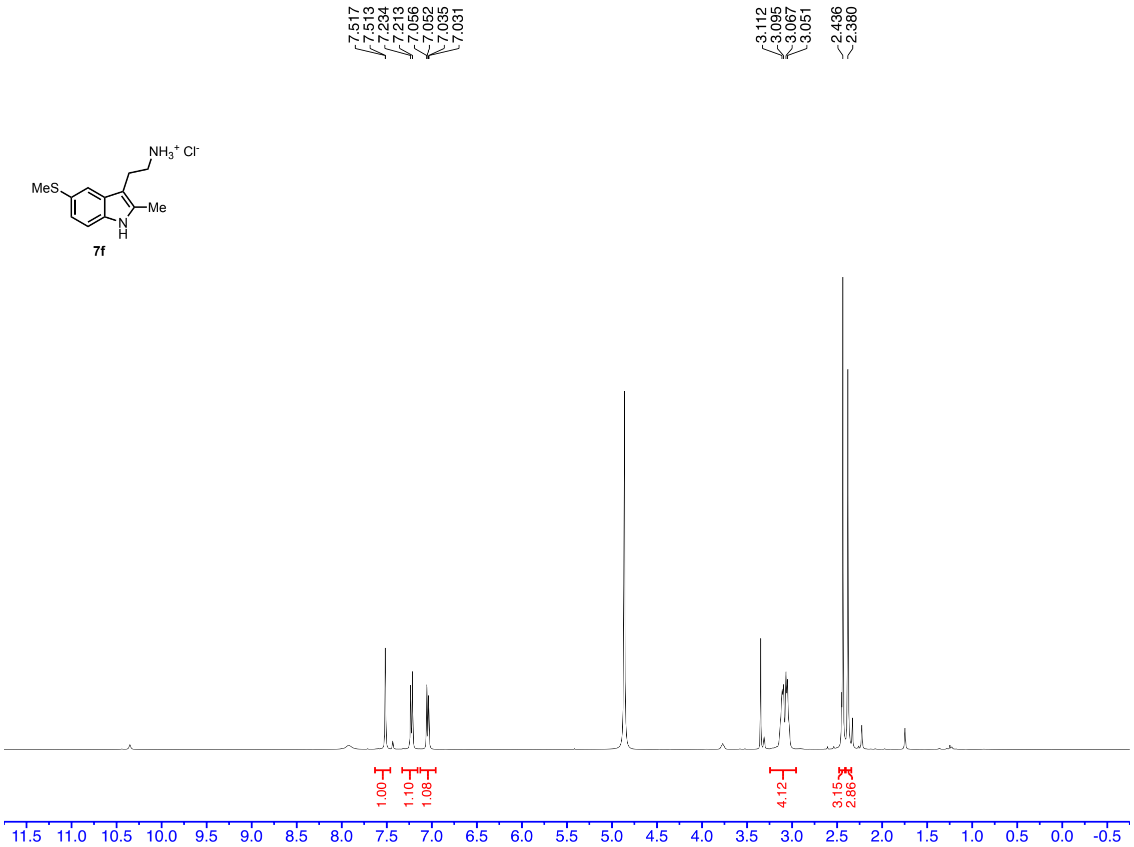

STM-3-16 Ethanol Removal - 1H NMR - 400.13 MHz - MeOD

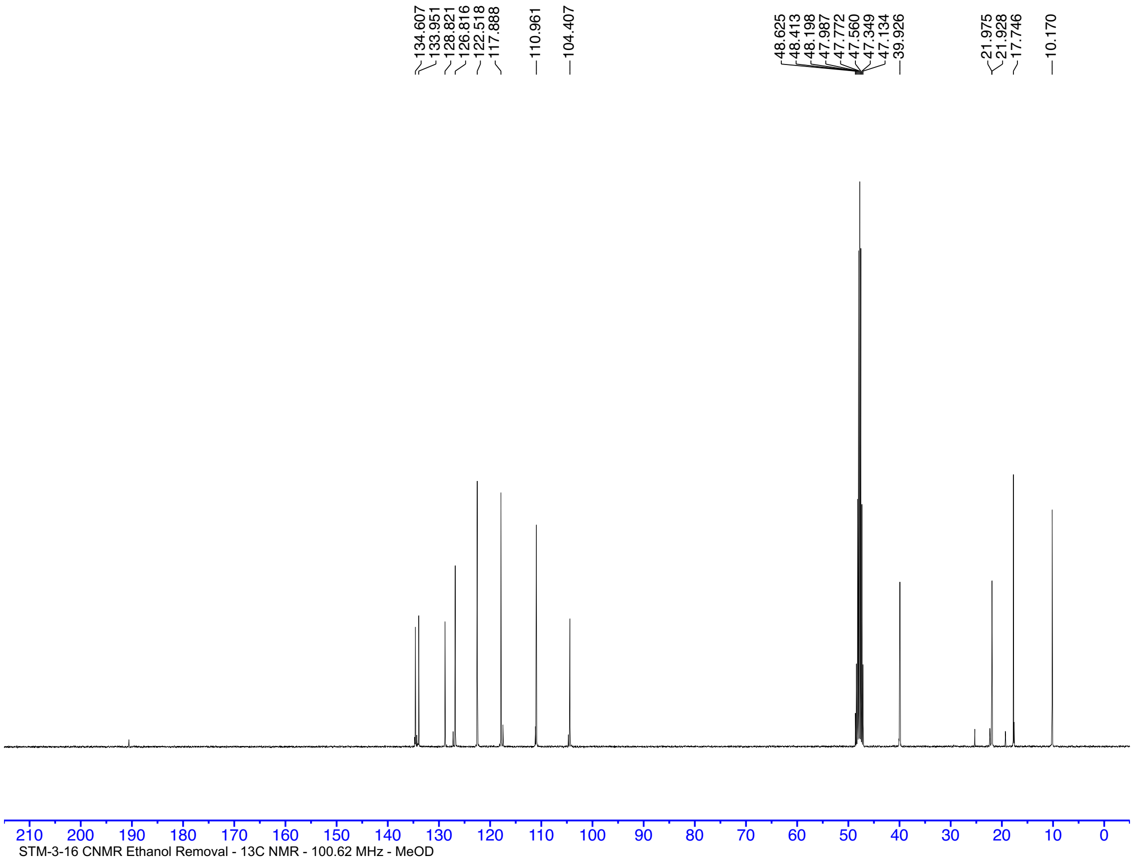

STM-3-16 CNMR Ethanol Removal - 13C NMR - 100.62 MHz - MeOD

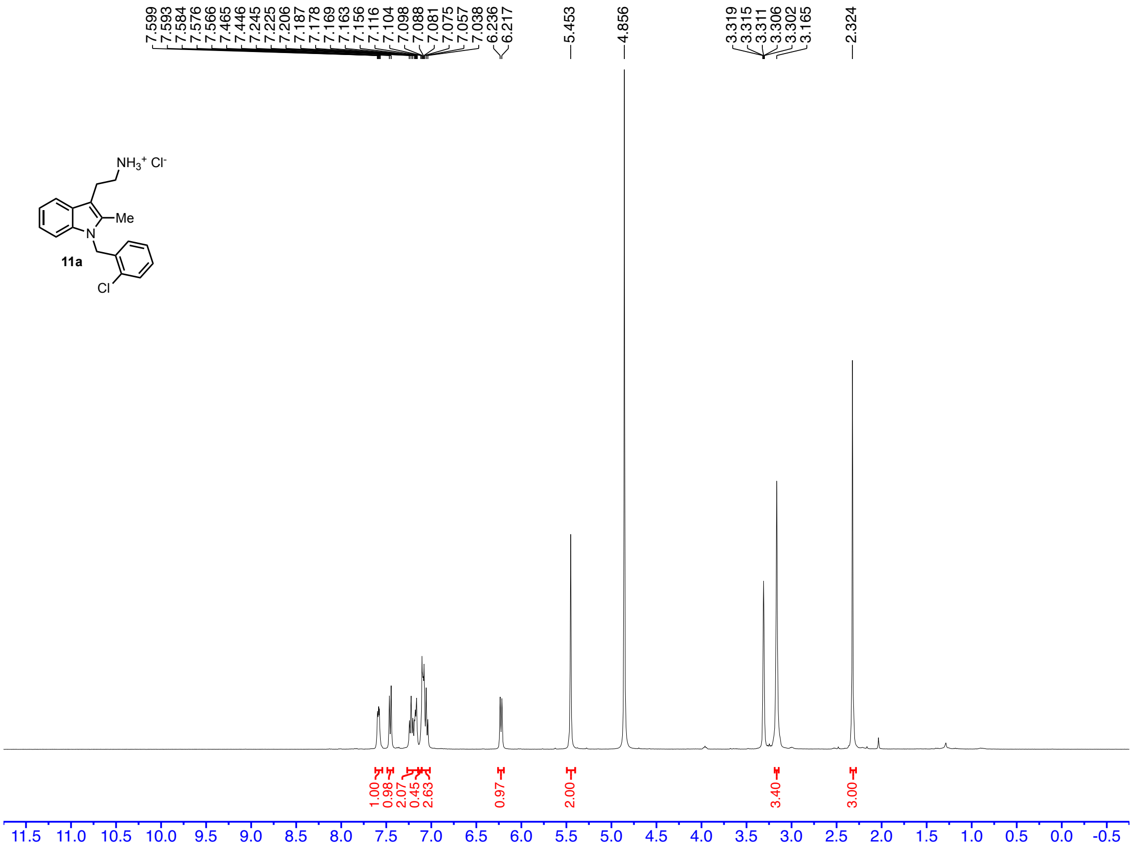

STM-3-26 - 1H NMR - 400.13 MHz - MeOD

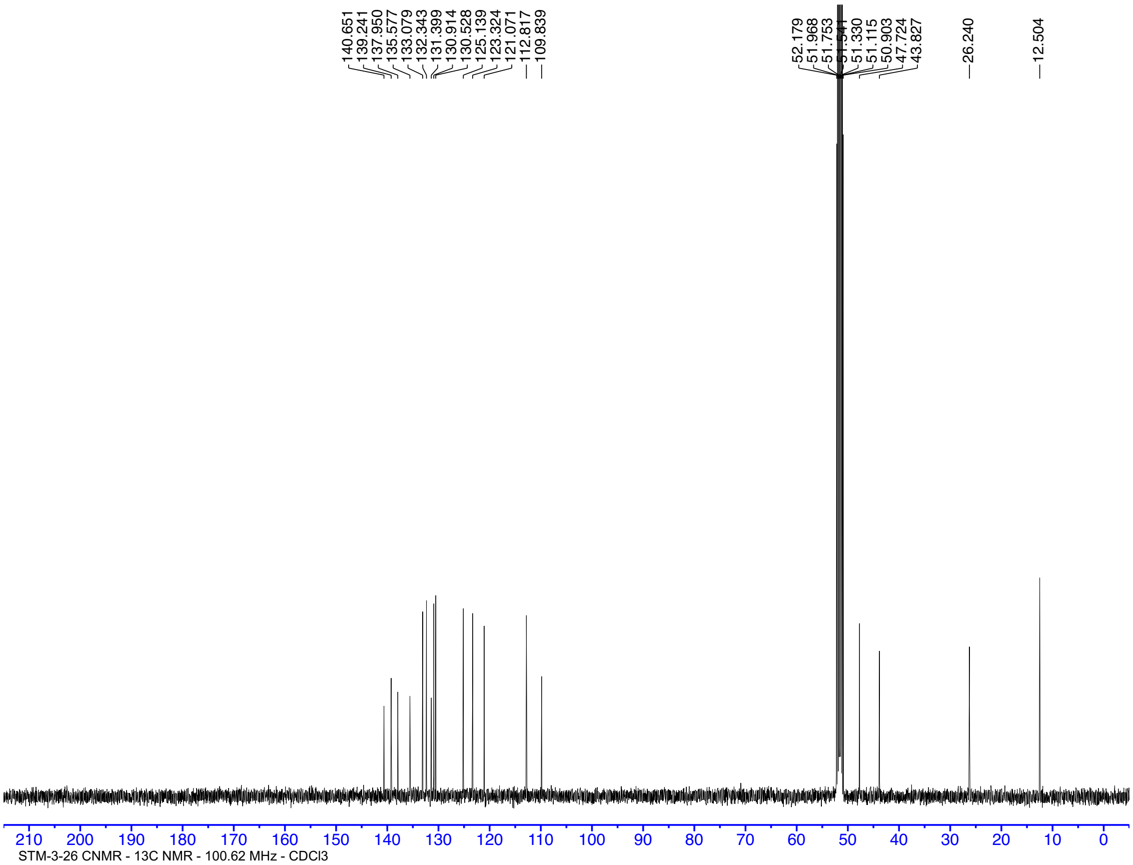

STM-3-26 CNMR - 13C NMR - 100.62 MHz - CDCl3

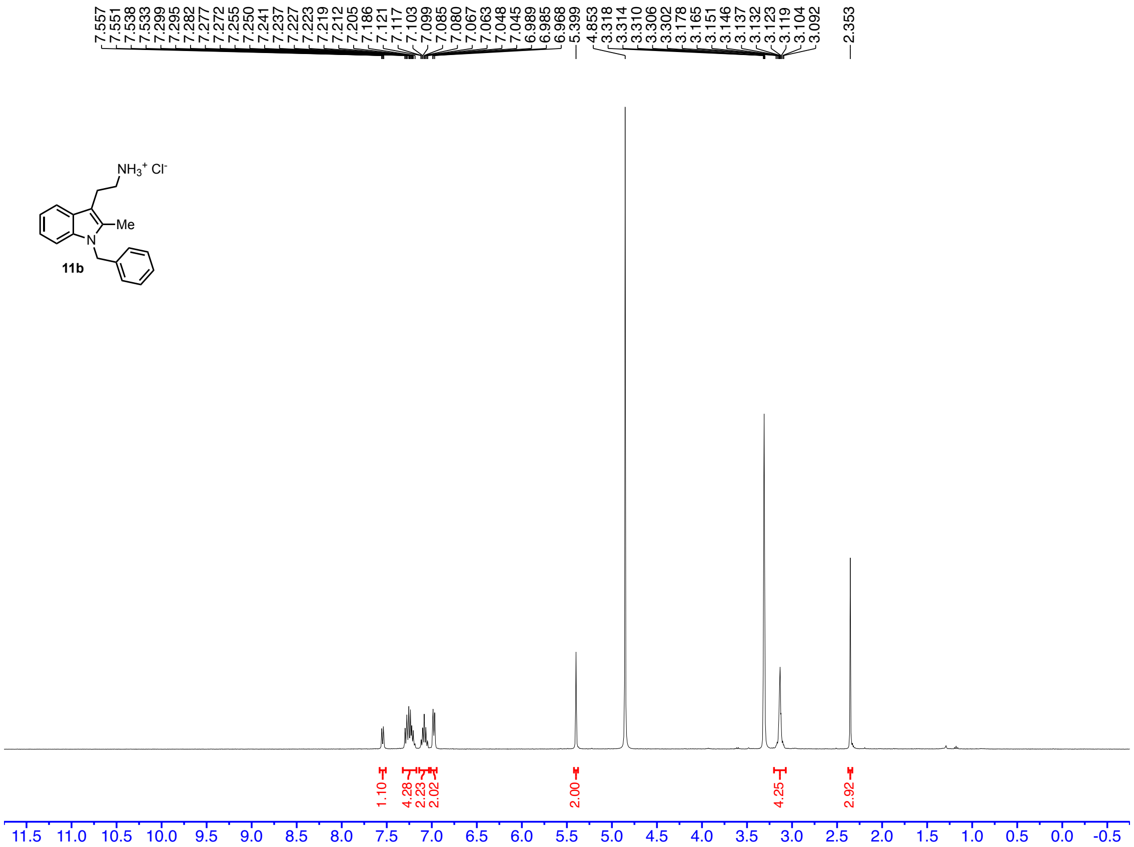

STM-3-31 - 1H NMR - 400.13 MHz - MeOD

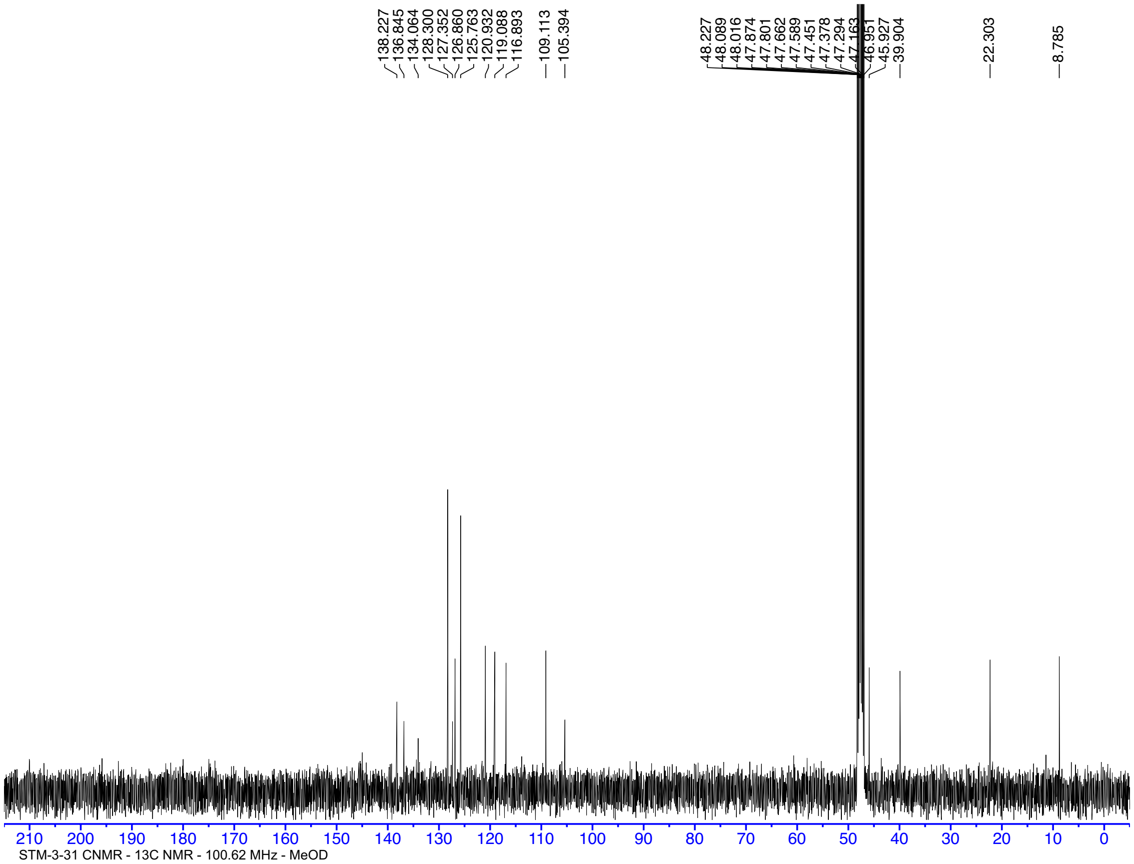

STM-3-31 CNMR - 13C NMR - 100.62 MHz - MeOD

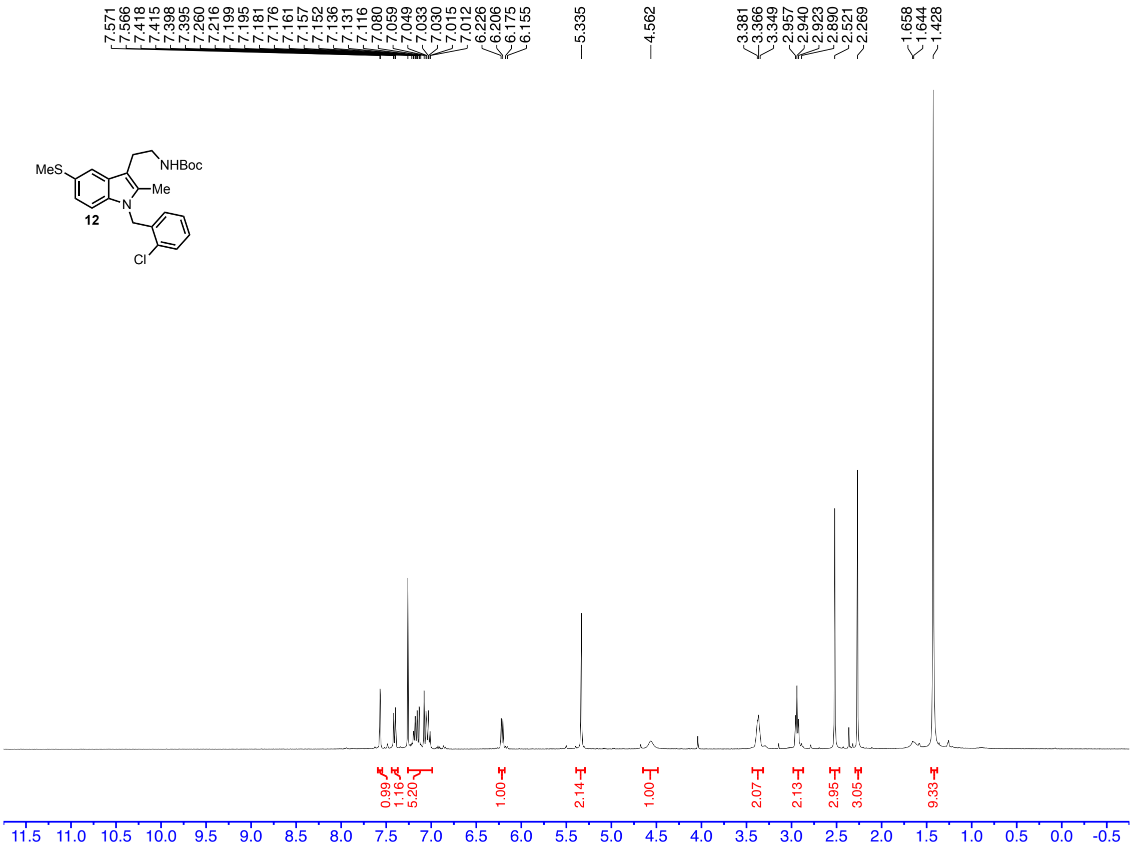

STM-3-15 - 1H NMR - 400.13 MHz - CDCl3

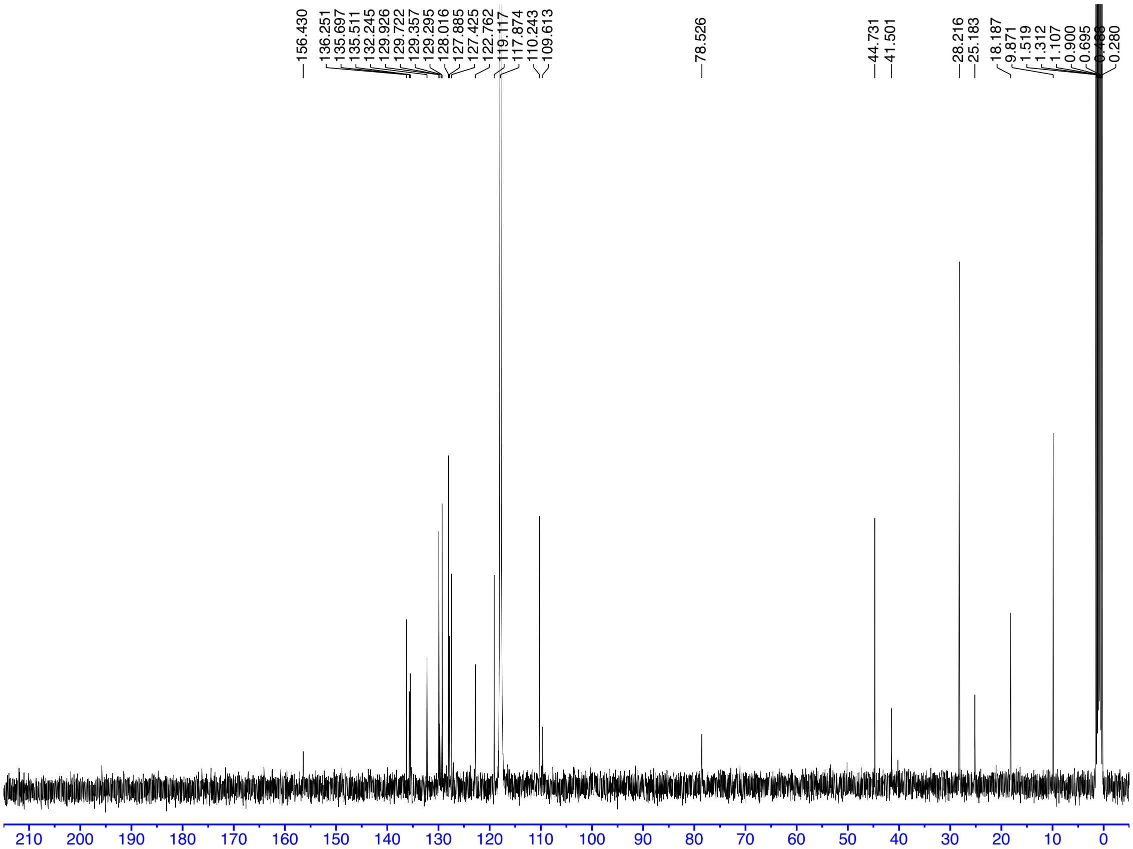

STM-3-15 CNMR MeCN - 13C NMR - 100.62 MHz - CD3CN

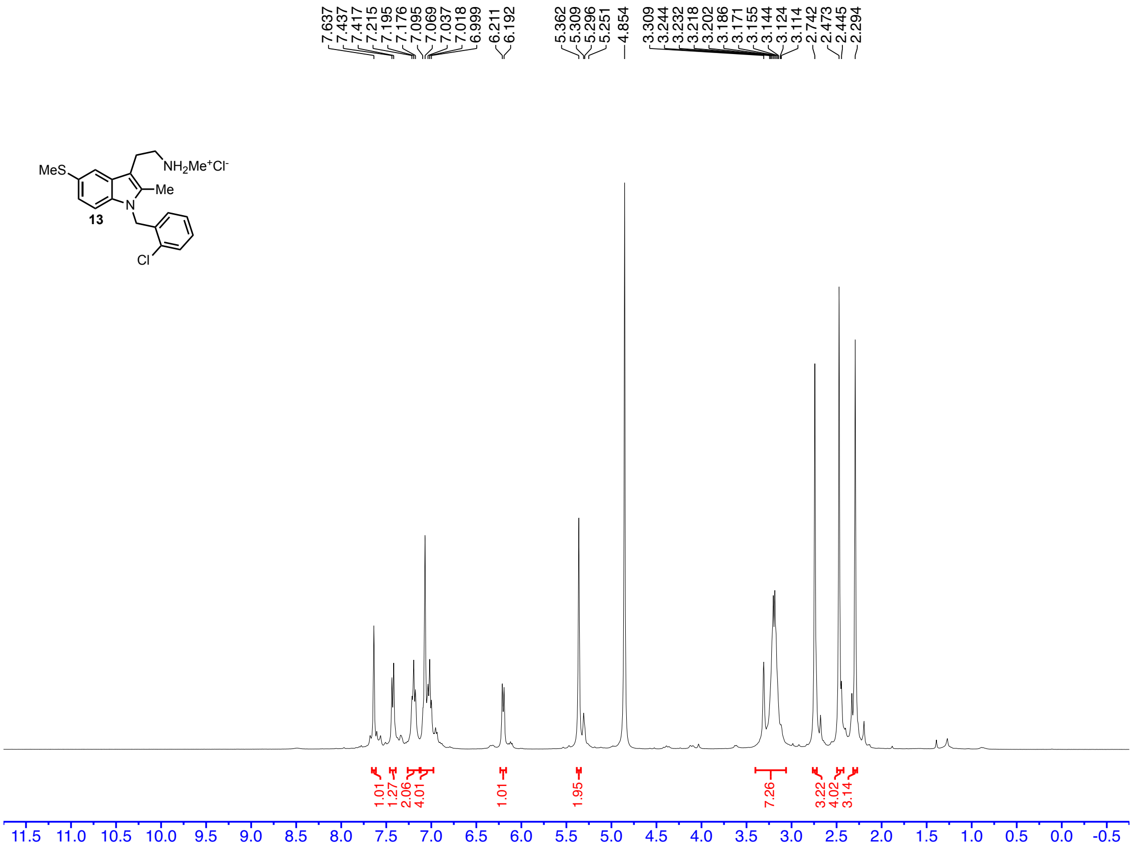

STM-3-39 Solvent Removal - 1H NMR - 400.13 MHz - MeOD

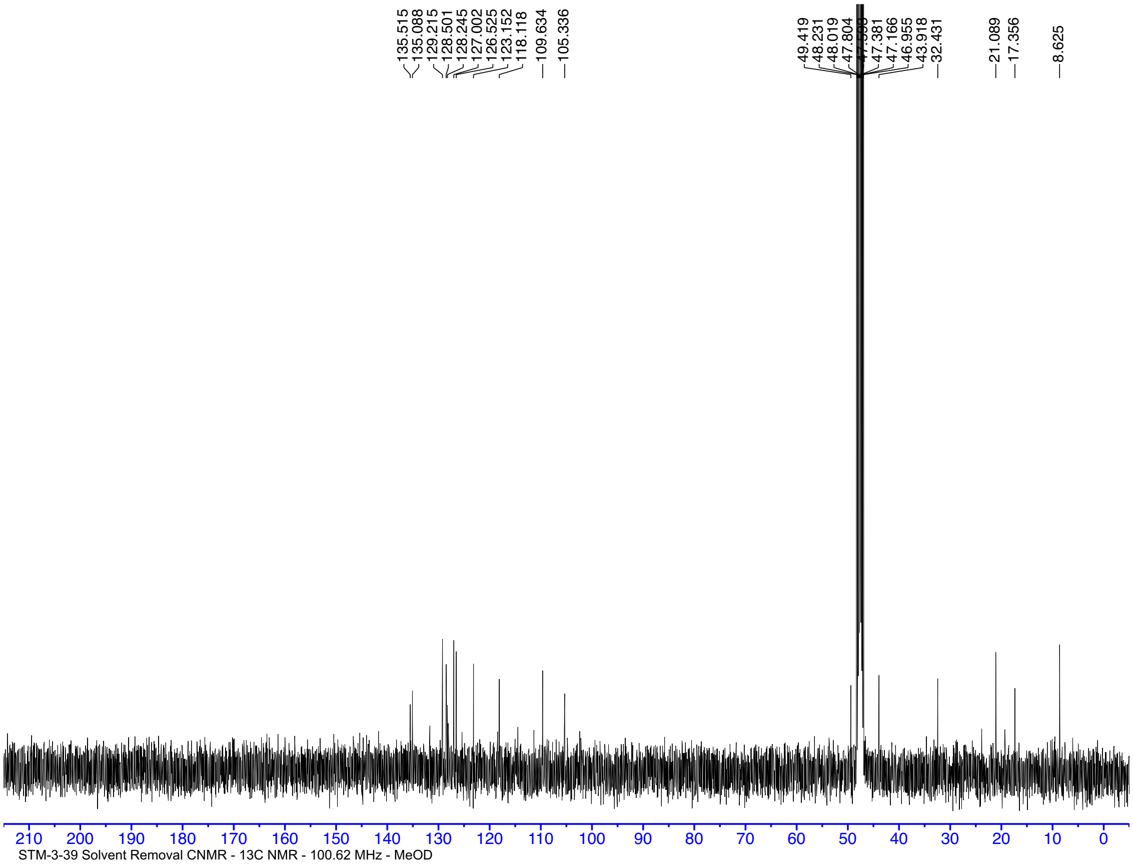

STM-3-39 Solvent Removal CNMR - 13C NMR - 100.62 MHz - MeOD

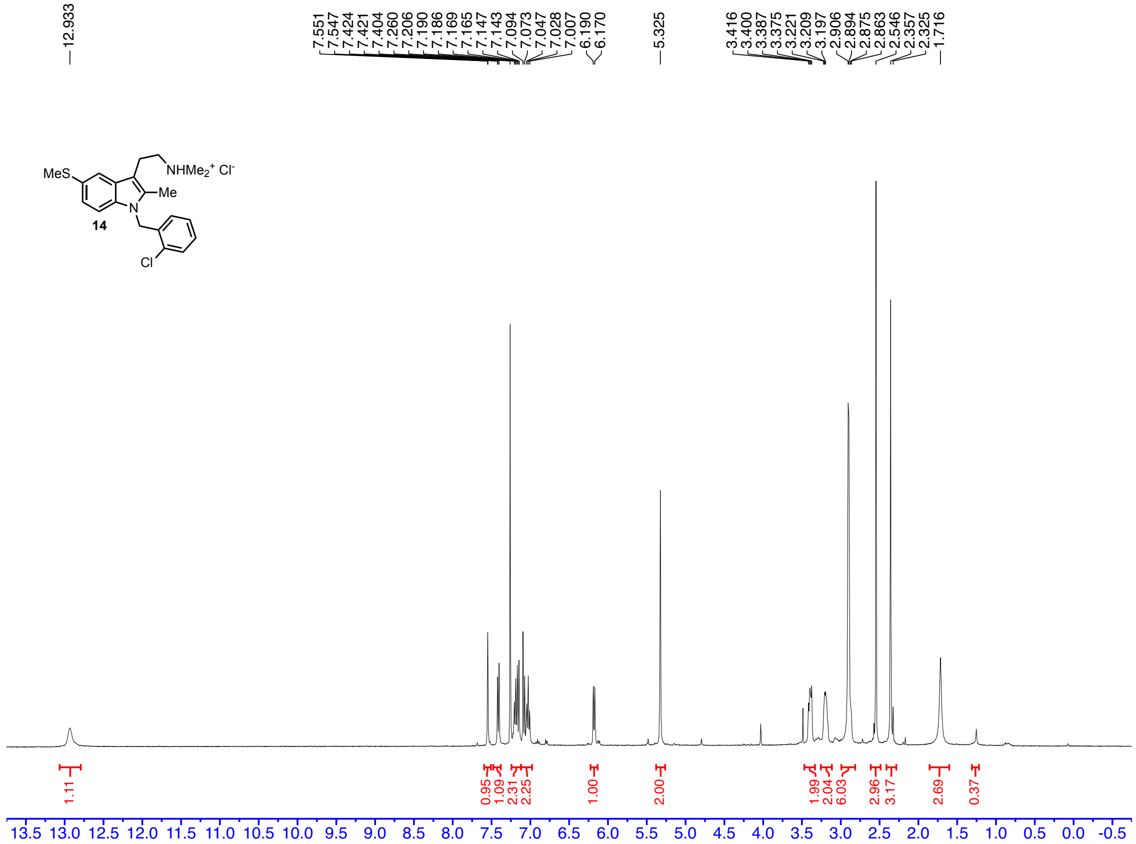

STM-3-21 CDCl3 - 1H NMR - 400.13 MHz - CDCl3

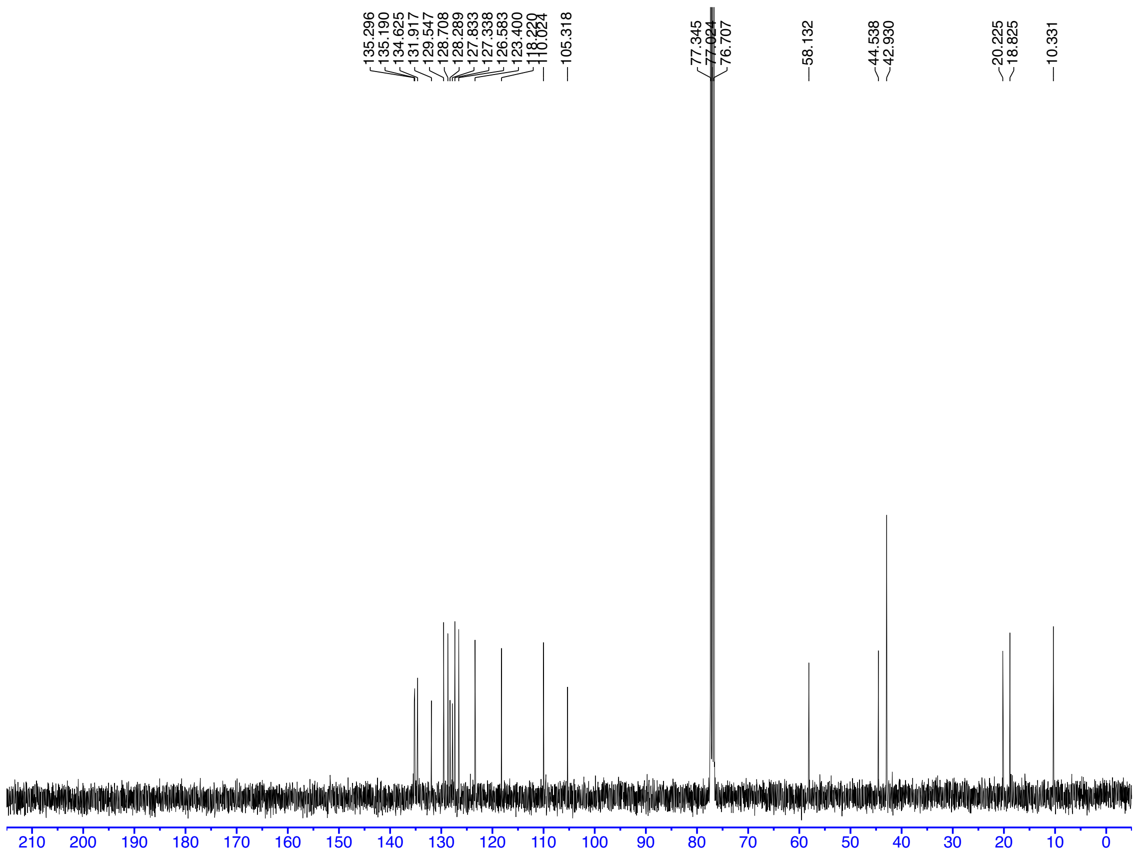

STM-3-21 CNMR concentrated - 13C NMR - 100.62 MHz - CDCl3

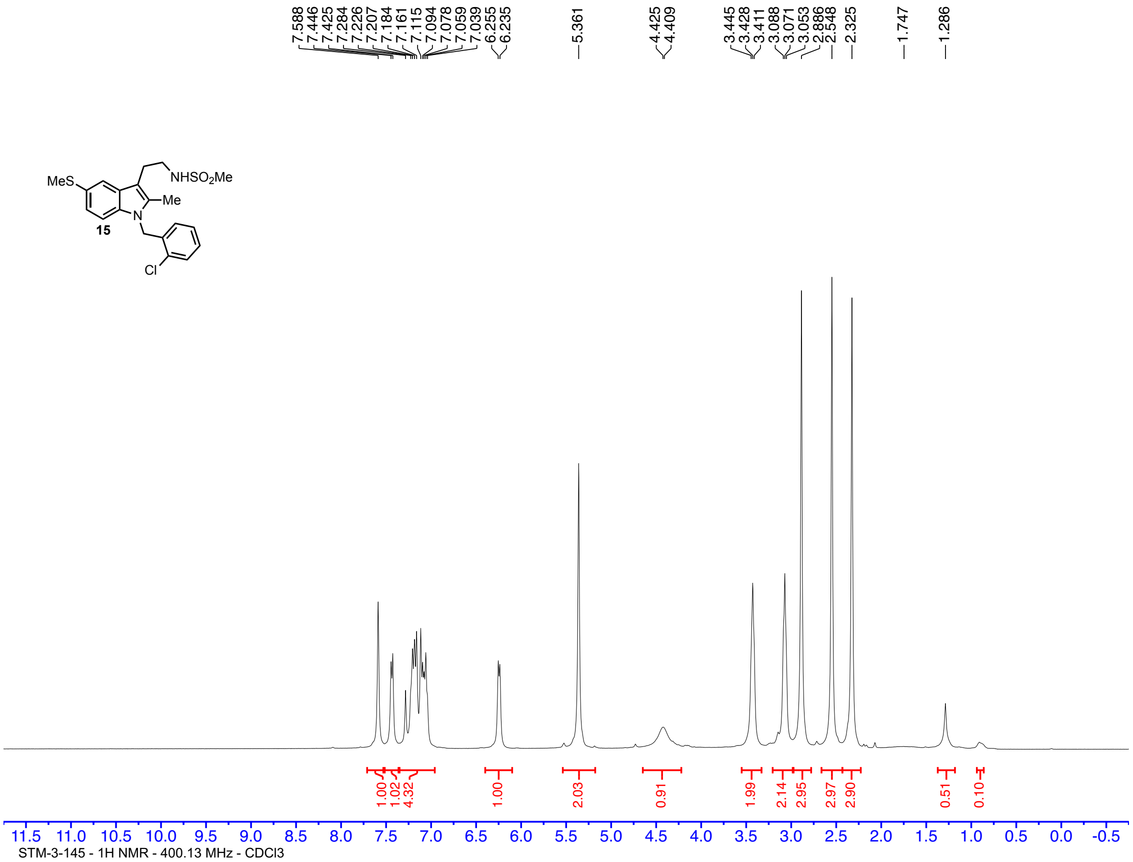

STM-3-145 - 1H NMR - 400.13 MHz - CDCl3

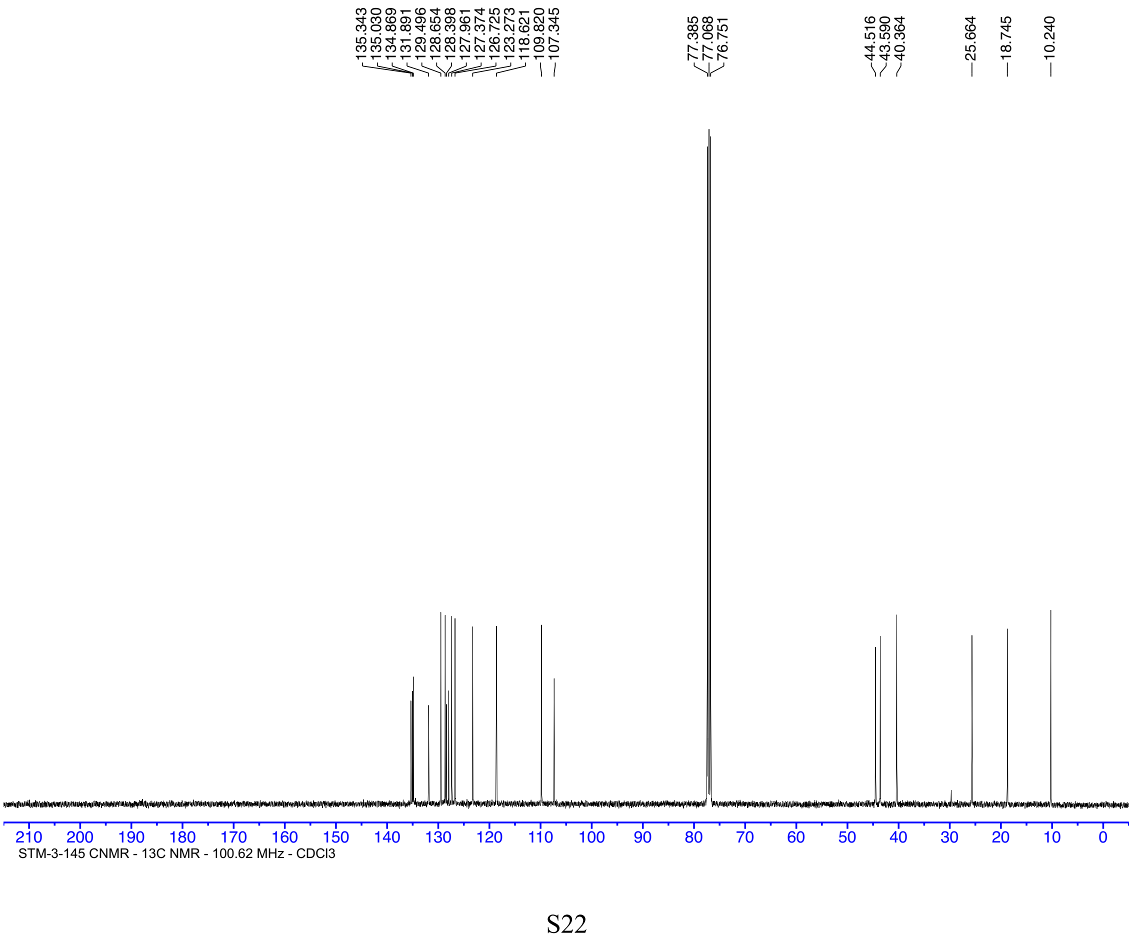

STM-3-145 CNMR - 13C NMR - 100.62 MHz - CDCl3

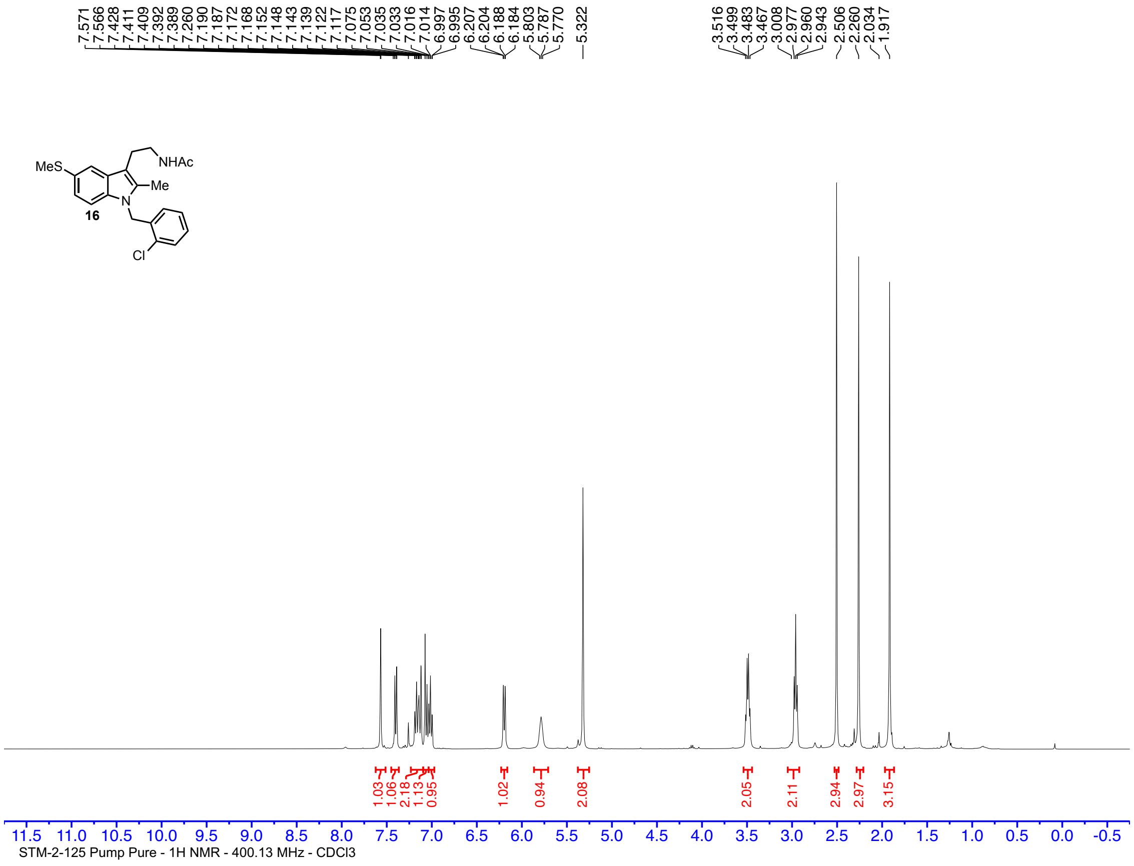
7.571
7.566
7.428
7.411
7.409
7.392
7.389
7.260
7.190
7.187
7.172
7.168
7.152
7.148
7.143
7.139
7.122
7.117
7.075
7.053
7.035
7.033
7.016
7.014
6.997
6.995
6.207
6.204
6.188
6.184
5.803
5.787
5.770
5.322
3.516
3.499
3.483
3.467
3.008
2.977
2.960
2.943
2.506
2.260
2.034
1.917
MeS
NHAc
Me
N
16
Cl
1.03
1.06
2.18
1.13
0.95
1.02
0.94
2.08
2.05
2.11
2.94
2.97
3.15
11.5 11.0 10.5 10.0 9.5 9.0 8.5 8.0 7.5 7.0 6.5 6.0 5.5 5.0 4.5 4.0 3.5 3.0 2.5 2.0 1.5 1.0 0.5 0.0 -0.5
STM-2-125 Pump Pure - 1H NMR - 400.13 MHz - CDCl3

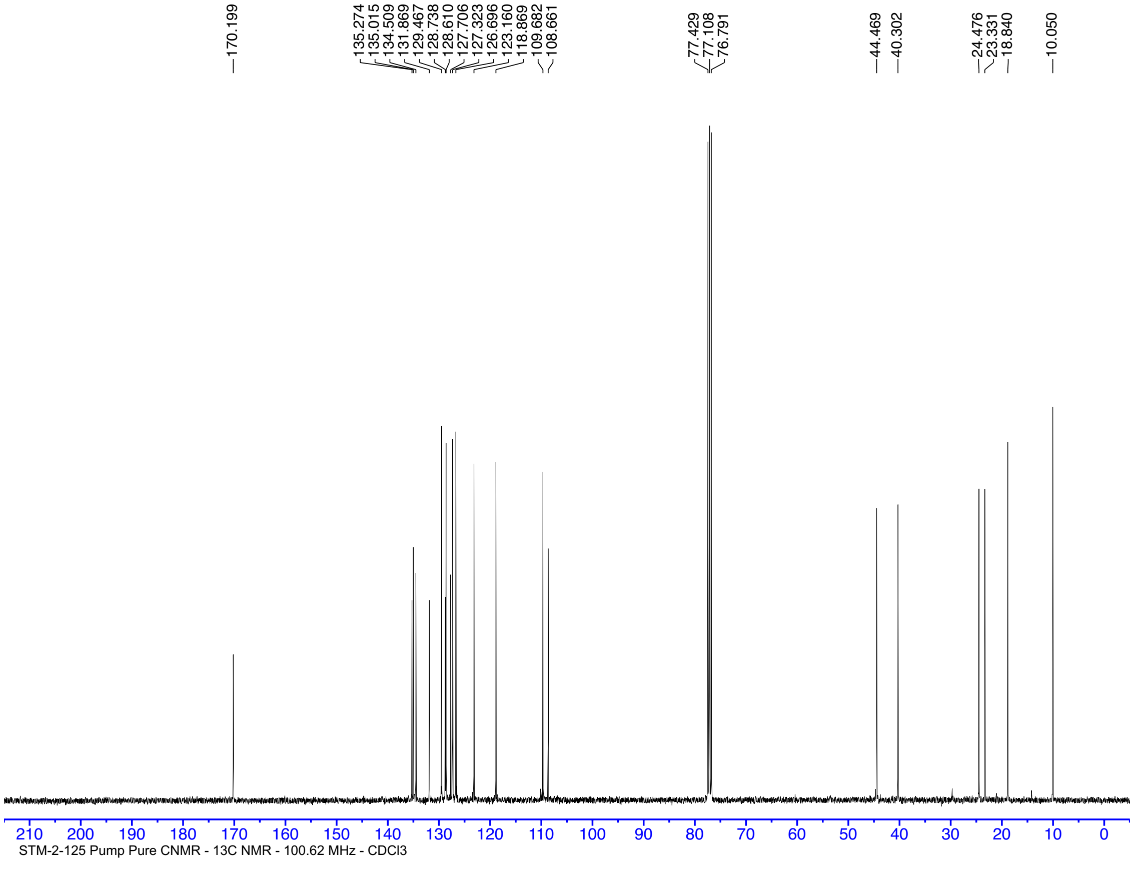
170.199
135.274
135.015
134.509
131.869
129.467
128.738
128.610
127.706
127.323
126.696
123.160
118.869
109.682
108.661
77.429
77.108
76.791
44.469
40.302
24.476
23.331
18.840
10.050
210 200 190 180 170 160 150 140 130 120 110 100 90 80 70 60 50 40 30 20 10 0
STM-2-125 Pump Pure CNMR - 13C NMR - 100.62 MHz - CDCl3

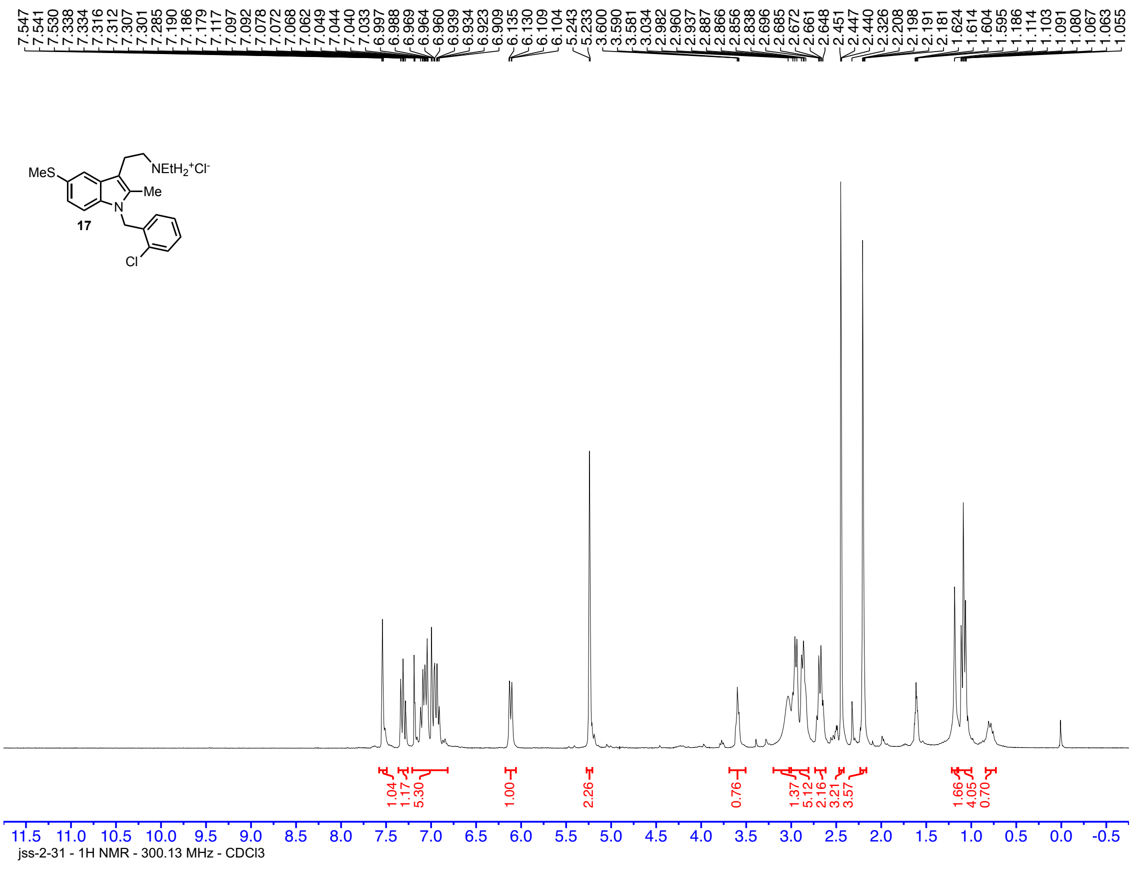

jss-2-31 - 1H NMR - 300.13 MHz - CDCl3

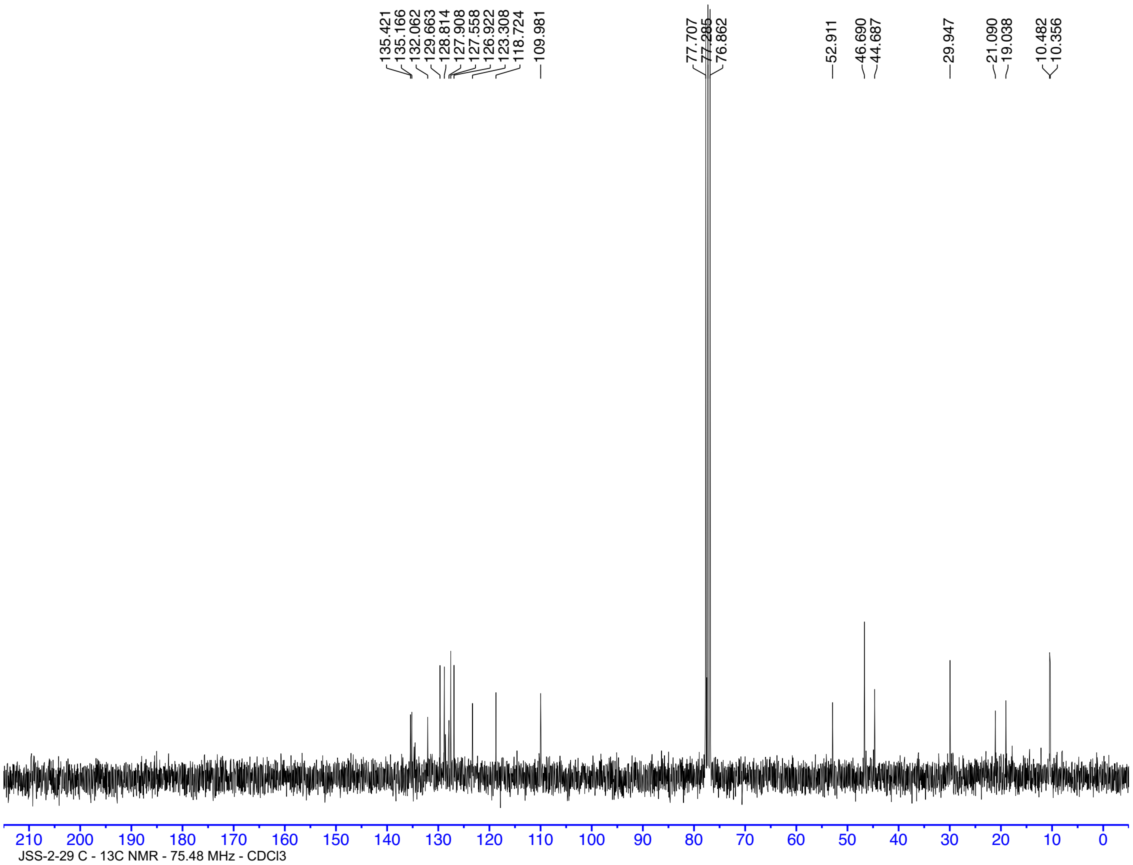

JSS-2-29 C - 13C NMR - 75.48 MHz - CDCl3

Table S1. Predicted Physiochemical Properties and Lipophilicity for the Tested Tryptamines[a]

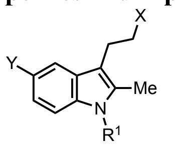

Entry	Cmpd	R1	X	Y	MW	CLogP[b]	TPSA	Molar Refractivity	Number of Rotatable Bonds	H Bond Acceptors	H Bond Donors
1	**1**	2-chlorobenzyl	NH_3^+ Cl^-	SMe	344.9	4.11	56.25	101.86	5	1	1
2	**7b**	4-chlorobenzyl	NH_3^+ Cl^-	SMe	344.9	4.11	56.25	101.86	5	1	1
3	**7c**	4-bromobenzyl	NH_3^+ Cl^-	SMe	389.35	4.23	56.25	104.55	5	1	1
4	**7d**	2,4-dichlorobenzyl	NH_3^+ Cl^-	SMe	379.35	4.60	56.25	106.87	5	1	1
5	**7e**	benzyl	NH_3^+ Cl^-	SMe	310.46	2.10	56.25	96.85	5	1	1
6	**7f**	H	NH_3^+ Cl^-	SMe	220.33	3.62	67.11	67.47	3	1	2
7	**11a**	benzyl	NH_3^+ Cl^-	H	264.36	3.12	30.95	85.13	4	1	1
8	**11b**	2-chlorobenzyl	NH_3^+ Cl^-	H	298.81	3.62	30.95	90.14	4	1	1
9	**13**	2-chlorobenzyl	$MeNH_2^+$ Cl^-	SMe	358.93	4.34	42.26	106.77	6	1	1
10	**14**	2-chlorobenzyl	Me_2NH^+ Cl^-	SMe	372.95	4.56	33.47	111.67	6	1	0
11	**15**	2-chlorobenzyl	$NHSO_2Me$	SMe	422.99	3.27	84.78	115.73	7	3	1
12	**16**	2-chlorobenzyl	NHAc	SMe	386.94	4.01	59.33	111.77	7	1	1
13	**17**	2-chlorobenzyl	$EtNH_2^+$ Cl^-	SMe	372.95	4.56	42.26	111.57	7	1	1

[a]Calculated using SwissADME.[1] [b]CLogP calculated for the free amine using the MLOGP method[2, 3] with SwissADME.[1]

Table S2. Predicted Pharmacokinetic Properties for the Tested Tryptamines[a]

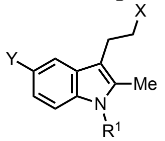

Entry	Cmpd	R1	X	Y	ESOL Log S	ESOL Solubility class	GI absorption	BBB permeant	Pgp substrate	CYP1A2 inhibitor	CYP2C19 inhibitor	CYP2C9 inhibitor	log Kp (cm/s)
1	**1**	2-chlorobenzyl	NH_3^+ Cl^-	SMe	-4.93	Moderately soluble	High	Yes	Yes	Yes	Yes	Yes	-5.24
2	**7b**	4-chlorobenzyl	NH_3^+ Cl^-	SMe	-4.93	Moderately soluble	High	Yes	Yes	Yes	Yes	Yes	-5.24
3	**7c**	4-bromobenzyl	NH_3^+ Cl^-	SMe	-5.25	Moderately soluble	High	Yes	Yes	Yes	Yes	Yes	-5.47
4	**7d**	2,4-dichlorobenzyl	NH_3^+ Cl^-	SMe	-5.52	Moderately soluble	High	Yes	Yes	Yes	Yes	Yes	-5.01
5	**7e**	benzyl	NH_3^+ Cl^-	SMe	-4.35	Moderately soluble	High	Yes	Yes	Yes	Yes	No	-5.47
6	**7f**	H	NH_3^+ Cl^-	SMe	-2.89	Soluble	High	Yes	No	Yes	No	No	-6.03
7	**11a**	benzyl	NH_3^+ Cl^-	H	-3.86	Soluble	High	Yes	Yes	Yes	Yes	No	-5.56
8	**11b**	2-chlorobenzyl	NH_3^+ Cl^-	H	-4.44	Moderately soluble	High	Yes	Yes	Yes	Yes	No	-5.33
9	**13**	2-chlorobenzyl	$MeNH_2^+$ Cl^-	SMe	-5.26	Moderately soluble	High	Yes	Yes	Yes	Yes	Yes	-4.96
10	**14**	2-chlorobenzyl	Me_2NH^+ Cl^-	SMe	-5.62	Moderately soluble	High	Yes	No	Yes	Yes	Yes	-4.72
11	**15**	2-chlorobenzyl	$NHSO_2Me$	SMe	-5.25	Moderately soluble	High	No	No	Yes	Yes	Yes	-5.69
12	**16**	2-chlorobenzyl	NHAc	SMe	-5.17	Moderately soluble	High	Yes	No	Yes	Yes	Yes	-5.32
13	**17**	2-chlorobenzyl	$EtNH_2^+$ Cl^-	SMe	-5.49	Moderately soluble	High	Yes	Yes	Yes	Yes	No	-4.79

[a]Calculated using SwissADME.[1]

References

1. Daina, A.; Michielin, O.; Zoete, V.; Michielin, O.; Michielin, O. SwissADME: a free web tool to evaluate pharmacokinetics, drug-likeness and medicinal chemistry friendliness of small molecules. *Sci Rep* **2017,** *7*, 42717. doi:10.1038/srep42717
2. Moriguchi, I.; Hirono, S.; Nakagome, I.; Hirano, H. Comparison of reliability of log P values for drugs calculated by several methods. *Chem. Pharm. Bull.* **1994,** *42*, 976. doi:10.1248/cpb.42.976
3. Lipinski, C. A.; Lombardo, F.; Dominy, B. W.; Feeney, P. J. Experimental and computational approaches to estimate solubility and permeability in drug discovery and development settings. *Adv. Drug Delivery Rev.* **1997,** *23*, 3-25. doi:10.1016/s0169-409x(00)00129-0
